# Computational insights into the mutagenicity of two tobacco-derived carcinogenic DNA lesions

**DOI:** 10.1093/nar/gky1071

**Published:** 2018-11-08

**Authors:** Katie A Wilson, Josh L Garden, Natasha T Wetmore, Stacey D Wetmore

**Affiliations:** Department of Chemistry and Biochemistry, University of Lethbridge, 4401 University Drive West, Lethbridge, Alberta T1K 3M4, Canada

## Abstract

4-(methylnitrosamino)-1-(3-pyridyl)-1-butanone is a potent carcinogen found in all tobacco products that leads to a variety of DNA lesions in cells, including O6-[4-oxo-4-(3-pyridyl)butyl]guanine (POB-G) and O6-[4-hydroxy-4-(3-pyridyl)butyl]guanine (PHB-G), which differ by only a single substituent in the bulky moiety. This work uses a multiscale computational approach to shed light on the intrinsic conformational and base-pairing preferences of POB-G and PHB-G, and the corresponding properties in DNA and the polymerase η active site. Our calculations reveal that both lesions form stable pairs with C and T, with the T pairs being the least distorted relative to canonical DNA. This rationalizes the experimentally reported mutational profile for POB-G and validates our computational model. The same approach predicts that PHB-G is more mutagenic than POB-G due to a difference in the bulky moiety hydrogen-bonding pattern, which increases the stability of the PHB-G:T pair. The mutagenicity of PHB-G is likely further increased by stabilization of an intercalated DNA conformation that is associated with deletion mutations. This work thereby uncovers structural explanations for the reported mutagenicity of POB-G, provides the first clues regarding the mutagenicity of PHB-G and complements a growing body of literature highlighting that subtle chemical changes can affect the biological outcomes of DNA adducts.

## INTRODUCTION

Nitrosamines are a large group of compounds that occur in the human diet, cosmetics, and tobacco, as well as flexible plastics such as balloons, condoms and baby bottle nipples (1–3). Despite the high prevalence of nitrosamines in contemporary society, these compounds are very carcinogenic, with 90% of the 300 identified nitrosamines being known carcinogens (4). The nitrosamine 4-(methylnitrosamino)-1-(3-pyridyl)-1-butanone (NNK) is formed during the curing of tobacco and has been identified in all tobacco products, including those used in conventional cigarettes, electronic cigarettes and Hookahs (5–7). NNK is also the only tobacco component that has led to lung cancer in every species tested (i.e. mice, rats, hamsters, rabbits, pigs, monkeys and humans) regardless of the route of administration (8,9). Therefore, the World Health Organization’s International Agency for Research on Cancer has classified NNK as carcinogenic to humans (Group 1) (10). Nevertheless, changes in cigarette design since the 1950s and new routes of tobacco administration have increased human exposure to NNK, and consequently led to an increase in the number of cases of adenocarcinoma (a form of lung cancer) (11,12). Indeed, lung cancer is the most common cancer caused by NNK and is also the leading cause of cancer death worldwide in part due to a survival rate of only 18% (13).

**Figure 1.**
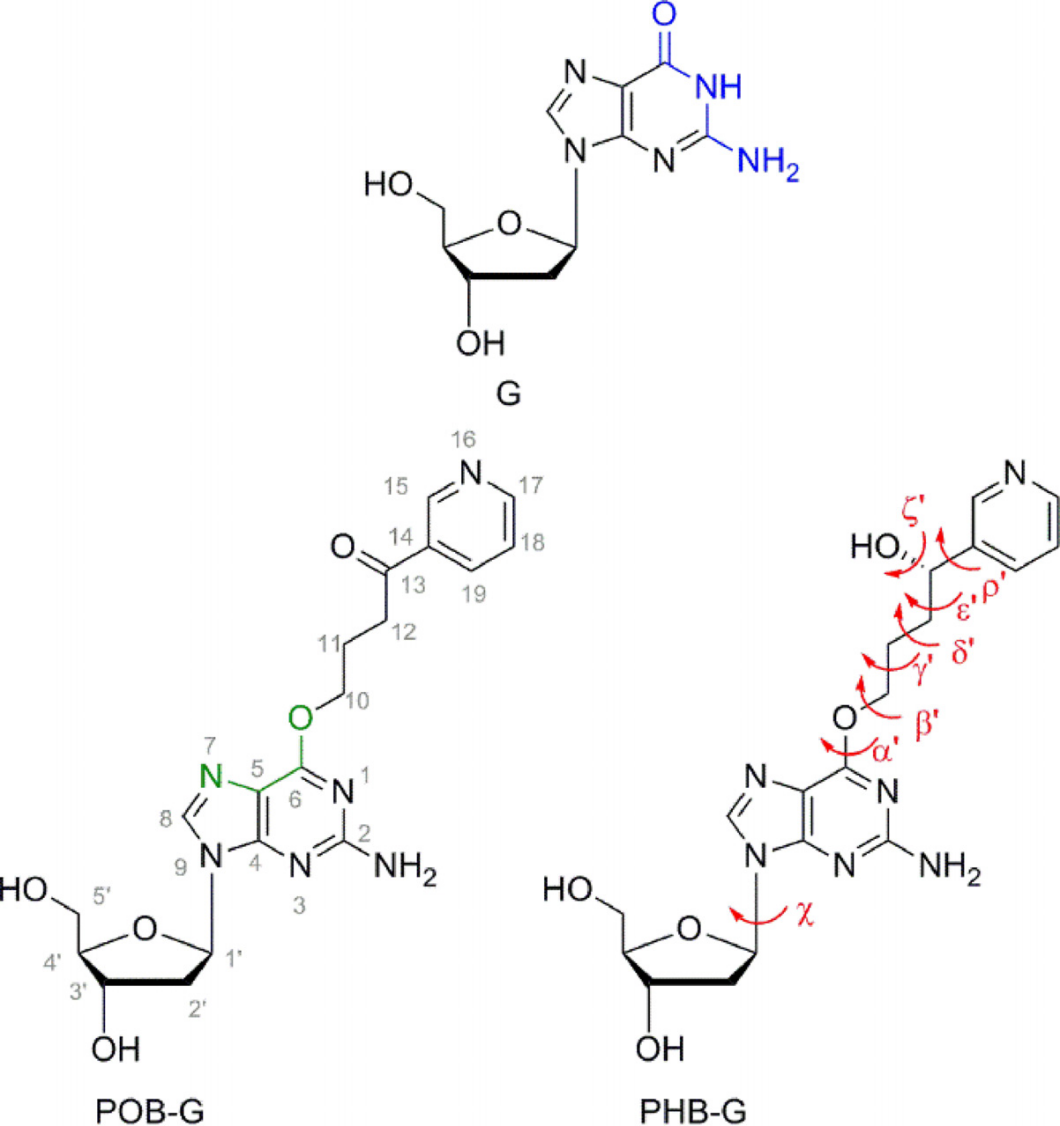
The G, POB-G and PHB-G nucleosides in the *anti* glycosidic conformation, with the Watson–Crick hydrogen-bonding face highlighted in blue and the Hoogsteen face in green. Key dihedral angles are defined for the lesions including χ [∠(O4′C1′N9C4)], α′ [∠(C5C6O6C10)], β′ [∠(C6O6C10C11)], γ′ [∠(O6C10C11C12)], δ′ [∠(C10C11C12C13)], ε′ [∠(C11C12C13C14)] and ρ′ [∠(C12C13C14C15)], as well as ζ′ for PHB-G [∠(C12C13O13H13)].

In cells, NNK can be reduced to 4-(methylnitrosamino)-1-(3-pyridyl)-1-butanol (NNAL), and cytochrome p450 enzymes convert both NNK and NNAL into species that react with DNA (5). The complex metabolism of NNK and NNAL gives rise to a variety of DNA lesions including methyl, formaldehyde, pyridyloxobutyl (POB) and pyridylhydroxybutyl (PHB) DNA adducts (5). The current study focuses on the larger POB-G and PHB-G lesions that result from attack at the O6 position of G (Figure 1). Both POB-G and PHB-G have a modified Watson–Crick hydrogen-bonding face compared to G (i.e. N1 becomes a hydrogen-bond acceptor; Figure 1). Therefore, this may cause these G lesions to no longer exhibit a preference for C, which could result in mutations upon replication. POB-G and PHB-G differ in the substitution of the chain connecting the adducted G to the bulky moiety ring (i.e. carbonyl versus hydroxyl group, respectively) and previous work on DNA adducts arising from known human carcinogens has shown that lesion mutagenicity is affected by small changes in chemical composition including linker substituents, ring substitution and ionization state (14–25). Thus, POB-G and PHB-G may have different mutagenic profiles, and their consideration may afford a broader understanding of the effect of bulky moiety linker composition on lesion mutagenicity.

Detailed investigations have shown that POB-G is highly mutagenic (21,26,27). Specifically, POB-G has a 96% mutation rate in *Escherichia coli* cells, exclusively leading to G → A transition mutations (26). The mutational spectrum in human HEK293 cells is slightly more complex, with the primary replication outcome still being G → A transition mutations (54%), but G → T mutations also occurring at a low frequency (2%) (26). *In vitro* kinetics studies support these reported mutational spectra, indicating that translesion synthesis (TLS) polymerases preferentially insert dCTP and dTTP opposite the lesion (21,27). Although the kinetic studies also report dGTP insertion, extension past the POB-G:G base pair does not occur (21,27). Furthermore, while polymerase (pol) η bypasses POB-G with a *k*_cat_/*K*_m_ of 0.63 mM^−1^ s^−1^, pol ι, κ and ν bypass POB-G with a *k*_cat_/*K*_m_ of < 0.005 mM^−1^ s^−1^, which indicates that pol η likely plays a critical role in POB-G replication (21,27). Nevertheless, POB-G replication is significantly slower compared to that of natural DNA, which has a *k*_cat_/*K*_m_ of 160 mM^−1^ s^−1^ for the insertion of dCTP opposite G by pol η (21). While the mutagenicity of PHB-G is currently unknown, the NNAL parent compound that primarily forms PHB lesions has been reported to be tumorigenic in mice (28).

To complement mutagenicity data, structural studies have been performed on POB-G using a variety of models (29–31). Molecular mechanics (MM) minimization of the 5′–AT[POB-G] 2′-deoxytrinucleoside diphosphate revealed that the bulky moiety can be directed either 3′ or 5′ with respect to the lesion site, or extended in the same plane as the adducted G (29). Classical molecular dynamics (MD) simulations on POB-G-adducted DNA (29) and nuclear magnetic resonance (NMR)-based restrained MM minimizations (30) found that the bulky moiety is preferentially extended in the same plane as the adducted G. Nevertheless, while only one position of the POB-G bulky moiety was reported in the NMR structure, the inherent flexibility of the lesion is reflected in broad NMR signals (30). This correlates with computational work on the POB-G nucleobase (31) and 5′–AT[POB-G] 2′-deoxytrinucleoside diphosphate models (29), which indicate POB-G has a high degree of conformational flexibility. In terms of base pairing, POB-G has been shown both in computational and experimental work to form a wobble pair opposite C (29–31). Previous computational work from our group provided the first structural data for understanding the observed POB-G mutagenicity by characterizing nucleobase base dimers (31). Specifically, a stable, non-distorted pseudo-Watson–Crick POB-G:T pair and marginally distorted pairs between the Watson–Crick face of POB-G and A or C were identified (31). Furthermore, a predicted stable, but highly-distorted, Hoogsteen POB-G:G pair was found (31), which correlates with the experimentally-observed insertion of dGTP opposite POB-G, but the lack of subsequent extension (21,27). Overall, these previous studies have provided insight into the flexibility and base-pairing propensity of POB-G. Furthermore, these works highlight the critical role computational studies can play in unveiling how lesion structural properties relate to mutagenicity. Nevertheless, structural details of the adduct mispairs within the helical environment, as well as pairs within the polymerase active site, are key for understanding lesion mutagenicity, but remain unavailable. Additionally, no structural studies have clarified the similarities and/or differences in the flexibility and base-pairing preferences of PHB-G with respect to POB-G.

To shed further light on the mutagenicity of POB-G and provide the first clues regarding the mispairing potential of PHB-G, the present work uses quantum chemical calculations and MD simulation methods to characterize the intrinsic conformational and hydrogen-bonding preferences of POB-G and PHB-G, as well as the corresponding properties in a DNA duplex and human TLS polymerase active site. Specifically, density functional theory (DFT) nucleobase and nucleoside models are initially used to examine the energy penalty for rotation about key bonds in the bulky moiety and at the glycosidic linkage, which uncovers the accessible conformations of free POB-G and PHB-G. Subsequently, 28 hydrogen-bonded pairs for each lesion were characterized using DFT nucleobase models to understand possible interactions between different lesion orientations and the canonical DNA nucleobases. MD simulations on adducted DNA provide insight into the dynamic conformational landscape of POB-G and PHB-G, and the effects of the identity of the pairing base on the structure of post-replication duplexes. Finally, MD simulations reveal the POB-G and PHB-G conformational flexibility and base pairing within the confines of the pol η active site, and thereby shed light on the relative propensity for the insertion of various dNTPs. Overall, our multiscaled computational approach provides structural insight into the mispairing potential of two key tobacco-derived DNA lesions and complements a growing body of literature focused on understanding how small changes in the chemical composition of adducts affect mutagenicity (14–25).

## MATERIALS AND METHODS

An AMBER conformational search about key rotatable bonds in the POB-G and PHB-G nucleobases (α′, β′, γ′, δ′, ε′, ρ′ and ζ′, Figure 1) as implemented in Hyperchem was used to determine the inherent conformational preference about the nucleobase–bulky moiety linker and within the bulky moiety. All orientations of POB-G and PHB-G isolated from the conformational search were subsequently optimized using DFT, specifically B3LYP-D3(BJ)/6–31G(d) and the relative energies were determined using B3LYP-D3/6–311+G(2df,2p). The structures thus obtained were grouped into conformational categories based on the orientation of the bulky moiety with respect to the adducted G. Subsequently, to assess the relative stability of the *anti* (χ ≈ 220°) and *syn* (χ ≈ 60°) conformations about the glycosidic bond in the POB-G and PHB-G nucleosides, 2′-deoxyribose was added to the most stable orientation from each conformational category of the nucleobase adducts. Finally, hydrogen-bonded nucleobase dimers between the Watson–Crick or Hoogsteen face of the most stable orientations of POB-G and PHB-G from each conformational category, and each of the four canonical DNA bases were investigated. These steps all involved B3LYP-D3(BJ)/6–31G(d) or M06–2X/6–31G(d) optimizations and B3LYP-D3(BJ)/6–311+G(2df,2p) energy calculations using Gaussian 09 (revision D.01).

MD simulations (AMBER14SB) were performed on 5′–CTCGGCG*CCATC 12-mer DNA duplexes, with G* = POB-G or PHB-G in orientations that represent each conformational category and paired opposite C. Using the insight gained from these simulations about possible POB-G or PHB-G orientations in DNA, MD simulations were performed on duplexes containing the lesions mispaired against T, A and G. Finally, MD simulations for dCTP, dTTP and dATP insertion opposite POB-G or PHB-G by pol η were initiated from a crystal structure of the insertion complex for dATP incorporation opposite T (PDB ID: 4ECS) (32). DNA systems were neutralized and solvated such that 8 Å of TIP3P water surround the duplex, while polymerase systems were solvated such that 10 Å of TIP3P water surround the solute and the concentration of NaCl is ∼0.150 M. All systems were minimized, heated and equilibrated. Subsequently, 20 ns pre-production simulations were performed to understand the inherent conformational dynamics at the lesion site. From these trial simulations, representative structures of unique conformations that cannot be easily converted into another conformation through standard MD sampling were chosen as starting points for further simulations. To enhance sampling of the accessible lesion conformations, this process was continued iteratively until no new structures were obtained. Based on these sampling simulations, the lesion orientation was used to select structures for three 100 ns replicas for each system (using different initial velocities) to ensure statistically relevant results were obtained. Due to negligible all-atom rmsds between each replica (generally < 2.5 Å; Supplementary Table S1), one replica was extended to yield a final 0.5 μs production trajectory for each DNA duplex and DNA–polymerase complex. Data from these final production simulations will be discussed throughout the main text. The representative structure for each MD simulation shown in the figures was obtained by clustering the trajectory based on the rmsd of the damaged base pair using the average linkage algorithm; however, all analysis was performed over the entire trajectory. To qualitatively compare the strength of lesion-site hydrogen bonding, the average lesion nucleobase pair interactions in DNA and polymerase models were estimated using B3LYP-D3(BJ)/6–311+G(2df,2p) calculations on 100 representative structures across each simulation.

Full details of the computational protocol are provided in the Supplementary Data.

## RESULTS AND DISCUSSION

### POB-G and PHB-G have a high degree of inherent conformational flexibility

**Figure 2.**
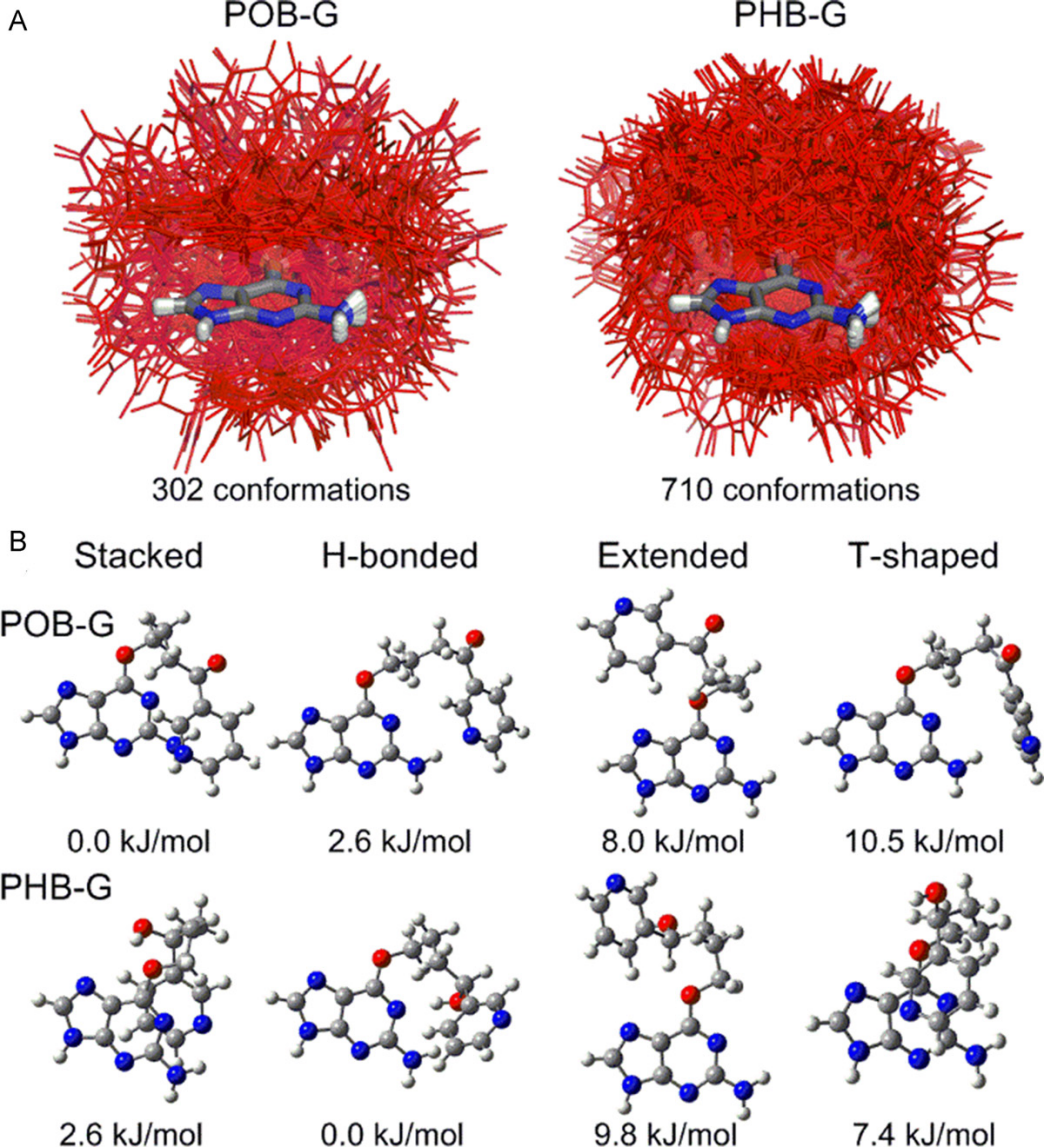
(**A**) Overlay (based on G ring atoms) of POB-G (left) and PHB-G (right) conformations identified using DFT, with the bulky moiety highlighted in red. (**B**) Most stable POB-G (top) and PHB-G (bottom) structures identified for each conformational category (relative energies provided). POB-G nucleobase geometries adapted from reference (31).

To understand the inherently preferred orientations of POB-G and PHB-G, the conformational landscape of both lesions is investigated using DFT nucleobase and nucleoside models. Consistent with the low rotational barriers previously reported for POB-G (31), both lesions are extremely flexible, with 302 and 710 unique conformations of POB-G and PHB-G identified, respectively (Figure 2A). The greater number of structures for PHB-G compared to POB-G arises due to multiple orientations of the hydroxy moiety. All structures were visually inspected and classified based on discrete interactions between the bulky moiety and adducted G as stacked, hydrogen bonded, T-shaped or extended (no interactions; Figure 2B). The majority of POB-G (62%) and PHB-G (52%) conformations do not contain an interaction between the bulky moiety and the adducted G (extended, Supplementary Figure S1). The relative distribution of the remaining conformations varies between the two adducts, although the separation between the categories is not as clear for PHB-G since some stacked conformations also involve hydrogen bonding between the bulky moiety hydroxy group and the adducted G. Most importantly, regardless of the lesion, substantial variation occurs within each conformational category (Figure 2A), which further highlights lesion flexibility.

The most stable POB-G conformation is stacked, while the most stable hydrogen bonded, extended and T-shaped orientations are 2.6, 8.0 and 10.5 kJ/mol less favourable, respectively (Figure 2B, top). Conversely, the most stable PHB-G conformation is hydrogen bonded, while the most stable stacked, T-shaped and extended orientations are 2.6, 7.4 and 9.8 kJ/mol less favourable, respectively (Figure 2B, bottom). Nevertheless, the PHB-G conformations span a larger energy range than those for POB-G (by 15 kJ/mol; Supplementary Figure S2). Upon adding 2′-deoxyribose to the most stable POB-G and PHB-G nucleobase conformations, the bulky moiety orientations and the trends in relative stability are maintained (Supplementary Figures S3 and 4). Additionally, the *anti* orientation is consistently ∼15 kJ/mol more stable than the *syn* conformation, which indicates that both glycosidic orientations of the lesions may be energetically accessible.

Overall, although our computational protocol may not have fully recovered all possible lesion conformations, the number and variation in the predicted orientations support the conclusions drawn from the nucleobase and nucleoside models. Most importantly, our calculations on the damaged nucleobases and nucleosides reveal that both POB-G and PHB-G display a high degree of inherent conformational flexibility. As a result, the nucleobase and nucleoside DFT models emphasize that many lesion conformations should be sampled in larger DNA and DNA–polymerase complexes, with observed conformations being limited by only the surrounding environment. Furthermore, each possible lesion conformation may have unique biological implications that should be carefully scrutinized in larger models.

### The Watson–Crick face of POB-G and PHB-G forms stable and minimally distorted base pairs with C, T and A, while the Hoogsteen face of POB-G and PHB-G forms stable but distorted base pairs with G

To understand the effects of the POB-G and PHB-G conformational heterogeneity on the lesion base-pairing potential, DFT is used to evaluate the geometry and strength of interactions between the Watson–Crick or Hoogsteen face of the most stable structure in each lesion conformational category and the canonical nucleobases. Additionally, since an NMR structure of POB-G adducted DNA predicted the bulky moiety to be projected straight into the major groove (30), the hydrogen-bonding potential of fully extended POB-G and PHB-G (i.e. all dihedral angles in the bulky moiety set to 180°) is also considered. Nevertheless, neither the POB nor PHB bulky moiety orientation affects the trends in stability of the resulting base pairs (Supplementary Table S2 and Figures S5–12). Therefore, only the fully extended conformation will be discussed for simplicity. However, the bulky moiety orientation leads to additional interactions between the lesion and pairing base in some cases (e.g. hydrogen bonding between the bulky moiety and opposing base; Supplementary Figures S5–12), which may be important and will be discussed in more detail in the helical and polymerase environments.

**Figure 3.**
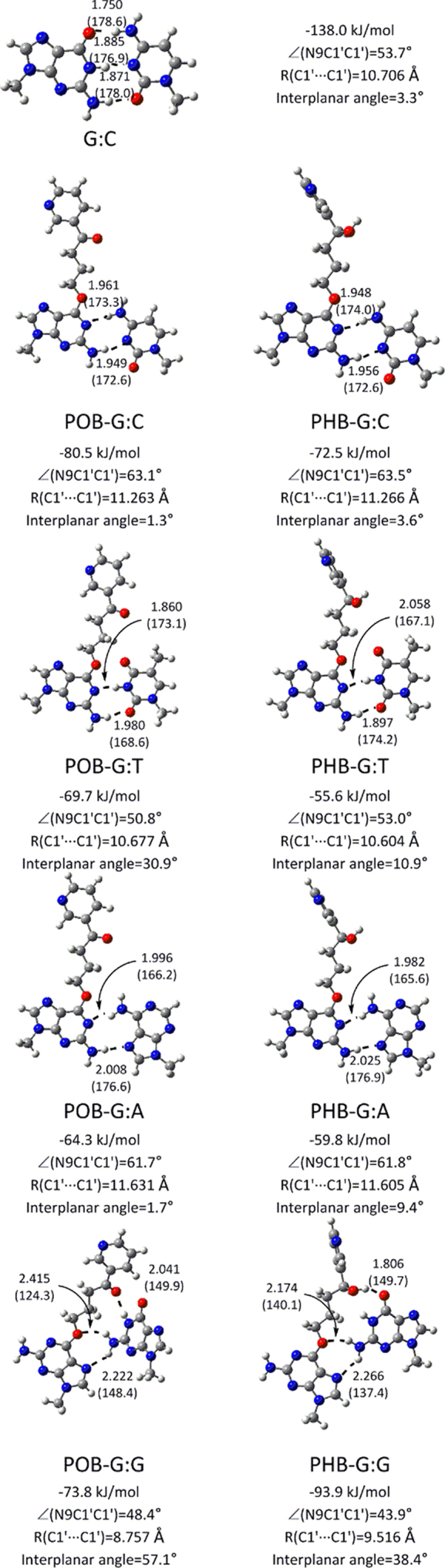
DFT structures (distances in Å and angles in deg.) and binding energies (kJ/mol) for the most stable hydrogen-bonded pairs between the extended POB-G (left) or PHB-G (right) conformation and each canonical nucleobase. Supplementary Figures S5–12 contain data for different lesion orientations. POB-G hydrogen-bonding dimer geometries adapted from reference (31).

Both POB-G and PHB-G form a wobble base pair with C using the Watson–Crick face of the lesion (Figure 3), which is consistent with previous studies on POB-G (29–31). This pair contains two hydrogen bonds and is weaker than the canonical G:C pair by ∼58–65 kJ/mol. Both wobble pairs are wider than G:C by ∼0.6 Å and the base pair opening is ∼10° larger. Nevertheless, the base pairs are nearly planar (interplanar angle = 1–4°). Regardless of the bulky moiety, adducted G forms a pseudo-Watson–Crick base pair with T (–69.7 or –55.6 kJ/mol for POB-G or PHB-G, respectively; Figure 3), which contains two hydrogen bonds. Although the G*:T mispairs slightly deviate from planarity (by 10–30°), both base pairs maintain the opening and width of natural DNA (50–54° and 10.6–10.7 Å, respectively), and therefore are overall less distorted than G*:C. Two hydrogen bonds are formed between the lesion Watson–Crick face and the Hoogsteen face of A (−64.3 or −59.8 kJ/mol for POB-G or PHB-G, respectively; Figure 3). The base pair opening and width of the G*:A pairs deviate from those of natural DNA (by ∼5° and ∼0.8 Å, respectively). The base pair between the Watson–Crick face of G* and the Hoogsteen face of G is weak (−38.1 or −29.8 kJ/mol, respectively; Supplementary Table S2) and significantly distorted (i.e. deviations up to ∼2 Å and 55° in the base pair width and interplanar angles, respectively, Supplementary Figures S11 and 12).

Several other DNA adducts use their Hoogsteen face to pair with the canonical nucleobases (18,33,34). In the case of POB-G and PHB-G, the strongest interactions with the lesion Hoogsteen face occur with G (−73.8 and −93.9 kJ/mol for POB-G and PHB-G, respectively, Figure 3). Nevertheless, these base pairs deviate from the natural G:C pair in the opening angle by up to ∼25° and base pair width by up to ∼2 Å, and the pairs are nonplanar by up to ∼57° (Figure 3). The hydrogen-bonding interactions between the Hoogsteen face of POB-G or PHB-G, and the Watson–Crick face of C, T or A are weak (–43 to –52 kJ/mol, Supplementary Table S2 and Figures S5–10), and the pairs are structural distorted (i.e. deviations up to ∼3 Å and 20° in the base pair width and opening angles, respectively, Supplementary Figures S5–8).

Overall, the Watson–Crick faces of POB-G and PHB-G form similar hydrogen-bonding interactions with the canonical nucleobases. In particular, stable pairs are characterized between the lesions and C, T and A, with the T pairs being the least distorted. Conversely, the most stable base pairs with the Hoogsteen faces of the lesions occur with G. However, the high degree of distortion predicted for the isolated G*:G mispairs indicates that either the distorted base pairing geometry will not be adopted in the duplex environment or the pair will cause significant structural changes to the overall helical geometry.

### Both lesions preferably extended the bulky moiety into the major groove of DNA, while PHB-G may also adopt an intercalated conformation

**Figure 4.**
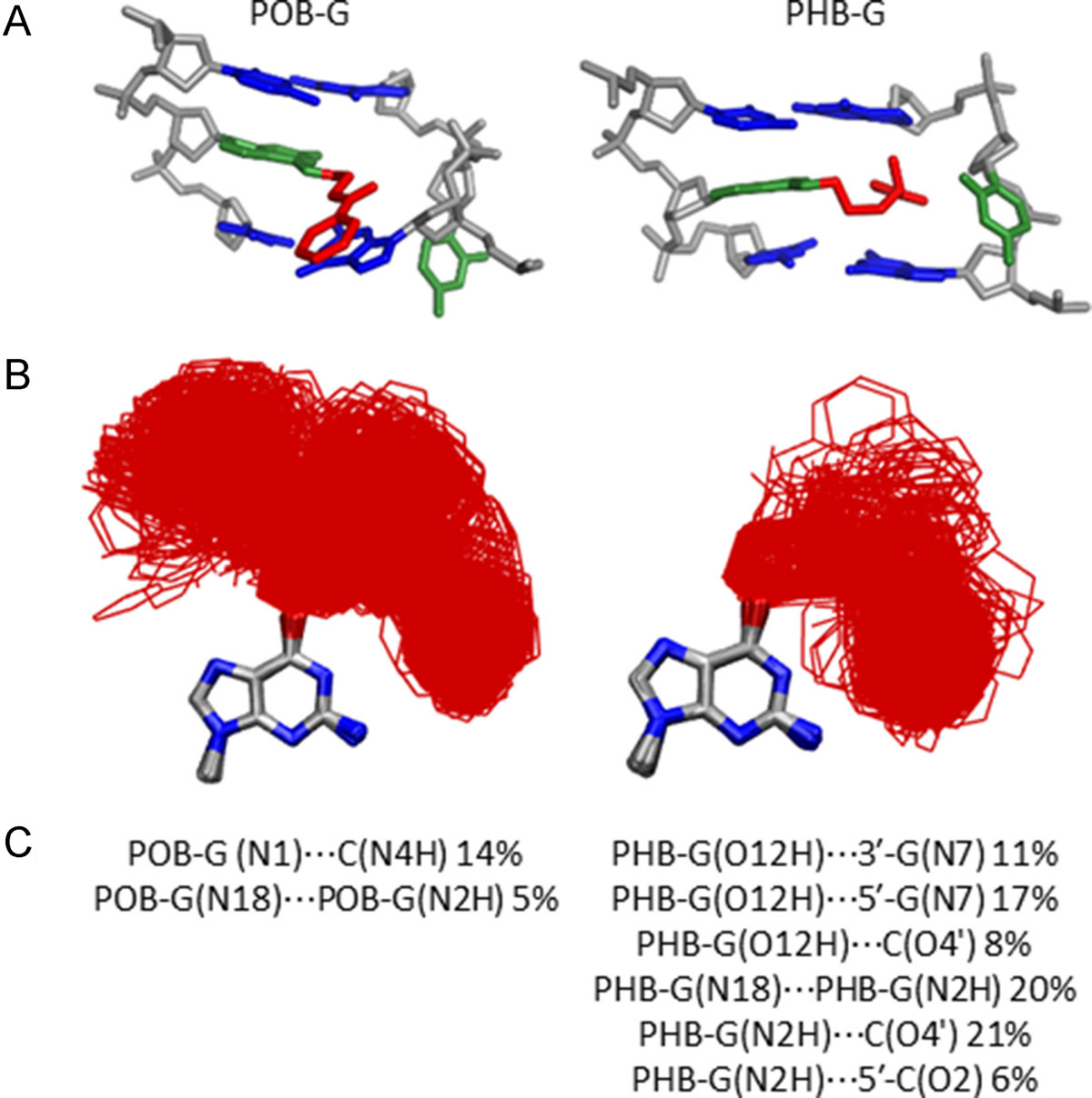
(**A**) MD representative structures obtained for POB-G (left) and PHB-G (right) adducted DNA based on an initial hydrogen-bonded orientation of the lesion with the pairing C in the major groove. (**B**) Overlay of lesion conformations adopted throughout the MD simulations, highlighting the deviation in bulky moiety orientation (red). (**C**) Lesion hydrogen-bonding interactions within DNA.

Since each lesion conformational category identified for the adducted nucleobases will result in a different bulky moiety position in DNA duplexes, MD simulations were initiated from each conformational theme for POB-G or PHB-G paired opposite C to better understand the accessible lesion orientations in DNA and the resulting impacts on DNA structure. Unsurprisingly, the bulky and intrinsically unstable T-shaped nucleobase orientation (Figure 2) does not fit within the confines of the duplex environment. Incorporation of the stacked POB-G or PHB-G conformation into DNA places the bulky moiety between the adducted base pair and the 5′ or 3′ flanking base pair. Regardless of the initial 3′ or 5′ stacked orientation, the bulky moiety consistently adopted an extended orientation during equilibration (Supplementary Figure S13), which suggests the inherently stable stacked conformation of the damaged nucleobases (Figure 2) cannot be accommodated in the duplex. When the lesion hydrogen-bonded conformation is considered, the bulky moiety is situated in the location of the pairing base, forcing the opposing C into an extrahelical position. Although this conformation could be stabilized through stacking interactions between the bulky moiety and flanking base pairs, as well as hydrogen bonding between the bulky moiety and adducted G, POB-G acquires an extended conformation with the bulky moiety in the major groove and stacked with the 3′-G upon simulation, regardless of the initial starting point (Figure 4, left). This suggests that the POB-G hydrogen-bonded conformation is not stable in the duplex. Conversely, PHB-G remains intercalated throughout the simulation (Figure 4, right). In addition to bulky moiety stacking interactions with the flanking pairs and hydrogen-bonding interactions with the adducted G, the intercalated PHB-G position is stabilized through discrete hydrogen-bonding interactions between the bulky moiety hydroxy group and the 5′ or 3′-G (5′, 17% or 3′, 11%; Figure 4, right), which suggests the stability of this conformer is likely sequence dependent.

**Figure 5.**
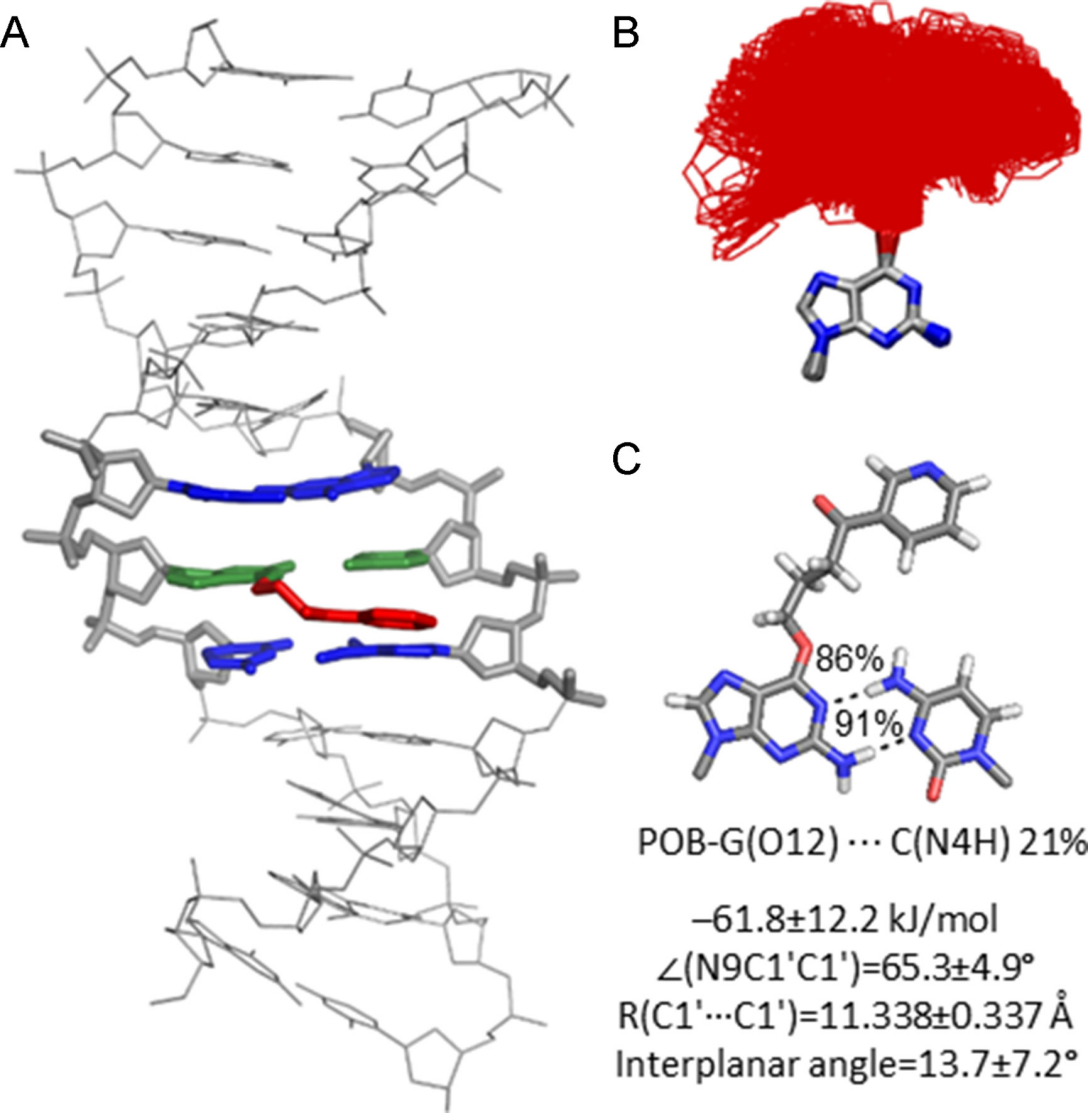
(A) MD representative structure of POB-G-adducted DNA with the lesion in the extended orientation opposite C. (**B**) Overlay of lesion conformations adopted throughout the MD simulations, highlighting the deviation in bulky moiety orientation (red). (**C**) Lesion hydrogen-bonding interactions within DNA.

**Figure 6.**
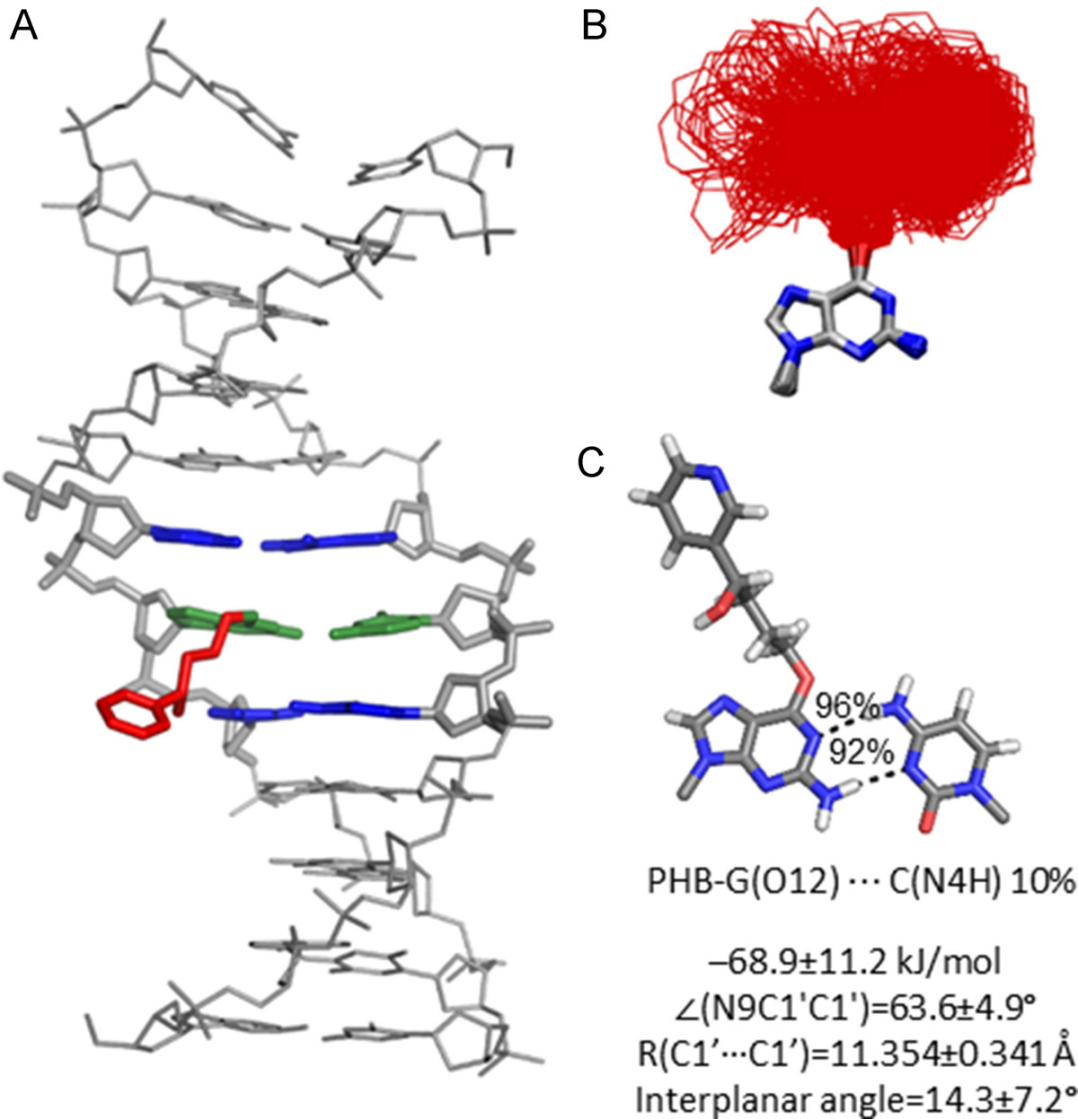
(**A**) MD representative structure of PHB-G-adducted DNA with the lesion in the extended orientation opposite C. (**B**) Overlay of lesion conformations adopted throughout the MD simulations, highlighting the deviation in bulky moiety orientation (red). (**C**) Lesion hydrogen-bonding interactions within DNA.

When the dynamics of the extended POB-G or PHB-G orientation is considered with the bulky moiety in the major groove, many lesion conformations occur in which the bulky moiety does not interact with the surrounding DNA (Figures 5 and 6). This directly correlates with the extended conformation being the inherently most common orientation for the isolated nucleobase (Figure 2). Nevertheless, in some of the sampled adducted DNA conformations, the bulky moiety forms a T-shaped interaction with the Hoogsteen edge of the adducted G or a flanking base, or a hydrogen bond with the pairing C (N4H, ∼20%), which further highlights the inherent lesion flexibility and the predicted similar stability of many nucleobase conformational themes. As predicted by DFT base pair models, both POB-G and PHB-G form a wobble pair with C that contains G*(N1)···C(N4H) and G*(N2H)···C(N3) hydrogen bonds (each occupied for > 86% of the simulation). The pairs are ∼0.5 Å wider and the opening angle is ∼10° larger than for canonical DNA (Figures 5C and 6C; Supplementary Figures S14 and 15). The stability of POB-G:C (−61.8 kJ/mol) is less than PHB-G:C (−68.9 kJ/mol; Supplementary Table S2), as well as canonical G:C. Other global structural feature of the damaged DNA are consistent with those expected for natural DNA throughout the simulation, including the maintenance of the flanking base pairs and base step parameters (Supplementary Figures S14 and 15).

Overall, POB-G adducted DNA adopts one dominant conformational theme that positions the bulky moiety in the major groove and the damaged G hydrogen bonds with the opposing base, which correlates with the single structure of POB-G:C adducted DNA previously reported based on NMR (30). Although we acknowledge limitations in the ability of classical MD simulations to fully sample all lesion site conformations, the current work highlights the diversity of bulky moiety orientations, which is consistent with reported broad NMR peaks (30). In contrast, PHB-G adducted DNA can adopt two conformations: (i) the bulky moiety intercalates into the helix and the pairing base is displaced into the major groove, and (ii) the bulky moiety is in the major groove and the adducted G hydrogen bonds with the opposing base. This difference in the conformational heterogeneity of POB-G and PHB-G damaged DNA provides insight into the potential influence of the bulky moiety composition on mutagenic outcomes. Indeed, it has been proposed that DNA lesions with the ability to stabilize intercalated conformations have a propensity to form deletion mutations (18,33,34). Therefore, our calculations predict that PHB-G may lead to deletion mutations, while the instability of the intercalated conformation for POB-G correlates with the absence of deletion mutations in the reported mutagenic profile for this lesion (21,27). Regardless, both POB-G and PHB-G can maintain hydrogen bonding with an opposing C such that there are only minor distortions to the DNA structure, which reflects the importance of this non-mutagenic pairing in the broader biological context and is consistent with the experimentally-observed non-mutagenic replication of POB-G (21,27).

### Both POB-G and PHB-G form stable interactions with T and A in the DNA duplex

**Figure 7.**
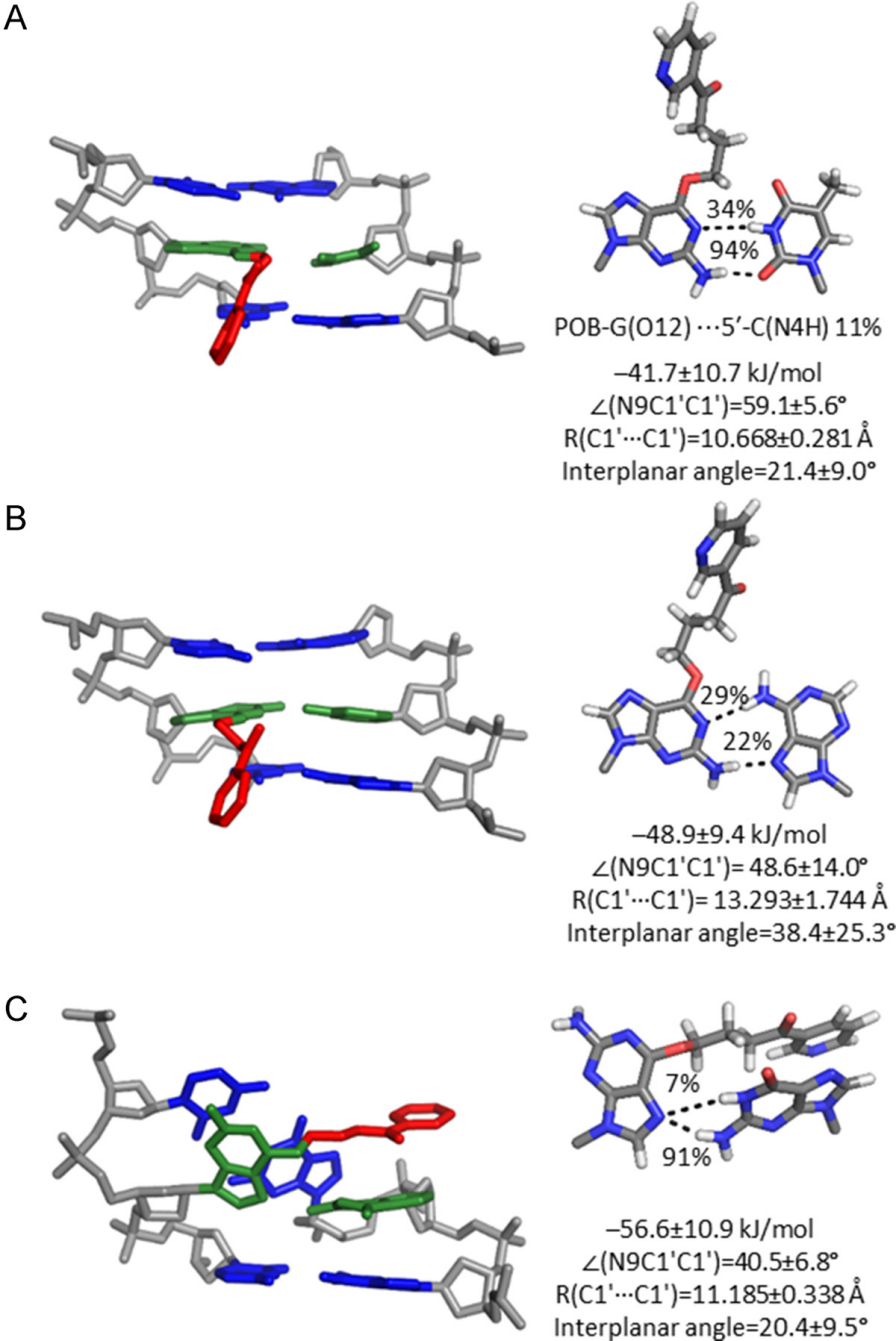
MD representative structures of POB-G-adducted DNA with the bulky moiety in the major groove opposite (**A**) T, (**B**) A or (**C**) G. The trimer containing the lesion pair (green), flanking pairs (blue) and bulky moiety (red) is shown on the left, while the lesion-site hydrogen bonding is shown on the right.

**Figure 8.**
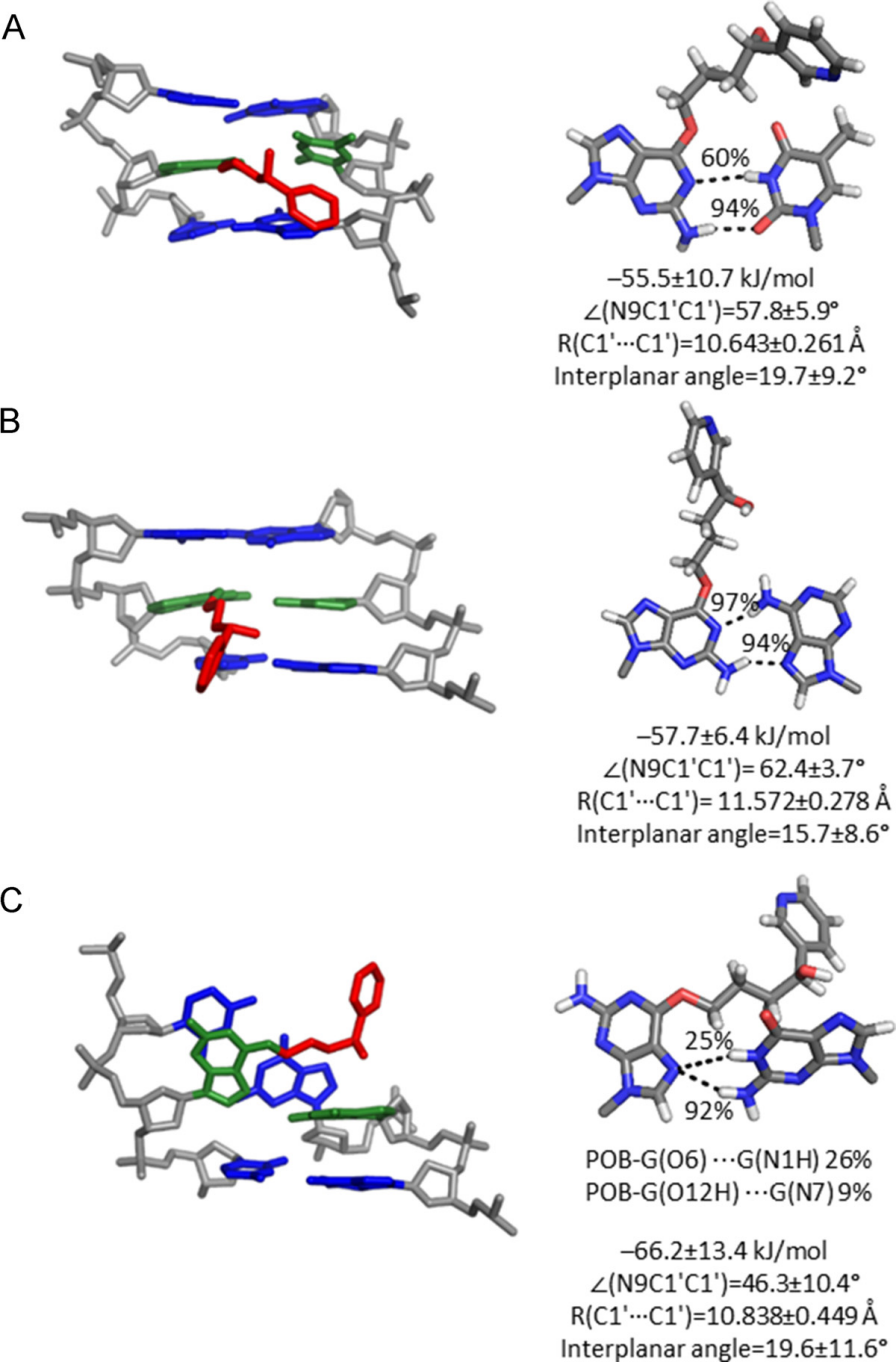
MD representative structures of PHB-G adducted DNA with the bulky moiety in the major groove opposite (**A**) T, (**B**) A or (**C**) G. The trimer containing the lesion pair (green), flanking pairs (blue) and bulky moiety (red) is shown on the left, while the lesion-site hydrogen bonding is shown on the right.

To probe the dynamical structure of post-replication duplexes that correspond to mutagenic events, the POB-G and PHB-G mispairs with T, A and G were modelled in DNA. When POB-G or PHB-G is paired opposite T, a pseudo-Watson–Crick pair is maintained that contains G*(N2H)···T(O2) and G*(N1)···T(N3H) hydrogen bonds (−41.7 or −55.5 kJ/mol for POB-G or PHB-G, respectively; Figures 7A and 8A; Supplementary Table S4). The G*(N1)···T(N3H) interaction is less persistent for POB-G (34%) than PHB-G (60%) due to the orientation of the bulky moiety at the site of attachment (i.e. C10–H is directed toward the POB-G Watson–Crick face to allow for T-shaped interaction between the pyridyl ring with the flanking base pairs, but C10–H is directed toward the PHB-G Hoogsteen face, with transient hydrogen bonding between the bulky moiety hydroxy group and flanking bases). Although the G*:T pairs are 15 to 20 kJ/mol weaker than the G*:C pairs within the helix, the G*:T mispairs cause little deviation in the canonical DNA structure, with a lesion base pair width of ∼10.6 Å and opening of ∼58°, as well as base step and base pair parameters consistent with natural DNA (Supplementary Figures S17 and 18).

When PHB-G is paired opposite A, the lesion forms persistent G*(N2H)···A(N7) and G*(N1)···A(N6H) hydrogen bonds (>90%; Figures 7B and 8B; Supplementary Table S5). In contrast, POB-G forms transient G*(N2H)···A(N7) and G*(N1)···A(N6H) hydrogen bonds (<30%; Figures 7B and 8B and Supplementary Table S5). These pairs are weaker than the G*:C pairs by ∼12 kJ/mol. Furthermore, some deviations occur compared to canonical DNA for DNA containing POB-G:A or PHB-G:A, with the base pair width and opening angle increasing by ∼1 Å and 10°, respectively (Figures 7B and 8B). Nevertheless, no significant deviations occur in the remaining helical parameters (Supplementary Figures S19 and 20).

When *syn*-POB-G or PHB-G is paired opposite G (Supplementary Table S4), a single hydrogen bond occurs between the adducted G(N7) and the pairing G (N2, for >90% of the simulation; Figures 7C and 8C; Supplementary Table S6), which results in pairs that are up to 10 kJ/mol weaker than the G*:C pairs. Although there is little deviation from the canonical base pair width, the lesion mispair is not planar (interplanar angle ∼20°). As a result, the DNA helix is highly distorted with respect to all base step and base pair parameters surrounding the lesion site (Supplementary Figures S21 and 22). For example, the rise and twist between the adducted base pair and the 5′-flanking pair are 2 Å and ∼90° smaller than for an undamaged helix, respectively.

Overall, our simulations on post-replication DNA duplexes suggest that potential base substitution mutations formed upon replication of POB-G and PHB-G due to alternations in the Watson–Crick face of G will afford stable duplexes. Specifically, stable T and A mispairs can exist for both lesions, with the T mispairs causing the least distortion to the DNA duplex. However, the predicted highly distorted helices containing POB-G:G and PHB-G:G suggest that the G mispairs will destabilize the post-replication duplexes and may hinder lesion replication. This proposal is consistent with the observed formation of POB-G:G pairs, but the lack of extension past the lesion site by pol η, ι, κ and ν *in vitro* (21,27).

### Polymerase η insertion complexes indicate that dCTP and dTTP are best aligned for catalytic insertion opposite POB-G and PHB-G, but suggest that PHB-G may display a higher mutagenic potential

**Figure 9.**
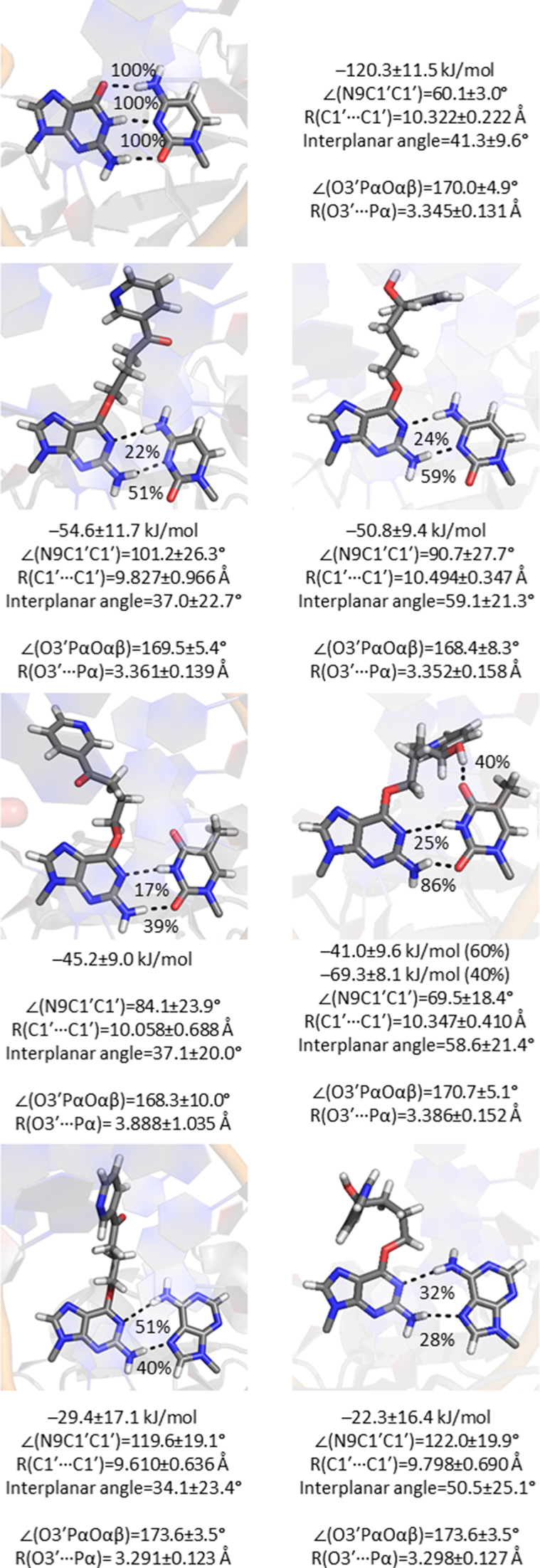
Hydrogen bonding between POB-G (left) or PHB-G (right) and an incoming dCTP, dTTP or dATP in the pol η insertion complex.

To understand the impact of constraints imposed by the pol η active site on the preferred lesion orientations and structures of lesion base pairs, the pol η insertion complexes were considered for dCTP, dTTP or dATP incorporation opposite POB-G and PHB-G (Figure 9). The bulky moiety adopts many distinct extended conformations in the pol η major groove binding pocket (Supplementary Figure S23). Indeed, although there are limitations in the ability of classical MD simulations to sample the entire conformational landscape of flexible lesions, the lesion conformations predicted when bound in the polymerase active site exhibit a high degree of overlap with those for the free damaged nucleobase and the lesion within the DNA duplex. Nevertheless, in contrast to the lesions in unbound DNA, the bulky moiety is exclusively positioned away from the Watson–Crick face of the lesion in the pol η active site.

**Figure 10.**
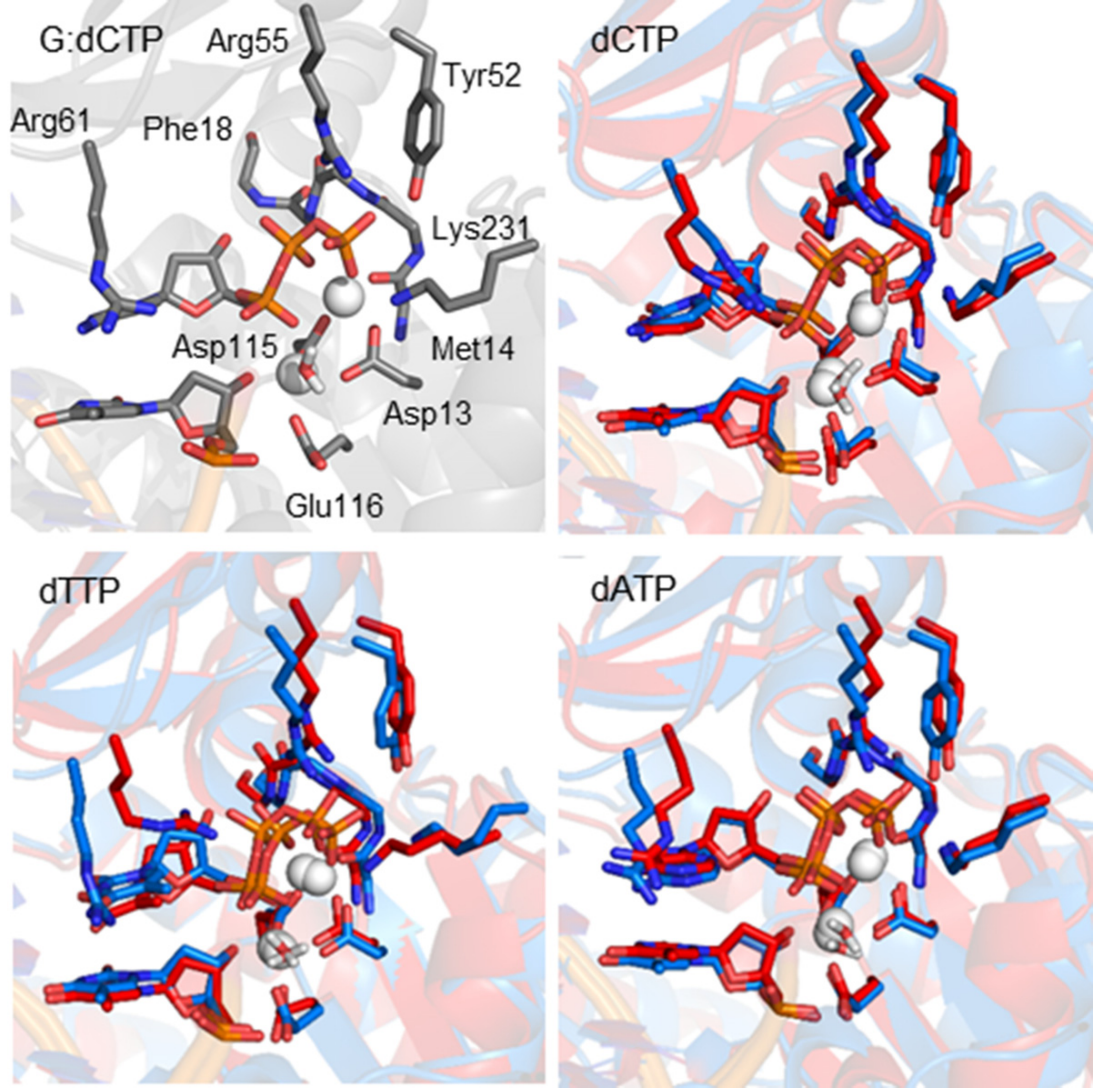
Overlay of the pol η insertion complex for POB-G (blue) and PHB-G (red) replication for dCTP, dTTP or dATP insertion, highlighting the orientation of key active site residues around the dNTP.

Regardless of the lesion conformation, all insertion complexes have key structural features that correctly align the dNTP for the phosphoryl transfer reaction. Specifically, Cys16, Phe17, Phe18, Tyr52, Arg55, Arg61 and Lys231 form hydrogen-bonding interactions with the dNTP (Supplementary Figure S25) (32). Although minor differences exist in the interactions between the polymerase and each dNTP (Figure 10), all dNTPs maintain a network of dNTP-polymerase hydrogen bonds (Tables S7-S9). Additionally, all predicted structures for the pol η insertion complexes associated with POB-G and PHB-G replication maintain octahedral coordination of the binding and catalytic Mg^2+^ ions (Supplementary Table S10), which is known to be essential for catalysis (32). Nevertheless, the coordination of Oα2 to the binding Mg^2+^ ion adopts a wide spread of distances (Supplementary Figure S24). Finally, for a polymerase insertion complex to be catalytically active, the reaction coordinates should be aligned, which includes: (i) the distance between the 3′-primer end(O3′) and the dNTP(Pα) approaching the van der Waals radii for O and P (3.5 Å), and (ii) the in-line attack angle [∠(3′-primer end(O3′)–dNTP(Pα)–dNTP(Oαβ))] approaching 180° (35–37). All POB-G and PHB-G replication complexes maintain this reaction coordinate orientation, with a distance < ∼3.3 Å and angle > ∼170° (Figure 9).

In addition to the contacts between pol η and the dNTP triphosphate tail, the position of the dNTP in the active site is stabilized by hydrogen-bonding interactions with the lesion in the template strand. Indeed, hydrogen bonding between the template base and incoming dNTP has been proposed to play an important role in dNTP selection by TLS polymerases (16,38,39). Consistent with DNA duplex models (Figures 5–8), POB-G and PHB-G form a wobble pair with dCTP that contains G*(N1)···dCTP(N4H) (>20%) and G*(N2H)···dCTP(N3) (>50%) interactions, and has an overall interaction strength of ∼−50 kJ/mol (Figure 9). The POB-G and PHB-G pairs with dCTP have opening angles significantly wider than G:dCTP in the pol η active site (up to ∼35°), but have the same interplanar angle as G:dCTP. While the PHB-G:dCTP pair has the same width as G:dCTP, the POB-G:dCTP pair is ∼0.5 Å narrower.

The lesions also form two hydrogen bonds with dATP, namely the G*(N1)···dATP(N6H) (<50%) and G*(N2H)···dATP(N7) (<40%) hydrogen bonds. Nevertheless, dATP incorporation results in a weak base-pair strength (∼−25 kJ/mol; Figure 9). Furthermore, the dATP pairs contain wide opening angles (∼120°) and a narrow width (∼9.7 Å), but an interplanar angle consistent with canonical DNA replication.

As seen for unbound DNA, a persistent pseudo-Watson–Crick pair occurs between dTTP and POB-G or PHB-G that contains an intermittent POB-G(N1)···dTTP(N3H) interaction (17 or 25%, respectively) and a POB-G(N2H)·dTTP(O2) hydrogen bond (39 or 86%, respectively; Figure 9). When only these two interactions are formed between the adducted nucleobase and the incoming dTTP, both pairs have an interaction strength of ∼−45 kJ/mol. However, in contrast to unbound DNA and dTTP insertion opposite POB-G within the pol η active site, the PHB-G:dTTP pair can also contain a hydrogen bond between the bulky moiety hydroxy group and O4 of the pairing dTTP (40%). When this bulky moiety–dTTP contact is present, the interaction strength increases to ∼−70 kJ/mol. Irrespective of the involvement of the bulky moiety in dTTP binding, both the POB-G and PHB-G pairs maintain a structure consistent with canonical DNA.

Overall, regardless of the catalytically conducive alignment of the pol η active site, the lack of strong, persistent and undistorted hydrogen bonding between A and the lesion suggests that dATP is unlikely to be selectively incorporated opposite POB-G or PHB-G by pol η. On the other hand, dCTP insertion results in a more stable and a less distorted hydrogen-bonded pair with both lesions, as well as a correct active site configuration. Finally, persistent hydrogen bonding occurs between both lesions and an incoming dTTP, which leads to base pair structural parameters more consistent with natural DNA than dCTP. Therefore, our calculations predict that pol η replication of these two key tobacco induced DNA lesions will lead to preferential dNTP insertion according to dTTP > dCTP >> dATP. This is consistent with experimental studies on POB-G, which report a high frequency of G → A mutations and non-mutagenic replication (21,26,27), and highlights the predictive power of computer modelling for understanding the mutagenicity of DNA damage. In the absence of experimental data, the same computational approach reveals that the PHB-G:dTTP pair can be significantly more stable in the pol η active site than POB-G:dTTP, and therefore indicates that PHB-G may be more mutagenic. This effect is caused by a difference in the bulky moiety composition, and therefore this finding complements previous literature that emphasizes the influence of adduct structure on the mutational outcomes of DNA lesions (14–25).

## CONCLUSION

The current study uses a multiscale computational approach to provide structural data that rationalizes and predicts the mutagenicity of two tobacco-derived carcinogenic DNA lesions, namely POB-G and PHB-G. Quantum mechanical calculations reveal that both POB-G and PHB-G possess a high degree of inherent conformational flexibility, which may affect the biological processing of the lesions and emphasizes the importance of dynamical structural data for other flexible DNA lesions. Nevertheless, regardless of the conformation adopted, both lesions form stable and minimally distorted nucleobase pairs with C, T and A, but highly distorted pairs with G. Indeed, MD simulations predict that the G mispairs significantly alter the DNA duplex, suggesting that dGTP incorporation will not afford stable post-replication duplexes. This proposal is consistent with the previously reported formation of, but lack of extension past, POB-G:G pairs by TLS polymerases *in vitro* (21,27). In contrast, stable minimally distorted duplexes occur when POB-G or PHB-G are paired opposite C, T and A. However, dATP weakly interacts with either lesion when bound within the polymerase, and therefore is unlikely to be selectively incorporated opposite POB-G or PHB-G, despite a catalytically conducive alignment of the pol η active site. This finding agrees with the observed low frequency of G → T mutations upon replication of POB-G *in vivo* (2%) (26). When dCTP and dTTP are placed opposite POB-G or PHB-G, the pol η active site is aligned for catalysis and the dNTP forms stable interactions with both lesions. However, the G*:dTTP pairs are less distorted, suggesting dTTP will be inserted more often than dCTP. These findings are consistent with experimental studies that report an abundance of G → A mutations, as well as non-mutagenic replication, for POB-G (21,26,27), which validates our approach. Nevertheless, differences in the bulky moiety hydrogen-bonding patterns enhance the stability of the PHB-G:dTTP pair, suggesting that dTTP will be more often misinserted opposite PHB-G. Furthermore, the mutagenicity of PHB-G is likely increased by the stabilization of a conformation that intercalates the bulky moiety into the DNA helix, which has been associated with deletion mutations for other adducts. This contrasts the instability of an analogous conformer for POB-G, which coincides with the experimental mutagenicity spectrum. Nevertheless, future experimental work that probes the mutational profile of PHB-G is required to confirm the relative importance of deletion mutations for different tobacco-derived lesions. Overall, this study provides key structural insight into the reported mutagenicity of POB-G, uncovers the first clues about the mutagenicity of PHB-G, and adds to a growing body of literature that highlights the impact of small chemical changes to the bulky moiety on lesion mutagenicity.

## Supplementary Material

Supplementary Data

## References

[1] Herrmann S.S., Duedahl-Olesen L., Christensen T., Olesen P.T., Granby K. Dietary exposure to volatile and non-volatile N-nitrosamines from processed meat products in Denmark. Food Chem. Toxicol. 2015; 80:137–143. 25792266 10.1016/j.fct.2015.03.008

[2] Al-Kaseem M., Al-Assaf Z., Karabeet F. Determination of seven volatile N-nitrosamines in fast food. Pharmacol. Pharm. 2014; 5:195–203.

[3] Proksch E. Review toxicological evaluation of nitrosamines in condoms. Int. J. Hyg. Environ. Health. 2001; 204:103–110. 11759152 10.1078/1438-4639-00087

[4] Lijinsky W. The significance of N‐nitroso compounds as environmental carcinogens. Environ. Carcinogenesis Rev. 1986; 4:1–45.

[5] Peterson L.A. Context matters: contribution of specific DNA adducts to the genotoxic properties of the tobacco-specific nitrosamine NNK. Chem. Res. Toxicol. 2016; 30:420–433. 28092943 10.1021/acs.chemrestox.6b00386 PMC5473167

[6] St.Helen G., Benowitz N.L., Dains K.M., Havel C., Peng M., Jacob P. Nicotine and carcinogen exposure after water pipe smoking in hookah bars. Cancer Epidemiol. Biomarkers Prev. 2014; 23:1055–1066. 24836469 10.1158/1055-9965.EPI-13-0939 PMC4047652

[7] Kim H.-J., Shin H.-S. Determination of tobacco-specific nitrosamines in replacement liquids of electronic cigarettes by liquid chromatography–tandem mass spectrometry. J. Chromatogr. A. 2013; 1291:48–55. 23602640 10.1016/j.chroma.2013.03.035

[8] Hecht S.S. Biochemistry, biology, and carcinogenicity of tobacco-specific N-nitrosamines. Chem. Res. Toxicol. 1998; 11:559–603. 9625726 10.1021/tx980005y

[9] Hecht S.S. Progress and challenges in selected areas of tobacco carcinogenesis. Chem. Res. Toxicol. 2008; 21:160–171. 18052103 10.1021/tx7002068 PMC2556958

[10] Alavanja M., Baron J., Brownson R.C., Buffler P.A., DeMarini D.M., Djordjevic M.V., Doll R., Fontham E.T., Gao Y.T., Gray N. Tobacco smoke and involuntary smoking. IARC monographs on the evaluation of carcinogenic risks to humans. 2004; 83:1–1413. 15285078 PMC4781536

[11] Hecht S.S. It is time to regulate carcinogenic tobacco-specific nitrosamines in cigarette tobacco. Cancer Prev. Res. 2014; 7:639–647. 10.1158/1940-6207.CAPR-14-0095 PMC4135519 24806664

[12] Czoli C.D., Hammond D. Trends over time in tobacco-specific nitrosamines (TSNAs) in whole tobacco and smoke emissions from cigarettes sold in Canada. Nicotine Tob. Res. 2018; 20:649–653. 28595283 10.1093/ntr/ntx103 PMC5892861

[13] Siegel R.L., Miller K.D., Jemal A. Cancer statistics, 2015. CA Cancer J Clin. 2015; 65:5–29. 25559415 10.3322/caac.21254

[14] Patra A., Nagy L.D., Zhang Q.Q., Su Y., Muller L., Guengerich F.P., Egli M. Kinetics, structure, and mechanism of 8-oxo-7,8-dihydro-2′-deoxyguanosine bypass by human DNA polymerase η. J. Biol. Chem. 2014; 289:16867–16882. 24759104 10.1074/jbc.M114.551820 PMC4059130

[15] Gahlon H.L., Schweizer W.B., Sturla S.J. Tolerance of base pair size and shape in postlesion DNA synthesis. J. Am. Chem. Soc. 2013; 135:6384–6387. 23560524 10.1021/ja311434s

[16] Gahlon H.L., Boby M.L., Sturla S.J. O6-alkylguanine postlesion DNA synthesis is correct with the right complement of hydrogen bonding. ACS Chem. Biol. 2014; 9:2807–2814. 25259614 10.1021/cb500415q

[17] Kirouac K.N., Basu A.K., Ling H. Structural mechanism of replication stalling on a bulky amino-polycyclic aromatic hydrocarbon DNA adduct by a Y-family DNA polymerase. J. Mol. Biol. 2013; 425:4167–4176. 23876706 10.1016/j.jmb.2013.07.020 PMC3894629

[18] Stover J.S., Chowdhury G., Zang H., Guengerich F.P., Rizzo C.J. Translesion synthesis past the C8- and N2-deoxyguanosine adducts of the dietary mutagen 2-amino-3-methylimidazo[4,5-f]quinoline in the *Nar*I recognition sequence by prokaryotic DNA polymerases. Chem. Res. Toxicol. 2006; 19:1506–1517. 17112239 10.1021/tx0601455 PMC3150502

[19] Zhang H., Eoff R.L., Kozekov I.D., Rizzo C.J., Egli M., Guengerich F.P. Versatility of Y-family *Sulfolobus solfataricus* DNA polymerase Dpo4 in translesion synthesis past bulky N2-alkylguanine adducts. J. Biol. Chem. 2009; 284:3563–3576. 19059910 10.1074/jbc.M807778200 PMC2636699

[20] Eoff R.L., Angel K.C., Egli M., Guengerich F.P. Molecular basis of selectivity of nucleoside triphosphate incorporation opposite O6-benzylguanine by *Sulfolobus solfataricus* DNA polymerase Dpo4: Steady-state and pre-steady-state kinetics and X-ray crystallography of correct and incorrect pairing. J. Biol. Chem. 2007; 282:13573–13584. 17337730 10.1074/jbc.M700656200

[21] Choi J.-Y., Chowdhury G., Zang H., Angel K.C., Vu C.C., Peterson L.A., Guengerich F.P. Translesion synthesis across O6-alkylguanine DNA adducts by recombinant human DNA polymerases. J. Biol. Chem. 2006; 281:38244–38256. 17050527 10.1074/jbc.M608369200

[22] Sherrer S.M., Brown J.A., Pack L.R., Jasti V.P., Fowler J.D., Basu A.K., Suo Z. Mechanistic studies of the bypass of a bulky single-base lesion catalyzed by a Y-family DNA polymerase. J. Biol. Chem. 2009; 284:6379–6388. 19124465 10.1074/jbc.M808161200 PMC2649090

[23] Gadkari V.V., Tokarsky E.J., Malik C.K., Basu A.K., Suo Z. Mechanistic investigation of the bypass of a bulky aromatic DNA adduct catalyzed by a Y-family DNA polymerase. DNA Rep. 2014; 21:65–77. 10.1016/j.dnarep.2014.06.003 PMC4133103 25048879

[24] Xu P., Oum L., Lee Y.-C., Geacintov N.E., Broyde S. Visualizing sequence-governed nucleotide selectivities and mutagenic consequences through a replicative cycle: Processing of a bulky carcinogen N2-dG lesion in a Y-family DNA polymerase. Biochemistry. 2009; 48:4677–4690. 19364137 10.1021/bi802363f PMC2929011

[25] Wyss L.A., Nilforoushan A., Eichenseher F., Suter U., Blatter N., Marx A., Sturla S.J. Specific incorporation of an artificial nucleotide opposite a mutagenic DNA adduct by a DNA polymerase. J. Am. Chem. Soc. 2015; 137:30–33. 25490521 10.1021/ja5100542

[26] Pauly G.T., Peterson L.A., Moschel R.C. Mutagenesis by O6-[4-oxo-4-(3-pyridyl)butyl]guanine in *Escherichia coli* and human cells. Chem. Res. Toxicol. 2002; 15:165–169. 11849042 10.1021/tx0101245

[27] Gowda A.S.P., Spratt T.E. DNA polymerase ν rapidly bypasses O6-methyl-dG but not O6-[4-(3-pyridyl)-4-oxobutyl-dG and O2-alkyl-dTs. Chem. Res. Toxicol. 2016; 29:1894–1900. 27741574 10.1021/acs.chemrestox.6b00318 PMC5673091

[28] Carmella S., Ye M., Upadhyaya P., Hecht S.S. Stereochemistry of metabolites of a tobacco-specific lung carcinogen in smokers' Urine. Cancer Res. 1999; 59:3602–3605. 10446969

[29] Park S., Seetharaman M., Ogdie A., Ferguson D., Tretyakova N. 3′‐exonuclease resistance of DNA oligodeoxynucleotides containing O6‐[4‐oxo‐4‐(3‐pyridyl) butyl] guanine. Nucleic Acids Res. 2003; 31:1984–1994. 12655016 10.1093/nar/gkg299 PMC152814

[30] Peterson L.A., Vu C., Hingerty B.E., Broyde S., Cosman M. Solution structure of an O6-[4-oxo-4-(3-pyridyl)butyl]guanine adduct in an 11mer DNA duplex: Evidence for formation of a base triplex. Biochemistry. 2003; 42:13134–13144. 14609323 10.1021/bi035217v

[31] Wilson K.A., Szemethy K.G., Wetmore S.D. Conformational flexibility and base-pairing tendency of the tobacco carcinogen O6-[4-oxo-4-(3-pyridyl)butyl]guanine. Biophys. Chem. 2017; 228:25–37. 28654813 10.1016/j.bpc.2017.06.001

[32] Nakamura T., Zhao Y., Yamagata Y., Hua Y.-J., Yang W. Watching DNA polymerase η make a phosphodiester bond. Nature. 2012; 487:196–201. 22785315 10.1038/nature11181 PMC3397672

[33] Norman D., Abuaf P., Hingerty B.E., Live D., Grunberger D., Broyde S., Patel D.J. NMR and computational characterization of the N-(deoxyguanosin-8-yl) aminofluorene adduct [(AF)-G] opposite adenosine in DNA:(AF)-G [*syn*]:A [*anti*] pair formation and its pH dependence. Biochemistry. 1989; 28:7462–7476. 2819081 10.1021/bi00444a046

[34] Gu Z., Gorin A., Hingerty B.E., Broyde S., Patel D.J. Solution structures of aminofluorene [AF]-stacked conformers of the *syn* [AF]−C8-dG adduct positioned opposite dC or dA at a template-primer junction. Biochemistry. 1999; 38:10855–10870. 10451382 10.1021/bi991266p

[35] Brautigam C.A., Steitz T.A. Structural and functional insights provided by crystal structures of DNA polymerases and their substrate complexes. Curr. Opin. Struct. Biol. 1998; 8:54–63. 9519297 10.1016/s0959-440x(98)80010-9

[36] Wang Y., Schlick T. Quantum mechanics/molecular mechanics investigation of the chemical reaction in Dpo4 reveals water-dependent pathways and requirements for active site reorganization. J. Am. Chem. Soc. 2008; 130:13240–13250. 18785738 10.1021/ja802215c PMC3195406

[37] Wang L., Yu X., Hu P., Broyde S., Zhang Y. A water-mediated and substrate-assisted catalytic mechanism for *Sulfolobus solfataricus* DNA polymerase IV. J. Am. Chem. Soc. 2007; 129:4731–4737. 17375926 10.1021/ja068821c PMC2519035

[38] Washington M.T., Helquist S.A., Kool E.T., Prakash L., Prakash S. Requirement of Watson-Crick hydrogen bonding for DNA synthesis by yeast DNA polymerase η. Mol. Cell. Biol. 2003; 23:5107–5112. 12832493 10.1128/MCB.23.14.5107-5112.2003 PMC162216

[39] Wolfle W.T., Washington M.T., Kool E.T., Spratt T.E., Helquist S.A., Prakash L., Prakash S. Evidence for a Watson-Crick hydrogen bonding requirement in DNA synthesis by human DNA polymerase κ. Mol. Cell. Biol. 2005; 25:7137–7143. 16055723 10.1128/MCB.25.16.7137-7143.2005 PMC1190260

