# Supplementary material for: Computational insights into the mutagenicity of two tobacco-derived carcinogenic DNA lesions
Source: Nucleic Acids Res. 2018 Nov 8;46(22):11858–68. doi: 10.1093/nar/gky1071 (PMC6294509; doi:10.1093/nar/gky1071)
Supplement: Supplementary Data [file gky1071_supplemental_files.pdf]

Computational Insights into the Mutagenicity of Two Tobacco Derived Carcinogenic DNA Lesions

Katie A. Wilson, Josh L. Garden, Natasha T. Wetmore and Stacey D. Wetmore*

Department of Chemistry and Biochemistry, University of Lethbridge, 4401 University Drive West, Lethbridge, Alberta T1K 3M4, Canada

Supporting Information

Contents

Full Methods...4

Table S1. Heavy atom rmsd (Å) with respect to the representative structure for Trial 1. 8

Table S2. B3LYP-D3(BJ)/6-311+G(2df,2p)//M06-2X/6-31G(d) interaction energies (kJ/mol) for base pairs between the Watson-Crick or Hoogsteen hydrogen-bonding face of G, or various POB-G or PHB-G conformers (G*), and the canonical nucleobases.[a] 9

Table S3. Occupancies (%), average heavy atom distances (Å), and average angles (deg.) for hydrogen bonds in the adducted, and 3′ and 5′-flanking base pairs across the entire 500ns MD simulation trajectory for POB-G or PHB-G adducted DNA with the lesion paired opposite C. 10

Table S4. Occupancies (%), average heavy atom distances (Å), and average angles (deg.) for hydrogen bonds in the adducted, and 3′ and 5′-flanking base pairs across the entire 500 ns MD simulation trajectory for POB-G or PHB-G adducted DNA with the lesion paired opposite T. 11

Table S5. Occupancies (%), average heavy atom distances (Å), and average angles (deg.) for hydrogen bonds in the adducted, and 3′ and 5′-flanking base pairs across the entire 500 ns MD simulation trajectory for POB-G or PHB-G adducted DNA with the lesion paired opposite A. 12

Table S6. Occupancies (%), average heavy atom distances (Å), and average angles (deg.) for hydrogen bonds in the adducted, and 3′ and 5′-flanking base pairs across the entire 500 ns MD simulation trajectory for POB-G or PHB-G adducted DNA with the lesion paired opposite G. 13

Table S10. Coordination of the active site Mg^{2+} ions across the entire MD simulation trajectory for the polymerase η insertion complex corresponding to various lesion replication outcomes.[a] 17

Table S11. Atom types and charges for POB-G and PHB-G. 18

Table S12. Occupancies (%), average heavy atom distances (Å), and average angles (deg.) for hydrogen bonds in the adducted, and 3′ and 5′-flanking base pairs across the entire 300 ns MD simulation trajectory for POB-G or PHB-G adducted DNA with the lesion paired opposite C. 19

Table S13. Occupancies (%), average heavy atom distances (Å), and average angles (deg.) for hydrogen bonds in the adducted, and 3′ and 5′-flanking base pairs across the entire 300 ns MD simulation trajectory for POB-G or PHB-G adducted DNA with the lesion paired opposite T. 20

Table S14. Occupancies (%), average heavy atom distances (Å), and average angles (deg.) for hydrogen bonds in the adducted, and 3′ and 5′-flanking base pairs across the entire 300 ns MD simulation trajectory for POB-G or PHB-G adducted DNA with the lesion paired opposite A. 21

Table S15. Occupancies (%), average heavy atom distances (Å), and average angles (deg.) for hydrogen bonds in the adducted, and 3′ and 5′-flanking base pairs across the entire 300 ns MD simulation trajectory for POB-G or PHB-G adducted DNA with the lesion paired opposite G. 22

Figure S1. Distribution of the a) POB-G and b) PHB-G nucleobase conformations resulting from the DFT conformational search. 26

Figure S2. Distribution of the B3LYP-D3(BJ)/6-311+G(2df,2p) relative energies (kJ/mol) of the a) POB-G and b) PHB-G nucleobase conformations for each structural category. 27

Figure S3. For each structural category B3LYP-D3(BJ)/6-311+G(2df,2p)//B3LYP-D3(BJ)/6-31G(d) a) *anti* and b) *syn* structures of the POB-G nucleoside, as well as the relative energies (kJ/mol). 28

Figure S4. For each structural category B3LYP-D3(BJ)/6-311+G(2df,2p)//B3LYP-D3(BJ)/6-31G(d) a) *anti* and b) *syn* structures of the PHB-G nucleoside, as well as the relative energies (kJ/mol). 29

Figure S5. B3LYP-D3(BJ)/6-311+G(2df,2p)//M06-2X/6-31G(d) structures (distances in Å and angles in deg.) and binding energy (kJ/mol) for dimers between the Watson-Crick (left) or Hoogsteen (right) face of various POB-G conformations and C. 30

Figure S7. B3LYP-D3(BJ)/6-311+G(2df,2p)//M06-2X/6-31G(d) structures (distances in Å and angles in deg.) and binding energy (kJ/mol) for dimers between the Watson-Crick (left) or Hoogsteen (right) face of various POB-G conformations and T. 32

Figure S9. B3LYP-D3(BJ)/6-311+G(2df,2p)//M06-2X/6-31G(d) structures (distances in Å and angles in deg.) and binding energy (kJ/mol) for dimers between the Watson-Crick (left) or Hoogsteen (right) face of various POB-G conformations and A. 34

Figure S12. B3LYP-D3(BJ)/6-311+G(2df,2p)//M06-2X/6-31G(d) structures (distances in Å and angles in deg.) and binding energy (kJ/mol) for dimers between the Watson-Crick (left) or Hoogsteen (right) face of various PHB-G conformations and G. 37

Figure S13. a) MD representative structures obtained for POB-G (left) and PHB-G (right) adducted DNA based on an initial stacked lesion conformation opposite C. b) Overlay (based on G ring atoms) of lesion conformations adopted throughout the MD simulation, highlighting the deviation in bulky moiety orientation (red). 38

Figure S14. Average base step parameters from MD simulations on a) POB-G or b) PHB-G adducted DNA with the lesion in the extended conformation paired opposite C. 39

Figure S15. Average base pair parameters for a) POB-G or b) PHB-G adducted DNA with the lesion in the extended conformation paired opposite C. 40

Figure S16. Average base step parameters for a) POB-G or b) PHB-G adducted DNA with the lesion in the extended conformation paired opposite C. 40

Figure S17. Average base step parameters for a) POB-G or b) PHB-G adducted DNA with the lesion in the extended conformation paired opposite T. 40

Figure S18. Average base pair parameters for a) POB-G or b) PHB-G adducted DNA with the lesion in the extended conformation paired opposite T. 43

Figure S20. Average base pair parameters for a) POB-G or b) PHB-G adducted DNA with the lesion in the extended conformation paired opposite A. 45

Figure S21. Average base step parameters for a) POB-G or b) PHB-G adducted DNA with the lesion in the extended conformation paired opposite G. 46

Figure S22. Average base pair parameters for a) POB-G or b) PHB-G adducted DNA with the lesion in the extended conformation paired opposite G. 47

Figure S23. Overlay (based on G ring atoms) of lesion conformations adopted throughout the MD simulations on polymerase η complexes corresponding to dCTP (top), dTTP (middle), or dATP (bottom) insertion opposite POB-G (left) or PHB-G (right), highlighting the deviation in bulky moiety orientation (red)........ 48

Figure S24. Distance between the binding Mg^{2+} ion and coordinating Oα during MD simulations on the insertion of a dNTP opposite POB-G or PHB-G by polymerase η. Full coordination was considered to occur if the distance was < 2.5 Å for > 95% of the simulation. 49

Figure S25. Hydrogen-bonding interactions with the dNTP in the polymerase η active site observed in the crystal structure corresponding to dATP incorporation opposite T (PDB ID: 4ECS). 50

Full Methods

DFT Calculations

Nucleobase model: Due to the anticipated high degree of flexibility within POB-G and PHB-G, the inherent conformational preference about the nucleobase–carcinogen linker and within the bulky moiety was initially examined using a nucleobase model. Specifically, a usage directed conformational search about the α′, β′,γ′, δ′, ϵ′, ρ′, and [illegible]′ dihedral angles within the bulky moiety (Figure 1) was completed for each lesion as implemented in Hyperchem (1), with a maximum of 100,000 iterations or 1000 optimizations. Unique structures were defined based on an energy difference of > 0.2 kJ/mol and a heavy atom root-mean-square deviation (rmsd) of 0.25 Å. During the search, each adduct was modeled with AMBER99 charges and atom types (Table S13) and structures within 40 kJ/mol of the most stable structure were saved. All orientations of POB-G and PHB-G isolated from the conformational search were subsequently optimized using B3LYP-D3(BJ)/6-31G(d) and the relative energies were determined using B3LYP-D3(BJ)/6-311+G(2df,2p). Each unique structure (based on an energy difference of > 0.2 kJ/mol and difference of > 0.2° in the key bulky moiety dihedral angles) was considered for further analysis, including the classification of the lesion orientation based on the position of the bulky moiety relative to the adducted G. Specifically, all structures were visually inspected and classified based on discrete interactions between the bulky moiety and adducted G as stacked, hydrogen bonded, T-shaped or extended (no direct interaction between the bulky moiety and adducted G).

Nucleoside model: To assess the relative stability of the *anti* (χ ≈ 220°) and *syn* (χ ≈ 60°) conformations about the glycosidic bond in the nucleoside adducts, 2′-deoxyribose was added to the most stable orientation for each conformational category of POB-G and PHB-G identified using the nucleobase models. In the nucleoside adducts, the 2′-*endo* sugar pucker was used, and the O3′ hydroxy groups was oriented and the O5′ hydroxy groups was fixed to reflect the geometry of a non-terminal nucleoside (i.e., ∠(HC3′O3′H) ≈ −60° and ∠(C4′C5′O5′H) = 180°). Each nucleoside model was optimized in the *anti* and *syn* orientations using B3LYP-D3(BJ)/6-31G(d) and the relative energies were determined using B3LYP-D3(BJ)/6-311+G(2df,2p).

Hydrogen-Bonding Interactions: Hydrogen-bonded pairs between the Watson-Crick or Hoogsteen face of the most stable orientation of POB-G or PHB-G from each conformational category, and each of the four canonical DNA bases were optimized using M06-2X/6-31G(d). This method was used since previous work has shown that B3LYP-D3(BJ)/6-31G(d) optimizations of G mispairs give rise to a single, small imaginary frequency, while stable minima with nearly identical structures were identified when the same hydrogen-bonded complexes were optimized with M06-2X/6-31G(d) (2). Furthermore, no significant structural or energetic differences were observed between the most stable nucleobase and nucleoside conformations optimized with B3LYP-D3(BJ)/6-31G(d) and M06-2X/6-31G(d) (2). In the computational model, 2′-deoxyribose was replaced with a methyl group since the nucleoside model indicates that the sugar does not affect the conformation of the lesion. Furthermore, the methyl groups allow the base pair width (C1′–C1′ distance) and opening angle (∠(N9C1′C1′)) within the adducted base pairs to be assessed. The relative energies were determined using B3LYP-D3(BJ)/6-311+G(2df,2p).

All DFT calculations were performed using Gaussian 09 (revision D.01) (3).

Molecular Dynamics Simulations

Parameters: All natural amino acids, nucleotides, and the solvent were modeled with AMBER ff14SB parameters (4). Parameters for POB-G and PHB-G were assigned according to the GAFF (5) and AMBER ff14SB force fields using ANTECHAMBER 1.4 (6), and partial charges for the lesions were developed using

RESP charge fitting from a HF/6-31G(d) calculation by the R.E.D.v.III.4 program (7,8) (Table S11). The parameters for the dNTPs were adapted from the literature (9-11), Mg^{2+} was modeled with the parameters from Allner *et al.* (12), and Na^{+} and Cl^{-} were modeled with the monovalent ion parameters from Joung & Cheatham (13).

DNA model: MD simulations were performed on the 5′–CTCGGCG*CCATC 12-mer DNA helix. The initial choices of the key dihedral angles in the bulky moiety (α′, β′, γ′, δ′, ϵ′, ρ′, ζ′, and χ; Figure 1) and the lesion site base-pairing geometry were directed by the DFT calculations. Specifically, MD simulations were initially conducted with the lesion paired opposite C in a wobble base-pairing arrangement or with the lesion intercalated and the pairing C in the major groove. POB-G or PHB-G were positioned at G* in an orientation that represents each of the conformational categories identified in the nucleobase conformational search. Since the bulky moiety of the lesion resides in the major groove regardless of the orientation about the glycosidic bond, the conclusions about the preferred lesion orientation can be applied to POB-G and PHB-G regardless of the glycosidic orientation. Therefore, MD simulations were subsequently performed with the lesion mispaired opposite T, A, or G, with the bulky moiety initiated in the preferred orientation identified from the simulations on DNA containing the lesions paired opposite C.

Initial DNA helices were prepared for simulation by neutralizing the system with 24 Na^{+} ions, and solvating the DNA in a TIP3P octahedral water box such that the DNA was at least 8.0 Å from the box edge. The systems were minimized in a stepwise fashion. In the first minimization step, a 500 kcal mol^{-1} $Å^{-2}$ restraint was placed on the DNA, and 500 steps of steepest decent minimization and 500 steps of conjugate gradient minimization were performed. Subsequently, 1000 steps of unconstrained steepest decent minimization and 1500 steps of unconstrained conjugate gradient minimization were performed. The systems were then heated to 300 K over 20 ps using the Langevin thermostat (γ=1.0) with a 10 kcal mol^{-1} $Å^{-2}$ restraint on the DNA. Finally, each system was simulated for 100 ns at 300 K and 1 bar using the Langevin thermostat (γ=1.0), a Monte Carlo Barostat, and a time step of 2 fs. For all calculations, a non-bonded cutoff of 8.0 Å, the periodic boundary condition, and SHAKE were implemented. The simulations were run using the pmemd module of AMBER 14. For all duplexes, 20 ns pre-production simulations were performed to understand the inherent conformational flexibility of the lesion. From these trial simulations, representative structures for each unique lesion conformation were chosen as starting points for further simulations. Three 100 ns MD simulations were run using different starting velocities to ensure the results are statistically significantly. These simulations resulted in negligible all-atom rmsds between the replicas (Table S1). Therefore, one replica for each DNA duplex was selected for extension, first to 300 ns and subsequently to 500 ns. No significant differences were observed between the 300 and 500 ns simulations (Tables S3-S6 and Tables S12-S15), which suggests that the simulations are converged. Only data from the final 500 ns production simulation will be discussed throughout the main text.

Polymerase η model: Initial structures for simulations on the polymerase η insertion complexes were obtained from a crystal structure of the reactant complex for the insertion of dATP opposite dT (PDB ID: 4ECS). This crystal structure was chosen due to the resolution, presence of the two ions in the active site, and lack of mutation to the dNTP or 3′ terminus. Missing residues (Thr155–Glu159) were added by hand using GaussView. Glycerol, Ca^{2+}, and pyrophosphate were removed from the system. In cases were multiple orientations of amino acids were present in the crystal structure, the orientation that was best aligned to interact with the surrounding residues was chosen. In cases were the amino acid was not positioned to interact with any surrounding residues, the higher occupied crystal structure orientation was chosen. To generate the insertion complexes, dCTP, dTTP, or dATP was paired opposite POB-dG or PHB-dG in the active site based on the base pair hydrogen-bonding patterns observed in the DNA helix.

Additionally, the insertion complex for dCTP insertion opposite G was modeled as a control. The resulting 7 complexes were prepared for simulation using the tleap module of AMBER14. Specifically, hydrogen atoms were added to generate the native protonation states of all DNA and protein residues. Furthermore, NaCl was added to the water box to neutralize the system and to yield a final concentration of ~0.150 M (67 Na^+ ions and 56 Cl^- ions), and the system was solvated in a TIP3P octahedral water box such that the DNA–polymerase complex was at least 10.0 Å from the edge of the box.

The systems were minimized in a stepwise fashion. The first step minimized the water for 2500 steps of steepest decent and 2500 steps of conjugant gradient minimization, with a 50 kcal mol^{-1} $Å^{-2}$ on the rest of the system. Next, the hydrogen atoms were minimized for 4000 steps of steepest decent and 4000 steps of conjugant gradient minimization, with a 50 kcal mol^{-1} $Å^{-2}$ on the heavy atoms. Subsequently, the systems were minimized using a 50 kcal mol^{-1} $Å^{-2}$ restraint on the amino acid backbone for 12500 steps of steepest decent and 12500 steps of conjugant gradient minimization. Finally, the entire system was minimized without restraint for 5000 steps of steepest decent and 5000 steps of conjugant gradient minimization. The systems were then heated from 10 to 310 K in 6 steps, each increasing the temperature by 50 K over 10 ps using a 1 fs time step. Heating was performed with a 10 kcal mol^{-1} $Å^{-2}$ restraint on the solute using the Langevin thermostat (γ=1.0). The systems were then equilibrated over 5 steps, each of 20 ps, using a 2 fs time step, and a constraint of 20, 15, 10, 5, or 1 kcal mol^{-1} $Å^{-2}$ on the solute. Finally, a 100 ns unconstrained production simulation was performed on each system. MD simulations were run in triplicate using different initial velocities to ensure the results were statistically significantly. Due to negligible all-atom rmsds between each replica (Table S1), one simulation for each lesion–dNTP base pairing combination was extended to 500 ns. Since the conclusions remained the same upon extension (Tables S7-S9 and Tables S16-S18), the simulations are sufficiently converged. Data from the final 500 ns production simulation will be discussed throughout the main text.

Analysis: Analysis of the MD simulations was performed every 0.1 ns using the cpptraj module of Amber 14 (14). Additionally, 3DNA was used to understand the overall structure of the DNA helix (15). A representative structure for each MD simulation were obtained by clustering the simulations based on the rmsd of damaged base pair using the average linkage algorithm. Furthermore, the strength of the lesion hydrogen bonding with the pairing base in the DNA and polymerase models was calculated over 100 frames (i.e., every 3 ns for DNA models or 1 ns for polymerase models) using B3LYP-D3(BJ)/6-311+G(2df,2p) based on the geometry of the base pair in 100 representative structures sampled over the simulation trajectory, with hydrogen-capped nucleobases. When a hydrogen bond periodically exists between the bulky moiety and opposing base, the binding strength was separated into two clusters based on the presence or absence of the additional interaction. While we recognize the inherent errors that may be present in these hydrogen-bonding energies, this data allows for qualitative comparisons of the different base pairs.

References

1. HyperChem. Professional 7.5 ed. Hypercube, Inc., Gainesville, Florida 32601, USA.
2. Wilson, K.A., Szemethy, K.G. and Wetmore, S.D. (2017) Conformational flexibility and base-pairing tendency of the tobacco carcinogen o6-[4-oxo-4-(3-pyridyl)butyl]guanine. *Biophysical Chemistry*, **228**, 25-37.
3. Frisch, M.J., Trucks, G.W., Schlegel, H.B., Scuseria, G.E., Robb, M.A., Cheeseman, J.R., Scalmani, G., Barone, V., Mennucci, B., Petersson, G.A., Nakatsuji, H., Caricato, M., Li, X., Hratchian, H.P., Izmaylov, A.F., Bloino, J., Zheng, G., Sonnenberg, J.L., Hada, M., Ehara, M., Toyota, K., Fukuda, R.,

Hasegawa, J., Ishida, M., Nakajima, T., Honda, Y., Kitao, O., Nakai, H., Vreven, T., Jr., J.A.M., Peralta, J.E., Ogliaro, F., Bearpark, M., Heyd, J.J., Brothers, E., Kudin, K.N., Staroverov, V.N., Keith, T., Kobayashi, R., Normand, J., Raghavchari, K., Rendell, A., Burant, J.C., Iyengar, S.S., Tomasi, J., Cossi, M., Rega, N., Millam, J.M., Klene, M., Knox, J.E., Cross, J.B., Bakken, V., Adamo, C., Jaramillo, J., Gomperts, R., Stratmann, R.E., Yazyev, O., Austin, A.J., Cammi, R., Pomelli, C., Ochterski, J.W., Martin, R.L., Morokuma, K., Zakrzewski, V.G., Voth, G.A., Salvador, P., Dannenberg, J.J., Dapprich, S., Daniels, A.D., Farkas, O., Foresman, J.B., Ortiz, J.V., Cioslowski, J. and Fox, D.J. (2013). Revision D.01 ed. Gaussian, Inc., Wallingford CT.

4. Maier, J.A., Martinez, C., Kasavajhala, K., Wickstrom, L., Hauser, K.E. and Simmerling, C. (2015) Ff14sb: Improving the accuracy of protein side chain and backbone parameters from ff99sb. *J Chem Theory Comput*, **11**, 3696-3713.
5. Cornell, W.D., Cieplak, P., Bayly, C.I., Gould, I.R., Merz, K.M., Ferguson, D.M., Spellmeyer, D.C., Fox, T., Caldwell, J.W. and Kollman, P.A. (1995) A 2nd generation force-field for the simulation of proteins, nucleic-acids, and organic-molecules. *J. Am. Chem. Soc.*, **117**, 5179–5197.
6. Wang, J., Wang, W., Kollman, P.A. and Case, D.A. (2006) Automatic atom type and bond type perception in molecular mechanical calculations. *J. Mol. Graphics Modell.*, **25**, 247–260.
7. Dupradeau, F.-Y., Pigache, A., Zaffran, T., Savineau, C., Lelong, R., Grivel, N., Lelong, D., Rosanski, W. and Cieplak, P. (2010) The R.E.D. Tools: Advances in RESP and ESP charge derivation and force field library building. *Phys. Chem. Chem. Phys.*, **12**, 7821-7839.
8. Vanquelef, E., Simon, S., Marquant, G., Garcia, E., Klimerak, G., Delepine, J.C., Cieplak, P. and Dupradeau, F.-Y. (2011) R.E.D. Server: A web service for deriving RESP and ESP charges and building force field libraries for new molecules and molecular fragments. *Nucleic Acids Res.*, **39**, W511-W517.
9. Zhang, L., Rechkoblit, O., Wang, L., Patel, D.J., Shapiro, R. and Broyde, S. (2006) Mutagenic nucleotide incorporation and hindered translocation by a food carcinogen c8-dG adduct in sulfolobus solfataricus P2 DNA polymerase IV (Dpo4): Modeling and dynamics studies. *Nucleic Acids Res.*, **34**, 3326-3337.
10. Perlow, R.A. and Broyde, S. (2002) Toward understanding the mutagenicity of an environmental carcinogen: Structural insights into nucleotide incorporation preferences. *J. Mol. Biol.*, **322**, 291-309.
11. Zhang, L., Shapiro, R. and Broyde, S. (2005) Molecular dynamics of a food carcinogen−DNA adduct in a replicative DNA polymerase suggest hindered nucleotide incorporation and extension. *Chem. Res. Toxicol.*, **18**, 1347-1363.
12. Allnér, O., Nilsson, L. and Villa, A. (2012) Magnesium ion–water coordination and exchange in biomolecular simulations. *J. Chem. Theory Comput.*, **8**, 1493-1502.
13. Joung, I.S. and Cheatham, T.E. (2008) Determination of alkali and halide monovalent ion parameters for use in explicitly solvated biomolecular simulations. *J. Phys. Chem. B*, **112**, 9020-9041.
14. Case, D.A., Babin, V., Berryman, J.T., Betz, R.M., Cai, Q., Cerutti, D.S., T.E. Cheatham, I., Darden, T.A., Duke, R.E., Gohlke, H., Goetz, A.W., Gusarov, S., Homeyer, N., Janowski, P., Kaus, J., Kolossváry, I., Kovalenko, A., Lee, T.S., LeGrand, S., Luchko, T., Luo, R., Madej, B., Merz, K.M., Paesani, F., Roe, D.R., Roitberg, A., Sagui, C., Salomon-Ferrer, R., Seabra, G., Simmerling, C.L., Smith, W., Swails, J., Walker, R.C., Wang, J., Wolf, R.M., Wu, X. and Kollman, P.A. (2014). University of California, San Francisco.
15. Lu, X.-J. and Olson, W.K. (2008) 3dna: A versatile, integrated software system for the analysis, rebuilding and visualization of three-dimensional nucleic-acid structures. *Nat. Protocols*, **3**, 1213-1227.

Table S1. Heavy atom rmsd (Å) with respect to the representative structure for Trial 1.

	Trial 1 (100 ns)[a]		Trial 1 (300 ns)[a]		Trial 1 (500 ns)[a]		Trial 2 (100ns) [a]		Trial 3 (100 ns)[a]	
	Lesion Site	Entire system	Lesion Site	Entire system	Lesion Site	Entire system	Lesion Site	Entire system	Lesion Site	Entire system
DNA POB-G:C	0.866±0.175	1.908±0.414	0.875±0.178	1.887±0.399	0.874±0.178	1.885±0.401	0.879±0.184	1.889±0.391	0.875±0.191	1.890±0.426
DNA PHB-G:C	1.330±0.184	2.222±0.401	1.331±0.209	2.249±0.456	1.281±0.214	2.217±0.446	1.374±0.223	2.180±0.411	1.308±0.225	2.200±0.429
DNA POB-G:T	1.089±0.183	2.317±0.463	1.095±0.191	2.327±0.453	1.116±0.201	2.336±0.453	1.086±0.210	2.340±0.466	1.126±0.199	2.393±0.453
DNA PHB-G:T	1.5578±0.239	2.389±0.525	1.581±0.249	2.468±0.550	1.577±0.253	2.494±0.559	1.157±0.250	2.501±0.571	1.569±0.239	2.878±0.611
DNA POB-G:A	0.846±0.213	1.937±0.355	1.442±0.727	2.175±0.508	1.821±0.781	2.326±0.530	1.859±0.344	0.766±0.164	0.791±0.196	1.928±0.372
DNA PHB-G:A	0.862±0.188	1.951±0.417	0.877±0.218	1.994±0.438	0.868±0.205	1.993±0.433	0.847±0.171	1.964±0.396	0.852±0.174	1.957±0.397
DNA POB-G:G	1.878±0.208	4.221±0.706	1.300±0.492	2.850±1.147	1.159±0.433	2.542±0.984	1.760±0.316	3.827±0.994	1.845±0.273	4.667±0.563
DNA PHB-G:G	2.222±0.187	6.035±0.814	2.244±0.208	5.772±0.805	2.251±0.207	5.645±0.800	2.231±0.197	6.052±0.697	1.620±0.229	2.621±0.871
Pol η PHB-G:C	0.747±0.131	2.010±0.209	–	–	1.393±0.413	2.550±0.333	1.411±0.415	2.256±0.182	0.941±0.140	2.330±0.230
Pol η POB-G:T	1.059±0.182	2.090±0.268	–	–	1.455±0.378	2.587±0.420	0.987±0.230	2.107±0.159	1.120±0.180	2.356±0.121
Pol η PHB-G:T	1.202±0.264	2.167±0.267	–	–	1.093±0.438	1.835±0.305	1.646±0.100	2.472±0.250	1.704± 0.097	2.522±0.184
Pol η POB-G:A	1.157±0.384	2.065±0.330	–	–	1.566±0.300	2.626±0.413	2.385±0.341	1.044±0.256	0.947±0.117	2.228±0.166
Pol η PHB-G:A	1.468±0.402	2.077±0.237	–	–	1.671±0.320	2.273±0.287	2.200±0.116	2.331±0.165	2.197±0.228	2.283±0.130

[a]MD simulation length provided in brackets.

Table S2. B3LYP-D3(BJ)/6-311+G(2df,2p)//M06-2X/6-31G(d) interaction energies (kJ/mol) for base pairs between the Watson-Crick or Hoogsteen hydrogen-bonding face of G, or various POB-G or PHB-G conformers (G*), and the canonical nucleobases.[a]

		Fully Extended		Stacked		Hydrogen Bonded		T-shaped		Extended	
	G	POB-G	PHB-G	POB-G	PHB-G	POB-G	PHB-G	POB-G	PHB-G	POB-G	PHB-G
Watson-Crick G*:C	–138.0	–80.5	–72.5	–79.9	–75.8	N/A[b]	N/A[b]	N/A[b]	N/A[b]	–64.4	–57.7
Watson-Crick G*:T	–80.4	–69.7	–55.6	–68.9	–75.4	N/A[b]	N/A[b]	N/A[b]	N/A[b]	–56.3	–52.8
Watson-Crick G*:A	–82.5	–64.3	–59.8	–64.7	–65.9	N/A[b]	N/A[b]	N/A[b]	N/A[b]	–50.6	–48.2
Watson-Crick G*:G	–69.3	–38.1	–29.8	–48.4	–34.6	N/A[b]	N/A[b]	N/A[b]	N/A[b]	–43.1	–36.4
Hoogsteen G*:C	–59.0	–54.6	–53.1	–59.3	–63.3	–59.3	–53.1	–58.4	–60.0	N/A[b]	N/A[b]
Hoogsteen G*:T	–42.4	–52.4	–50.6	–59.5	–69.9	–51.5	–46.9	–50.9	–71.1	N/A[b]	N/A[b]
Hoogsteen G*:A	–44.4	-42.7	–43.6	–43.2	–51.7	–43.3	–38.9	–41.4	–58.6	N/A[b]	N/A[b]
Hoogsteen G*:G	–69.3	–73.8	–93.9	–76.5	–71.9	–78.7	–68.6	–84.2	–78.0	N/A[b]	N/A[b]

[a]See Figures 3 and S5–S12. [b]Not applicable due to bulky moiety interactions with the respective hydrogen-bonding face.

Table S3. Occupancies (%), average heavy atom distances (Å), and average angles (deg.) for hydrogen bonds in the adducted, and 3′ and 5′-flanking base pairs across the entire 500ns MD simulation trajectory for POB-G or PHB-G adducted DNA with the lesion paired opposite C.

	POB-G:C Occupancy	Average Distance	Average Angle	PHB-G:C Occupancy	Average Distance	Average Angle
5′-C(O2)⋯GN2H)	100%	2.882	163.7	100%	2.877	163.0
5′-C(N3)⋯G(N1)	100%	2.962	165.5	100%	2.963	164.9
5′-C(N4H)⋯G(O6)	99%	2.924	163.6	99%	2.934	162.1
G*(N1)⋯C(N4H)	86%	3.120	159.4	96%	3.019	163.5
G*(N2H)⋯C(N3)	91%	3.061	159.8	92%	3.034	160.8
G*(O12)⋯C(N4H)	21%	2.952	161.2	5%	2.980	159.2
3′-C(O2)⋯G(N2H)	100%	2.861	164.2	100%	2.880	163.2
3′-C(N3)⋯G(N1)	100%	2.951	164.2	100%	2.950	163.9
3′-C(N4H)⋯G(O6)	99%	2.937	164.3	99%	2.934	163.8

[a]Hydrogen-bonding occupancies are based on a distance cutoff of < 3.4 Å and an angle cutoff of < 120°.

Table S4. Occupancies (%), average heavy atom distances (Å), and average angles (deg.) for hydrogen bonds in the adducted, and 3′ and 5′-flanking base pairs across the entire 500 ns MD simulation trajectory for POB-G or PHB-G adducted DNA with the lesion paired opposite T.

	POB-G:T Occupancy	Average Distance	Average Angle	PHB-G:T Occupancy	Average Distance	Average Angle
5′-C(O2)···G(N2H)	100%	2.878	163.9	100%	2.874	163.8
5′-C(N3)···G(N1)	100%	2.955	165.2	100%	2.954	165.1
5′-C(N4H)···G(O6)	99%	2.917	163.6	99%	2.919	162.9
G*(N1)···T(N3H)	34%	3.204	155.0	60%	3.138	156.8
G*(N2H)···T(O2)	94%	2.927	158.1	94%	2.892	158.2
G*(O12)···5′-C(N4H)	11%	2.989	154.7	5%	3.068	155.3
3′-C(O2)···G(N2H)	100%	2.861	163.7	100%	2.868	163.3
3′-C(N3)···G(N1)	100%	2.953	165.1	100%	2.954	164.9
3′-C(N4H)···G(O6)	98%	2.953	163.6	98%	2.946	163.4

[a]Hydrogen-bonding occupancies are based on a distance cutoff of < 3.4 Å and an angle cutoff of < 120°.

Table S5. Occupancies (%), average heavy atom distances (Å), and average angles (deg.) for hydrogen bonds in the adducted, and 3′ and 5′-flanking base pairs across the entire 500 ns MD simulation trajectory for POB-G or PHB-G adducted DNA with the lesion paired opposite A.

	POB-G:A			PHB-G:A		
	Occupancy	Average Distance	Average Angle	Occupancy	Average Distance	Average Angle
5′-C(O2)···G(N2H)	100%	2.890	163.5	100%	2.885	163.4
5′-C(N3)···G(N1)	100%	2.963	164.9	100%	2.966	165.1
5′-C(N4H)···G(O6)	99%	2.929	162.8	99%	2.929	162.3
G*(N1)···A(N6H)	30%	3.143	159.8	97%	3.028	161.4
G*(N2H)···A(N7)	22%	3.040	160.2	95%	3.026	158.6
3′-C(O2)···G(N2H)	100%	2.878	163.7	100%	2.858	164.0
3′-C(N3)···G(N1)	100%	2.960	164.6	100%	2.957	164.7
3′-C(N4H)···G(O6)	98%	2.948	163.6	98%	2.962	163.7

[a]Hydrogen-bonding occupancies are based on a distance cutoff of < 3.4 Å and an angle cutoff of < 120°.

Table S6. Occupancies (%), average heavy atom distances (Å), and average angles (deg.) for hydrogen bonds in the adducted, and 3′ and 5′-flanking base pairs across the entire 500 ns MD simulation trajectory for POB-G or PHB-G adducted DNA with the lesion paired opposite G.

	POB-G:G			PHB-G:G		
	Occupancy	Average Distance	Average Angle	Occupancy	Average Distance	Average Angle
5′-C(O2)···G(N2H)	100%	2.904	165.4	100%	2.885	164.1
5′-C(N3)···G(N1)	100%	2.965	165.9	100%	2.958	165.0
5′-C(N4H)···G(O6)	99%	2.922	164.9	99%	2.931	163.2
G*(N7)···G(N2H)	91%	3.002	154.1	92%	2.967	156.5
G*(N7)···G(N1H)	7%	3.213	140.9	25%	3.194	143.0
G*(O6)···G(N1H)	3%	3.195	151.0	26%	3.180	157.6
3′-C(O2)···G(N2H)	100%	2.848	164.1	100%	2.883	162.8
3′-C(N3)···G(N1)	100%	2.953	164.4	100%	2.961	165.3
3′-C(N4H)···G(O6)	95%	2.980	162.7	98%	2.940	163.0

[a]Hydrogen-bonding occupancies are based on a distance cutoff of < 3.4 Å and an angle cutoff of < 120°. [b]Not Observed.

Table S7. Occupancies (%), average heavy atom distances (Å), and average angles (deg.) for the hydrogen bonds between the template G, POB-G, PHB-G or opposing dCTP and the surrounding DNA:polymerase η complex across the entire 500 ns MD simulation on the polymerase η insertion complex.

	G:dCTP			POB-G:dCTP			PHB-G:dCTP		
	Occupancy	Average Distance	Average Angle	Occupancy	Average Distance	Average Angle	Occupancy	Average Distance	Average Angle
3′-T(O4)···A(N6H)	95%	2.989	156.8	93%	3.014	156.9	79%	2.980	164.2
3′-T(N3H)···A(N1)	100%	2.937	163.3	100%	2.933	159.5	81%	2.943	163.9
dCTP(O2)···G(N2H)	100%	2.839	159.8	NO[b]			NO[b]		
dCTP(N3)···G(N1H)	100%	2.933	160.3	NO[b]			NO[b]		
dCTP(N4H)···G(O6)	100%	2.937	163.3	NO[b]			NO[b]		
dCTP(N3)···G*(N2H)	NO[b]			52%	3.027	153.5	51%	3.009	161.9
dCTP(N4H)···G*(N1)	NO[b]			22%	3.194	143.5	24%	3.130	160.1
dCTP(O3')···Phe18(NH)	95%	3.077	161.1	84%	3.073	162.9	78%	3.075	164.7
dCTP(Oα1)···Arg61(NH1H)	76%	2.83	157.5	30%	2.825	158.8	40%	2.805	161.9
dCTP(Oβ2)···Phe17(NH)	84%	3.193	157.6	79%	3.189	159.4	61%	3.192	164.1
dCTP(Oβγ)···Arg55(NH2H)	96%	2.942	144.4	97%	2.910	151.1	53%	2.912	163.9
dCTP(Oγ3)···Tyr52(OH)	97%	2.586	165.5	94%	2.583	165.9	95%	2.593	166.5
dCTP(Oγ3)···Lys231(NZH)	61%	2.917	144.5	53%	2.942	141.2	31%	2.953	163.2
dCTP(Oγ1)···Lys231(NZH)	30%	3.074	147.1	41%	3.000	155.0	NO[b]		
dCTP(Oγ3)···Cys16(NH)	100%	2.92	166.8	98%	2.942	165.6	93%	2.939	165.5
dCTP(Oγ3)···Arg55(NH2H)	90%	2.939	149.8	67%	2.992	146.3	32%	2.852	160.1
dCTP(Oγ3)···Arg55(NH1H)	74%	3.041	143	68%	3.014	155.4	NO[b]		
dCTP(Oγ2)···Arg55(NH1H)	67%	3.077	152.3	62%	3.020	146.1	46%	2.879	161.7

[a]Hydrogen-bonding occupancies are based on a distance cutoff of < 3.4 Å and an angle cutoff of < 120°. [b]Not Observed.

Table S8. Occupancies (%), average heavy atom distances (Å), and average angles (deg.) for the hydrogen bonds between the lesion or opposing dTTP and the surrounding DNA:polymerase η complex across the entire 500 ns MD simulation on the polymerase η insertion complex.

	POB-G:dTTP			PHB-G:dTTP		
	Occupancy	Average Distance	Average Angle	Occupancy	Average Distance	Average Angle
3′-T(O4)···A(N6H)	95%	2.972	157.4	94%	3.001	158.3
3′-T(N3H)···A(N1)	100%	2.954	161.4	100%	2.926	160.6
dTTP(O2)···G*(N2H)	39%	2.948	157.4	49%	2.932	155.2
dTTP(N3H)···G*(N1)	17%	3.187	140.3	25%	3.176	145.4
dTTP(O4)···G*(O13H)	NO[b]			40%	2.751	162.6
dTTP(O3')···Phe18(NH)	46%	3.070	163.7	56%	3.159	162.5
dTTP(O4)···Arg61(NH1H)	26%	2.813	155.2	NO[b]		
dTTP(Oβ1)···Arg61(NH1H)	NO[b]			75%	2.928	159.0
dTTP(Oβ1)···Arg61(NH2H)	NO[b]			51%	3.136	140.6
dTTP(Oβ2)···Phe17(NH)	69%	3.196	159.4	77%	3.162	158.4
dTTP(Oβγ)···Arg55(NH2H)	97%	2.920	151.4	92%	2.968	143.6
dTTP(Oγ3)···Tyr52(OH)	94%	2.583	165.1	56%	3.159	162.5
dTTP(Oγ3)···Lys231(NZH)	50%	2.954	154.8	35%	2.903	153.8
dTTP(Oγ3)···Cys16(NH)	96%	2.969	165.2	99%	2.923	166.0
dTTP(Oγ2)···Arg55(NH1H)	68%	2.996	153.8	47%	3.084	148.2
dTTP(Oγ3)···Arg55(NH1H)	61%	2.995	147.2	85%	2.912	151.2
dTTP(Oγ3)···Arg55(NH2H)	60%	3.006	147.7	82%	3.000	146.8

[a]Hydrogen-bonding occupancies are based on a distance cutoff of < 3.4 Å and an angle cutoff of < 120°. [b]Not Observed.

Table S9. Occupancies (%), average heavy atom distances (Å), and average angles (deg.) for the hydrogen bonds between the lesion or opposing dATP and the surrounding DNA:polymerase η complex across the entire 500 ns MD simulation on the polymerase η insertion complex.

	POB-G:ATP			PHB-G:ATP		
	Occupancy	Average Distance	Average Angle	Occupancy	Average Distance	Average Angle
3′-T(O4)···A(N6H)	91%	3.037	154.1	88%	3.033	152.7
3′-T(N3H)···A(N1)	100%	2.916	162.4	100%	2.922	161.8
dATP(N6H)···G*(N3)	51%	3.100	144.6	32%	3.124	148.0
dATP(N7)···G*(N2H)	49%	3.015	150.1	7%	3.023	154.4
dATP(N1)···G*(N2H)	41%	3.152	155.0	16%	3.218	138.4
dATP(O3')···Phe18(NH)	80%	3.145	167.3	77%	3.159	167.0
dATP(N1)···Arg61(NH1H)	20%	2.886	159.8	12%	2.891	160.5
dATP(Oβ2)···Phe17(NH)	92%	3.161	160.2	92%	3.149	160.8
dATP(Oβγ)···Arg55(NH1H)	95%	2.968	147.8	94%	2.951	149.0
dATP(Oγ3)···Tyr52(OH)	80%	2.607	166.4	54%	2.613	166.0
dATP(Oγ3)···Lys231(NZH)	31%	3.049	154.9	30%	2.932	141.8
dATP(Oγ1)···Lys231(NZH)	66%	2.904	143.3	59%	2.908	156.8
dATP(Oγ3)···Cys16(NH)	99%	2.922	165.1	99%	2.934	165.2
dATP(Oγ3)···Arg55(NH1H)	60%	2.992	149.2	61%	3.007	148.9
dATP(Oγ3)···Arg55(NH2H)	47%	3.064	142.5	53%	3.063	143.0
dATP(Oγ2)···Arg55(NH2H)	85%	2.946	156.3	86%	2.950	154.5

[a]Hydrogen-bonding occupancies are based on a distance cutoff of < 3.4 Å and an angle cutoff of < 120°. [b]Not Observed.

Table S10. Coordination of the active site Mg^{2+} ions across the entire MD simulation trajectory for the polymerase η insertion complex corresponding to various lesion replication outcomes.[a]

	Mg^{2+} (binding)	Mg^{2+} (catalytic)
G:dCTP	6/6	6/6
POB-G:dCTP	5/6 (Oα2, 57%)[b]	6/6
PHB-G:dCTP	5/6 (Oα2, 62%)[b]	6/6
POB-G:dTTP	5/6 (Oα2, 76%)[b]	6/6
PHB-G:dTTP	5/6 (Oα2, 49%)[b]	6/6
POB-G:dATP	5/6 (Oα2, 88%)[b]	6/6
PHB-G:dATP	5/6 (Oα2, 86%)[b]	6/6

[a]Coordination is considered to be present if the distance is < 2.5 Å for > 95% of the simulation. [b]Atom that does not meet coordination criteria and percentage of simulation it meets the coordination criteria is given in brackets.

Table S11. Atom types and charges for POB-G and PHB-G.

Atom Name	Atom Type	POB-G Charge	PHB-G Charge
P	P	1.1617	1.1624
O5'	OS	-0.4974	-0.4949
OP1	O2	-0.7662	-0.7629
OP2	O2	-0.7662	-0.7698
O3'	OS	-0.5408	-0.5444
C5'	CT	-0.0247	0.001
H5'1	H1	0.0806	0.072
H5'2	H1	0.0806	0.072
C4'	CT	0.148	0.1731
H4'	H1	0.0874	0.1016
O4'	OS	-0.3754	-0.4053
C1'	CT	0.1532	0.2091
H1'	H2	0.0809	0.0431
C3'	CT	0.2336	0.1433
H3'	H1	0.0363	0.0719
C2'	CT	-0.0405	-0.0565
H2'1	HC	0.0287	0.0445
H2'2	HC	0.0287	0.0445
N9	N*	-0.0791	-0.0438
N1	NC	-0.4172	-0.7314
C2	CQ	0.6269	0.8253
N2	N2	-0.9021	-0.9516
H21	H	0.3758	0.4031
H22	H	0.3758	0.4031
N3	NC	-0.5135	-0.588
C4	CB	0.2216	0.2658
C5	CB	0.1513	0.0834
C6	CA	0.3917	0.6332
O6	OS	-0.3159	-0.4136
N7	NB	-0.5899	-0.5995
C8	CK	0.2068	0.1512
H8	H5	0.1357	0.1618
C9	CT	0.0249	0.1154
H91	H1	0.0718	0.0654
H92	H1	0.0718	0.0654
C10	CT	0.0199	0.0326
H101	HC	0.0373	0.0313
H102	HC	0.0373	0.0313
C11	CT	-0.0959	-0.0029
H111	HC	0.0497	0.0125
H112	HC	0.0497	0.0125
C13	CA	-0.0585	-0.0502
C14	CA	-0.0378	-0.0548
H14	HA	0.1577	0.135
C15	CA	-0.2635	-0.3221
H15	HA	0.1481	0.1541
C16	CA	0.2165	0.2986
H16	H4	0.0964	0.07
C17	CA	0.2337	0.2679
H17	H4	0.0431	0.0862
N18	NC	-0.4886	-0.5909
C12	C (CT)[a]	0.4018	0.1062
O12	O (OH)[a]	-0.4907	-0.6278
H12'	(HO)[a]	N/A	0.4061
H12	(H1)[a]	N/A	0.0545

[a]Atom types for PHB-G are given in brackets

Table S12. Occupancies (%), average heavy atom distances (Å), and average angles (deg.) for hydrogen bonds in the adducted, and 3′ and 5′-flanking base pairs across the entire 300 ns MD simulation trajectory for POB-G or PHB-G adducted DNA with the lesion paired opposite C.

	POB-G:C			PHB-G:C		
	Occupancy	Average Distance	Average Angle	Occupancy	Average Distance	Average Angle
5′-C(O2)···GN2H)	100%	2.882	163.7	100%	2.877	163.0
5′-C(N3)···G(N1)	100%	2.962	165.5	100%	2.963	164.8
5′-C(N4H)···G(O6)	99%	2.924	163.7	99%	2.934	162.2
G*(N1)···C(N4H)	86%	3.120	159.4	96%	3.020	163.4
G*(N2H)···C(N3)	91%	3.061	159.7	92%	3.035	160.8
G*(O12)···C(N4H)	29%	2.961	158.0	7%	2.977	159.0
3′-C(O2)···G(N2H)	100%	2.861	164.3	100%	2.882	163.1
3′-C(N3)···G(N1)	100%	2.952	164.3	100%	2.950	163.9
3′-C(N4H)···G(O6)	99%	2.937	164.3	99%	2.933	163.8

[a]Hydrogen-bonding occupancies are based on a distance cutoff of < 3.4 Å and an angle cutoff of < 120°.

Table S13. Occupancies (%), average heavy atom distances (Å), and average angles (deg.) for hydrogen bonds in the adducted, and 3′ and 5′-flanking base pairs across the entire 300 ns MD simulation trajectory for POB-G or PHB-G adducted DNA with the lesion paired opposite T.

	POB-G:T Occupancy	Average Distance	Average Angle	PHB-G:T Occupancy	Average Distance	Average Angle
5′-C(O2)···G(N2H)	100%	2.878	163.9	100%	2.873	163.8
5′-C(N3)···G(N1)	100%	2.955	165.3	100%	2.955	165.1
5′-C(N4H)···G(O6)	99%	2.917	163.6	99%	2.919	162.9
G*(N1)···T(N3H)	32%	3.208	154.7	63%	3.136	156.8
G*(N2H)···T(O2)	94%	2.924	158.1	93%	2.892	158.1
G*(O12)···5′-C(N4H)	11%	2.988	154.4	6%	3.068	155.7
3′-C(O2)···G(N2H)	100%	2.861	163.7	100%	2.869	163.2
3′-C(N3)···G(N1)	100%	2.954	165.1	100%	2.954	164.9
3′-C(N4H)···G(O6)	98%	2.953	163.6	98%	2.946	163.4

[a]Hydrogen-bonding occupancies are based on a distance cutoff of < 3.4 Å and an angle cutoff of < 120°.

Table S14. Occupancies (%), average heavy atom distances (Å), and average angles (deg.) for hydrogen bonds in the adducted, and 3′ and 5′-flanking base pairs across the entire 300 ns MD simulation trajectory for POB-G or PHB-G adducted DNA with the lesion paired opposite A.

	POB-G:A			PHB-G:A		
	Occupancy	Average Distance	Average Angle	Occupancy	Average Distance	Average Angle
5′-C(O2)···G(N2H)	100%	2.889	163.4	100%	2.885	163.4
5′-C(N3)···G(N1)	100%	2.964	165.0	100%	2.966	165.2
5′-C(N4H)···G(O6)	99%	2.928	162.7	99%	2.929	162.4
G*(N1)···A(N6H)	51%	3.143	159.8	96%	3.029	161.4
G*(N2H)···A(N7)	37%	3.040	160.2	95%	3.025	158.5
3′-C(O2)···G(N2H)	100%	2.876	163.6	100%	2.860	164.0
3′-C(N3)···G(N1)	100%	2.958	164.2	100%	2.957	164.7
3′-C(N4H)···G(O6)	97%	2.951	163.4	97%	2.962	163.6

[a]Hydrogen-bonding occupancies are based on a distance cutoff of < 3.4 Å and an angle cutoff of < 120°.

Table S15. Occupancies (%), average heavy atom distances (Å), and average angles (deg.) for hydrogen bonds in the adducted, and 3′ and 5′-flanking base pairs across the entire 300 ns MD simulation trajectory for POB-G or PHB-G adducted DNA with the lesion paired opposite G.

	POB-G:G Occupancy	Average Distance	Average Angle	PHB-G:G Occupancy	Average Distance	Average Angle
5′-C(O2)···G(N2H)	100%	2.899	165.2	100%	2.883	164.2
5′-C(N3)···G(N1)	100%	2.965	165.7	100%	2.959	165.0
5′-C(N4H)···G(O6)	99%	2.923	164.5	99%	2.930	163.3
G*(N7)···G(N2H)	92%	2.997	154.9	90%	2.967	156.7
G*(N7)···G(N1H)	10%	3.208	141.7	NO[b]		
G*(O6)···G(N1H)	4%	3.191	151.9	48%	3.189	150.1
3′-C(O2)···G(N2H)	100%	2.854	163.9	100%	2.884	162.9
3′-C(N3)···G(N1)	100%	2.954	164.6	100%	2.960	165.3
3′-C(N4H)···G(O6)	96%	2.974	162.6	98%	2.940	162.8

[a]Hydrogen-bonding occupancies are based on a distance cutoff of < 3.4 Å and an angle cutoff of < 120°. [b]Not Observed.

Table S16. Occupancies (%), average heavy atom distances (Å), and average angles (deg.) for the hydrogen bonds between the template G, POB-G, PHB-G or opposing dCTP and the surrounding DNA:polymerase η complex across the entire 100 ns MD simulation on the polymerase η insertion complex.

	G:dCTP			POB-G:dCTP			PHB-G:dCTP		
	Occupancy	Average Distance	Average Angle	Occupancy	Average Distance	Average Angle	Occupancy	Average Distance	Average Angle
3′-T(O4)···A(N6H)	95%	2.989	156.8	95%	2.99	157	82%	2.972	164.4
3′-T(N3H)···A(N1)	100%	2.937	163.3	100%	2.934	159.2	86%	2.946	164.1
dCTP(O2)···G(N2H)	100%	2.839	159.8	NO[b]			NO[b]		
dCTP(N3)···G(N1H)	100%	2.933	160.3	NO[b]			NO[b]		
dCTP(N4H)···G(O6)	100%	2.937	163.3	NO[b]			NO[b]		
dCTP(N3)···G*(N2H)	NO[b]			97%	2.999	158.1	77%	3.025	162.3
dCTP(N4H)···G*(N1)	NO[b]			53%	3.182	141.8	39%	3.117	159.7
dCTP(O3')···Phe18(NH)	95%	3.077	161.1	94%	3.058	163.1	83%	3.053	164.4
dCTP(Oα1)···Arg61(NH1H)	76%	2.83	157.5	90%	2.813	159.5	71%	2.802	161.3
dCTP(Oβ2)···Phe17(NH)	84%	3.193	157.6	83%	3.193	159.1	55%	3.227	164.1
dCTP(Oβγ)···Arg55(NH2H)	96%	2.942	144.4	97%	2.911	149.6	56%	2.883	161.5
dCTP(Oγ3)···Tyr52(OH)	97%	2.586	165.5	90%	2.588	165.7	98%	2.572	167.5
dCTP(Oγ3)···Lys231(NZH)	61%	2.917	144.5	57%	2.923	144.1	42%	2.988	163
dCTP(Oγ1)···Lys231(NZH)	30%	3.074	147.1	22%	3.006	153.7	13%	2.895	160.4
dCTP(Oγ3)···Cys16(NH)	100%	2.92	166.8	98%	2.95	165.8	95%	2.941	166
dCTP(Oγ3)···Arg55(NH2H)	90%	2.939	149.8	78%	2.993	146.4	22%	2.94	158.2
dCTP(Oγ3)···Arg55(NH1H)	74%	3.041	143	68%	3.007	145.6	13%	2.94	156.9
dCTP(Oγ2)···Arg55(NH1H)	67%	3.077	152.3	67%	3.007	154.9	60%	2.912	164.3

[a]Hydrogen-bonding occupancies are based on a distance cutoff of < 3.4 Å and an angle cutoff of < 120°. [b]Not Observed.

Table S17. Occupancies (%), average heavy atom distances (Å), and average angles (deg.) for the hydrogen bonds between the lesion or opposing dTTP and the surrounding DNA:polymerase η complex across the entire 100 ns MD simulation on the polymerase η insertion complex.

	POB-G:dTTP			PHB-G:dTTP		
	Occupancy	Average Distance	Average Angle	Occupancy	Average Distance	Average Angle
3′-T(O4)···A(N6H)	97%	2.997	156.8	96%	2.994	159.5
3′-T(N3H)···A(N1)	100%	2.942	161.3	100%	2.936	160.4
dTTP(O2)···G*(N2H)	93%	2.939	157.2	96%	2.933	154.1
dTTP(N3H)···G*(N1)	50%	3.187	141.5	41%	3.162	144.6
dTTP(O4)···G*(O13H)	NO[b]			24%	2.751	162.6
dTTP(O3')···Phe18(NH)	90%	3.089	163.4	59%	3.123	162.8
dTTP(O4)···Arg61(NH1H)	28%	2.828	157.2	NO[b]		
dTTP(Oβ1)···Arg61(NH1H)	NO[b]			57%	2.936	159
dTTP(Oβ1)···Arg61(NH2H)	NO[b]			36%	3.092	142.7
dTTP(Oβ2)···Phe17(NH)	92%	3.154	159.9	73%	3.155	159.9
dTTP(Oβγ)···Arg55(NH2H)	99%	2.913	150.5	94%	2.948	146.6
dTTP(Oγ3)···Tyr52(OH)	76%	2.597	165	29%	2.584	164.3
dTTP(Oγ3)···Lys231(NZH)	NO[b]			52%	2.84	155.5
dTTP(Oγ3)···Cys16(NH)	95%	2.98	165.5	98%	2.958	165.5
dTTP(Oγ2)···Arg55(NH1H)	59%	3.002	154	51%	3.082	147.2
dTTP(Oγ3)···Arg55(NH1H)	65%	3.003	147.3	84%	2.98	148.9
dTTP(Oγ3)···Arg55(NH2H)	62%	2.988	149.5	77%	2.956	149.1

[a]Hydrogen-bonding occupancies are based on a distance cutoff of < 3.4 Å and an angle cutoff of < 120°. [b]Not Observed.

Table S18. Occupancies (%), average heavy atom distances (Å), and average angles (deg.) for the hydrogen bonds between the lesion or opposing dATP and the surrounding DNA:polymerase η complex across the entire 100 ns MD simulation on the polymerase η insertion complex.

	POB-G:ATP			PHB-G:ATP		
	Occupancy	Average Distance	Average Angle	Occupancy	Average Distance	Average Angle
3′-T(O4)···A(N6H)	93%	3.008	158.1	89%	3.016	155.5
3′-T(N3H)···A(N1)	100%	2.645	165.1	100%	2.933	161.6
dATP(N6H')···G*(N1)	29%	3.200	148.0	17%	3.156	147.3
dATP(N7)···G*(N2H)	55%	2.993	161.8	19%	3.020	154.6
dATP(N6H')···G*(N3)	12%	3.092	146.1	18%	3.110	148.3
dATP(O3')···Phe18(NH)	81%	3.143	167.5	74%	3.171	167.2
dATP(N1)···Arg61(NH1H)	18%	2.982	156.4	12%	2.892	158.8
dATP(Oβ2)···Phe17(NH)	90%	3.165	161.7	91%	3.142	161.1
dATP(Oβγ)···Arg55(NH1H)	98%	2.949	151.2	93%	2.968	147.1
dATP(Oγ3)···Tyr52(OH)	91%	2.609	167.3	74%	2.613	165.7
dATP(Oγ3)···Lys231(NZH)	62%	3.056	155.6	41%	2.987	155.7
dATP(Oγ1)···Lys231(NZH)	67%	2.898	140.4	56%	2.911	143.4
dATP(Oγ3)···Cys16(NH)	99%	2.941	164	99%	2.917	165.4
dATP(Oγ3)···Arg55(NH1H)	38%	3.213	139.2	68%	2.997	150.2
dATP(Oγ3)···Arg55(NH2H)	38%	3.045	148.8	54%	3.069	143.1
dATP(Oγ2)···Arg55(NH2H)	91%	2.906	159.8	84%	2.972	153.3

[a]Hydrogen-bonding occupancies are based on a distance cutoff of < 3.4 Å and an angle cutoff of < 120°. [b]Not Observed.

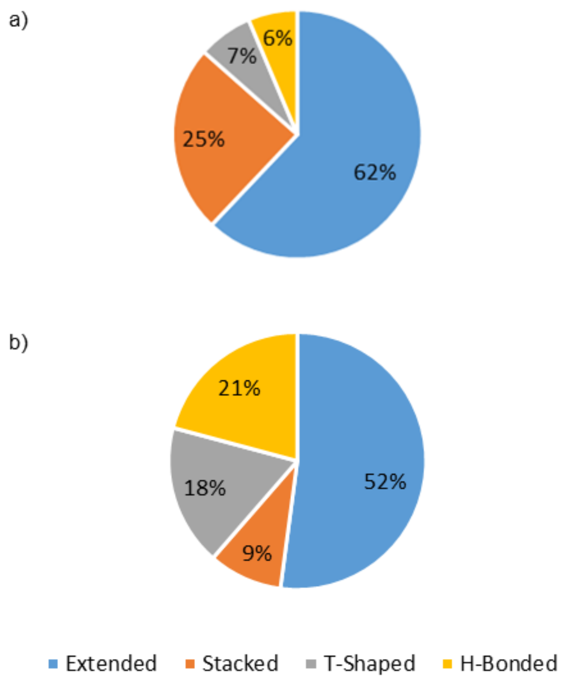

Figure S1. Distribution of the a) POB-G and b) PHB-G nucleobase conformations resulting from the DFT conformational search.

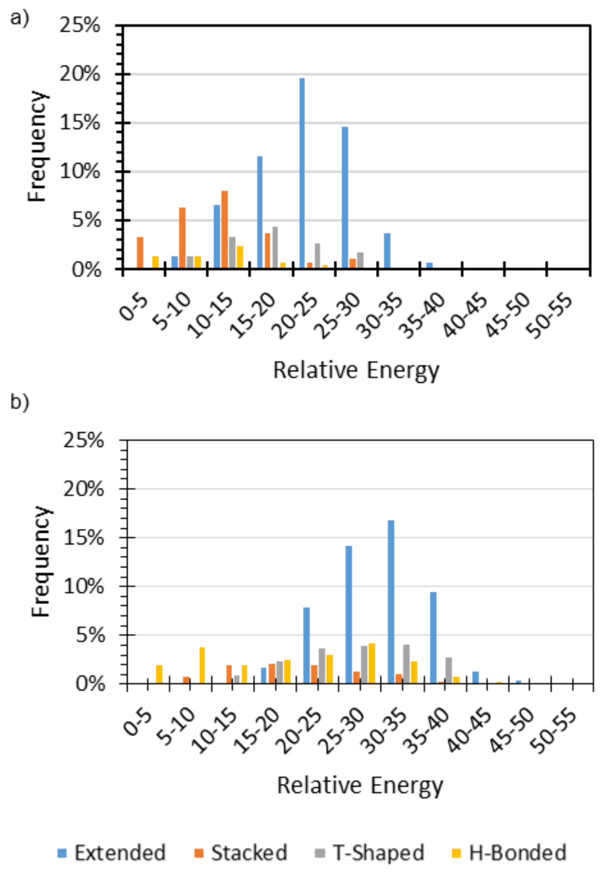

Figure S2. Distribution of the B3LYP-D3(BJ)/6-311+G(2df,2p) relative energies (kJ/mol) of the a) POB-G and b) PHB-G nucleobase conformations for each structural category.

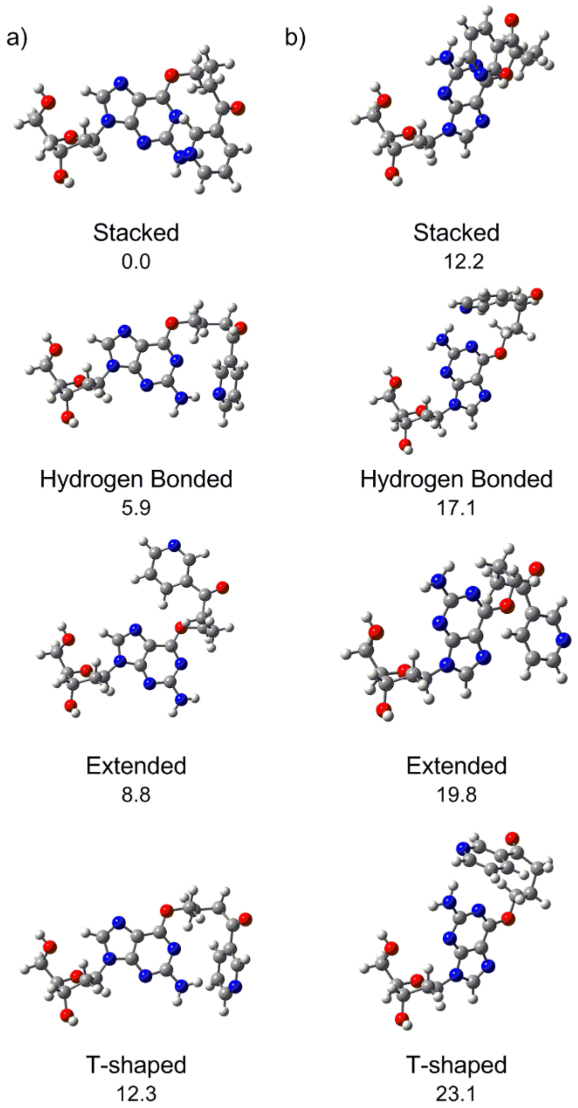

Figure S3. For each structural category B3LYP-D3(BJ)/6-311+G(2df,2p)//B3LYP-D3(BJ)/6-31G(d) a) *anti* and b) *syn* structures of the POB-G nucleoside, as well as the relative energies (kJ/mol).

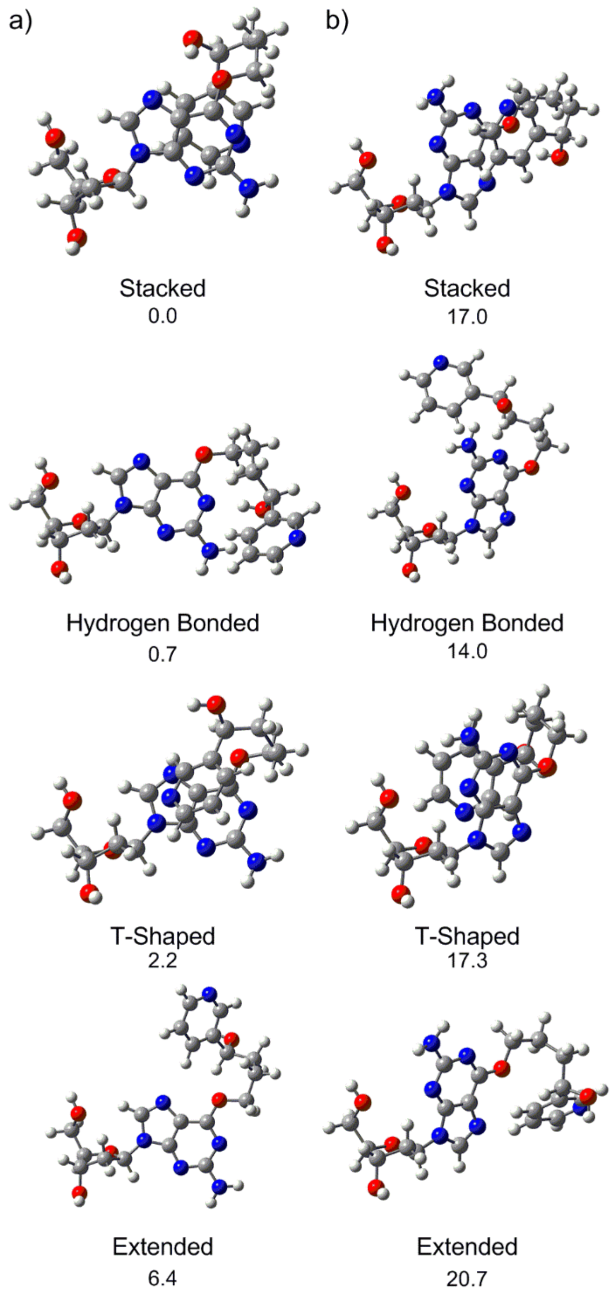

Figure S4. For each structural category B3LYP-D3(BJ)/6-311+G(2df,2p)//B3LYP-D3(BJ)/6-31G(d) a) *anti* and b) *syn* structures of the PHB-G nucleoside, as well as the relative energies (kJ/mol).

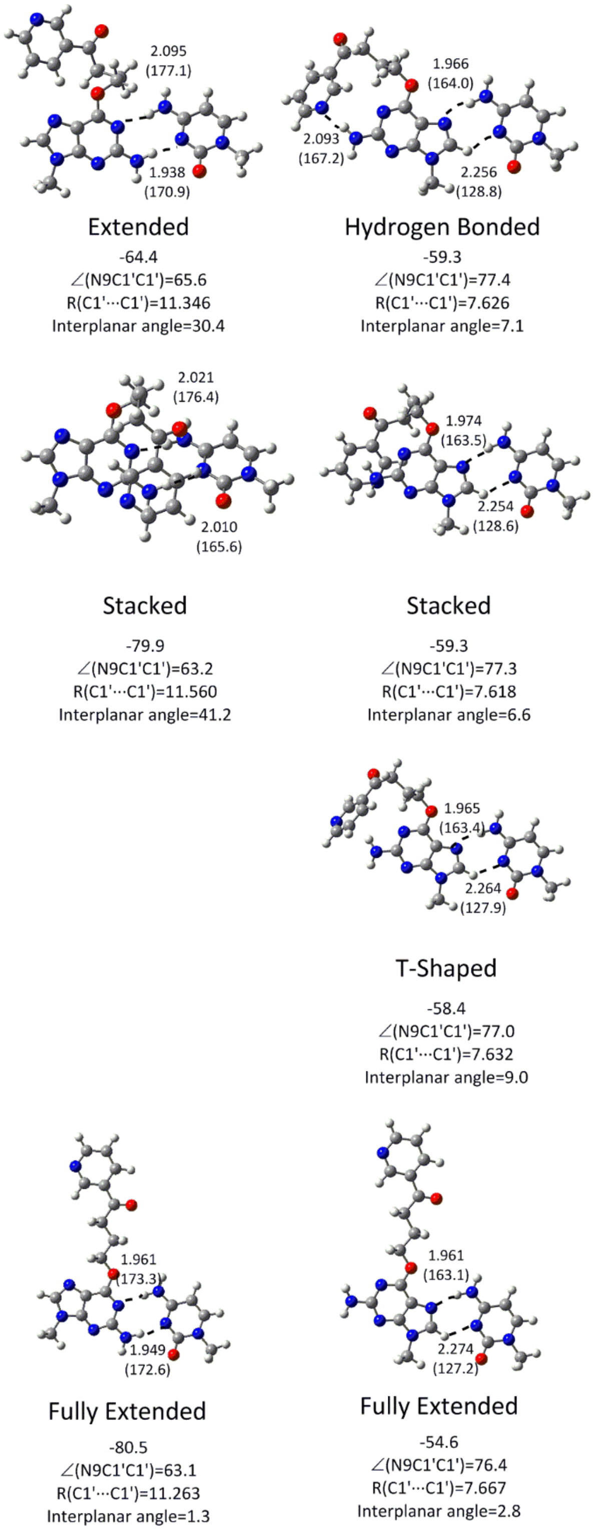

Figure S5. B3LYP-D3(BJ)/6-311+G(2df,2p)//M06-2X/6-31G(d) structures (distances in Å and angles in deg.) and binding energy (kJ/mol) for dimers between the Watson-Crick (left) or Hoogsteen (right) face of various POB-G conformations and C.

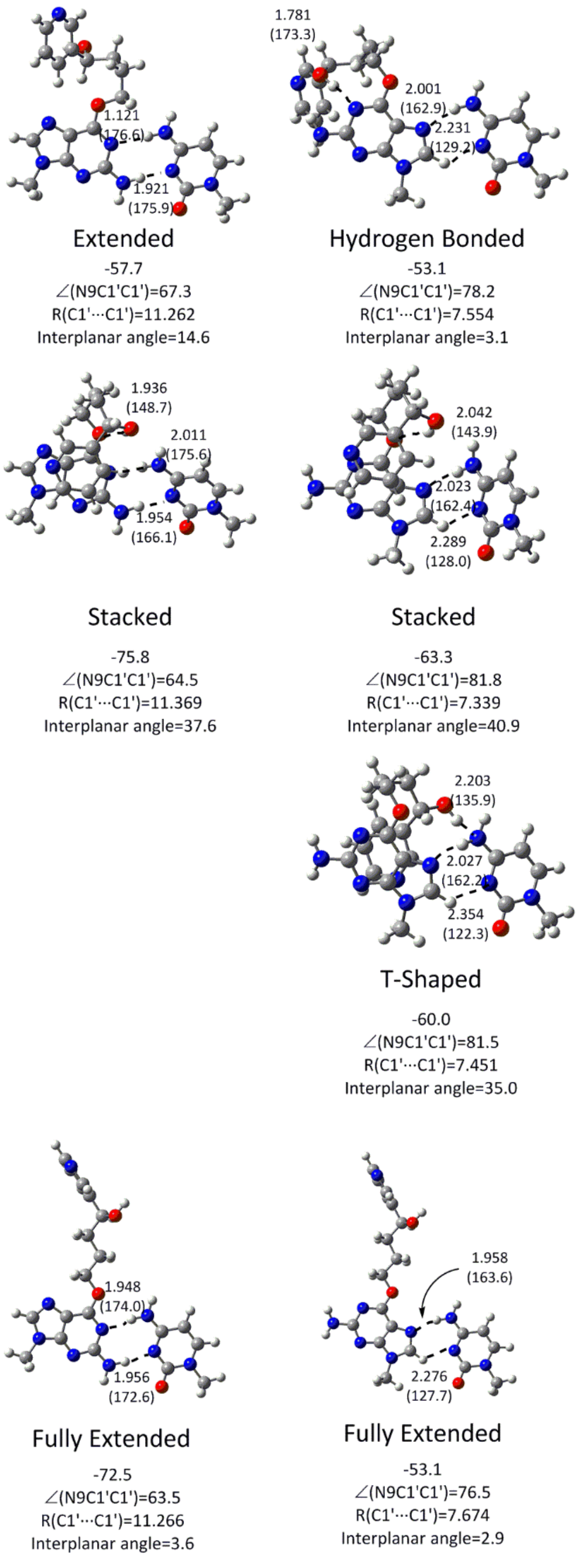

Figure S6. B3LYP-D3(BJ)/6-311+G(2df,2p)//M06-2X/6-31G(d) structures (distances in Å and angles in deg.) and binding energy (kJ/mol) for dimers between the Watson-Crick (left) or Hoogsteen (right) face of various PHB-G conformations and C.

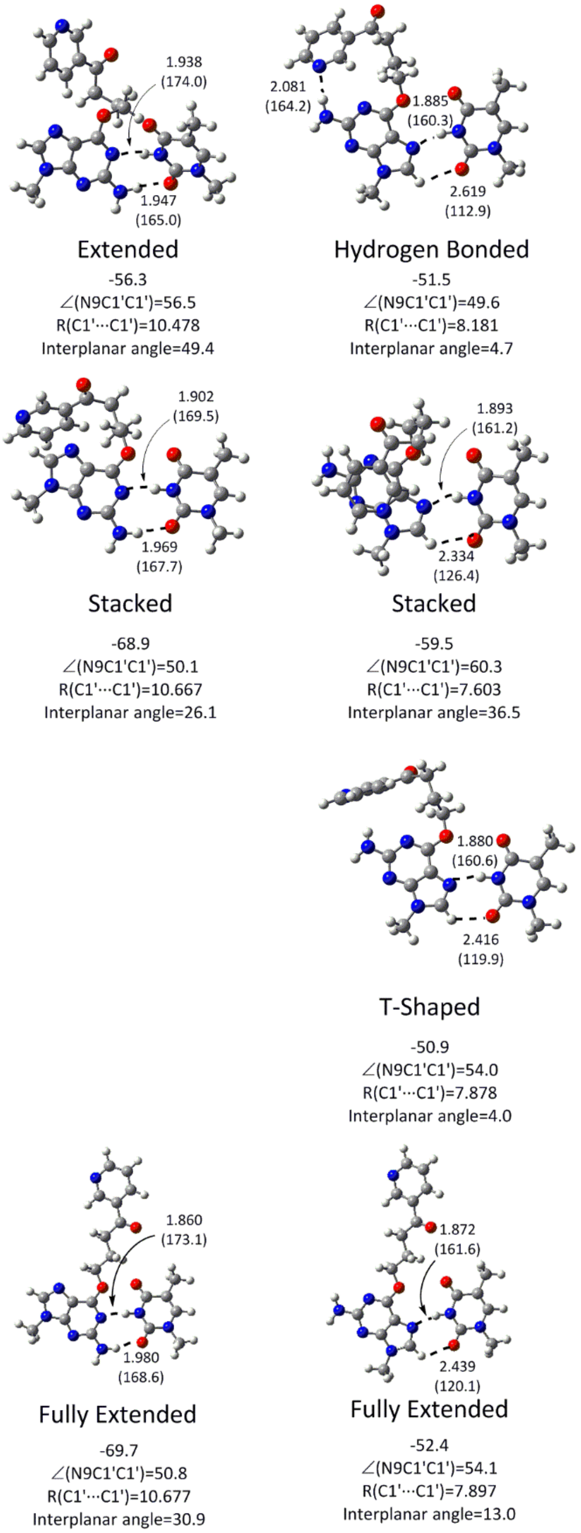

Figure S7. B3LYP-D3(BJ)/6-311+G(2df,2p)//M06-2X/6-31G(d) structures (distances in Å and angles in deg.) and binding energy (kJ/mol) for dimers between the Watson-Crick (left) or Hoogsteen (right) face of various POB-G conformations and T.

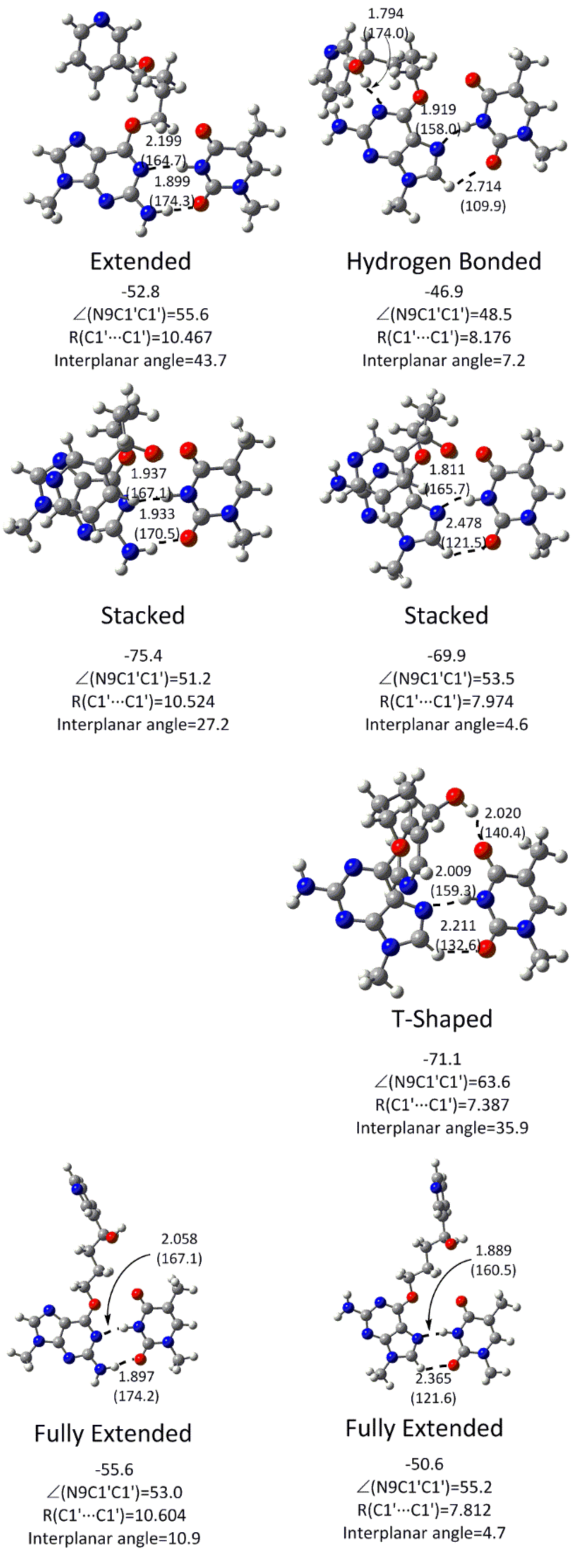

Figure S8. B3LYP-D3(BJ)/6-311+G(2df,2p)//M06-2X/6-31G(d) structures (distances in Å and angles in deg.) and binding energy (kJ/mol) for dimers between the Watson-Crick (left) or Hoogsteen (right) face of various PHB-G conformations and T.

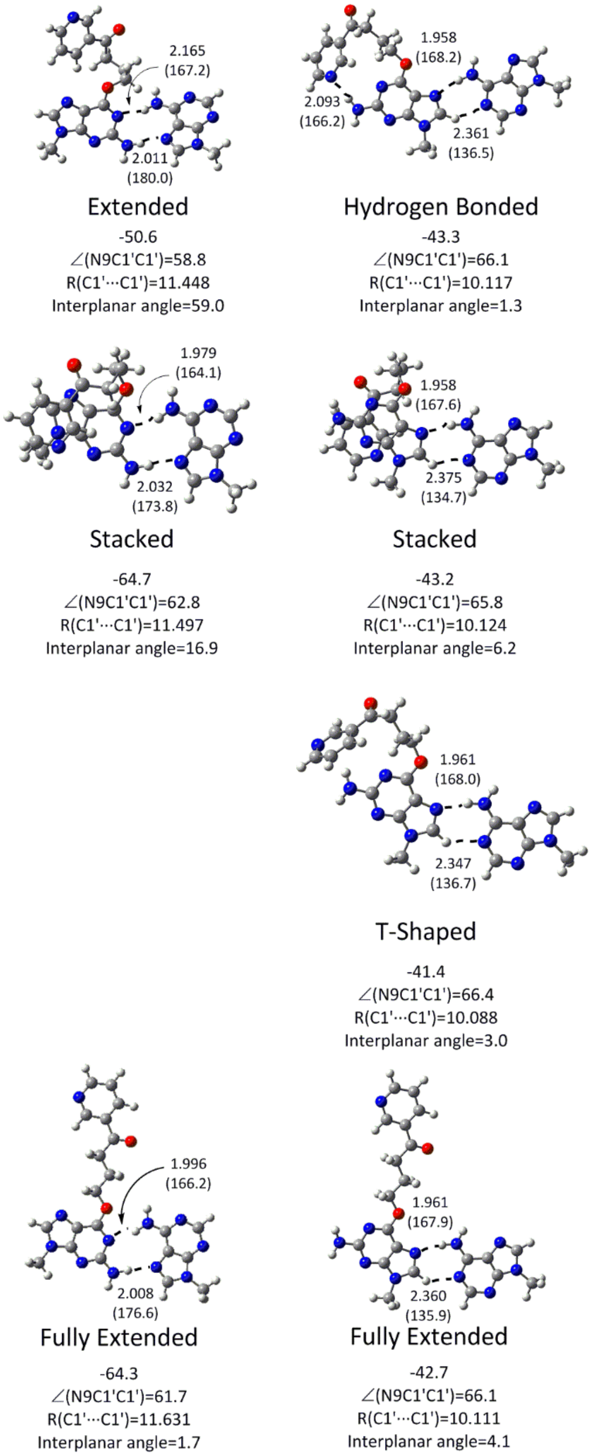

Figure S9. B3LYP-D3(BJ)/6-311+G(2df,2p)//M06-2X/6-31G(d) structures (distances in Å and angles in deg.) and binding energy (kJ/mol) for dimers between the Watson-Crick (left) or Hoogsteen (right) face of various POB-G conformations and A.

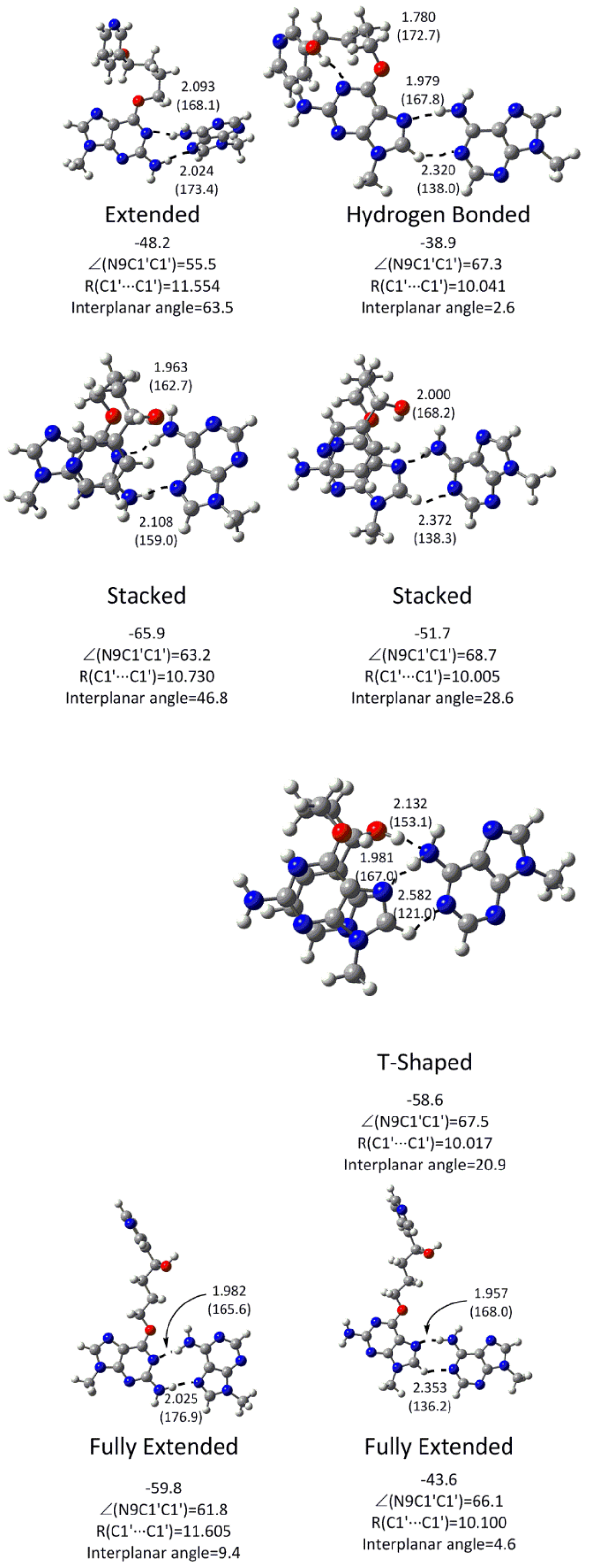

Figure S10. B3LYP-D3(BJ)/6-311+G(2df,2p)//M06-2X/6-31G(d) structures (distances in Å and angles in deg.) and binding energy (kJ/mol) for dimers between the Watson-Crick (left) or Hoogsteen (right) face of various PHB-G conformations and A.

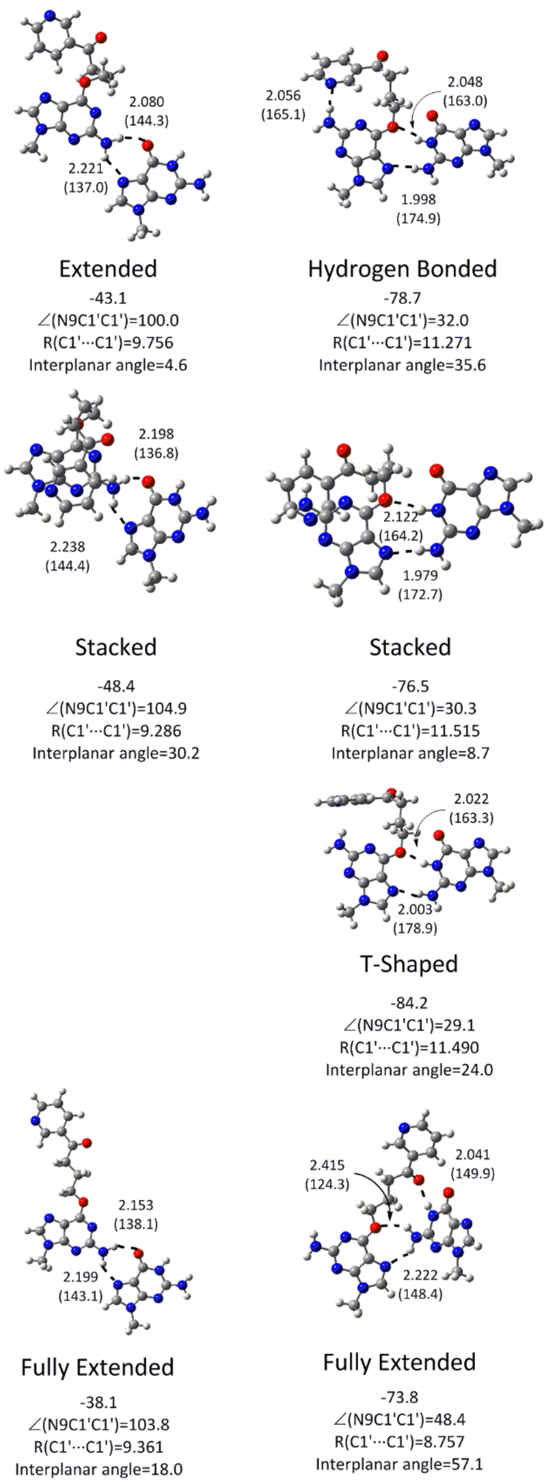

Figure S11. B3LYP-D3(BJ)/6-311+G(2df,2p)//M06-2X/6-31G(d) structures (distances in Å and angles in deg.) and binding energy (kJ/mol) for dimers between the Watson-Crick (left) or Hoogsteen (right) face of various POB-G conformations and G.

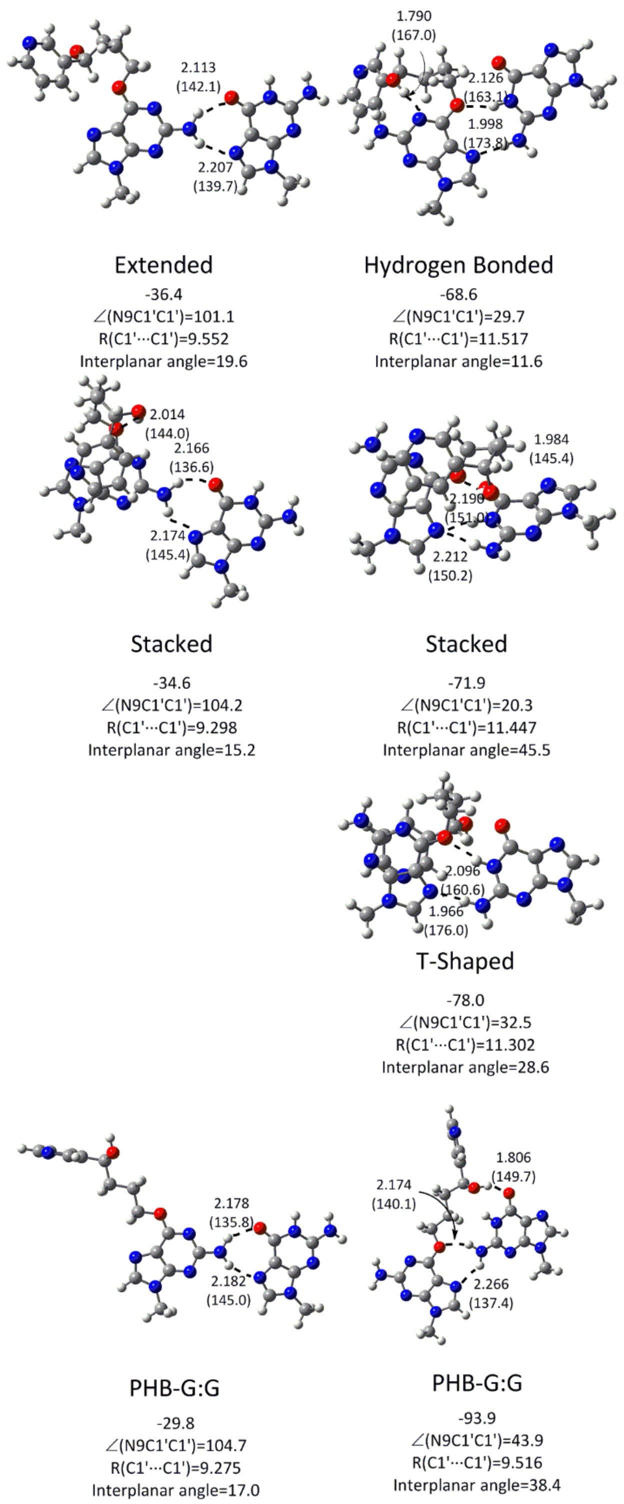

Figure S12. B3LYP-D3(BJ)/6-311+G(2df,2p)//M06-2X/6-31G(d) structures (distances in Å and angles in deg.) and binding energy (kJ/mol) for dimers between the Watson-Crick (left) or Hoogsteen (right) face of various PHB-G conformations and G.

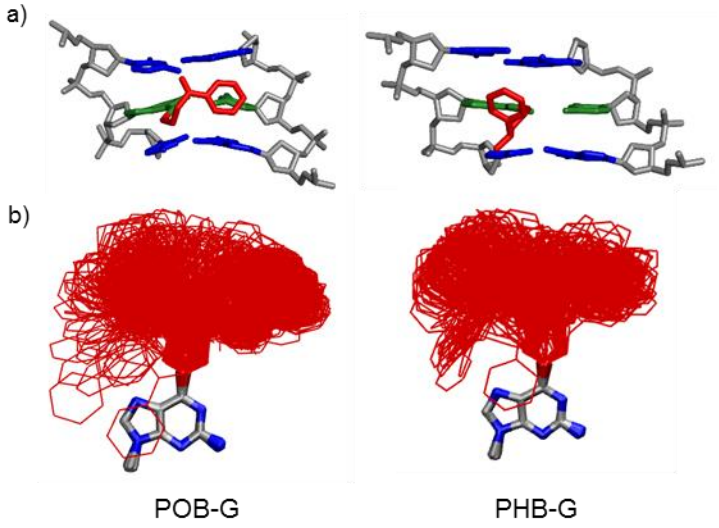

Figure S13. a) MD representative structures obtained for POB-G (left) and PHB-G (right) adducted DNA based on an initial stacked lesion conformation opposite C. b) Overlay (based on G ring atoms) of lesion conformations adopted throughout the MD simulation, highlighting the deviation in bulky moiety orientation (red).

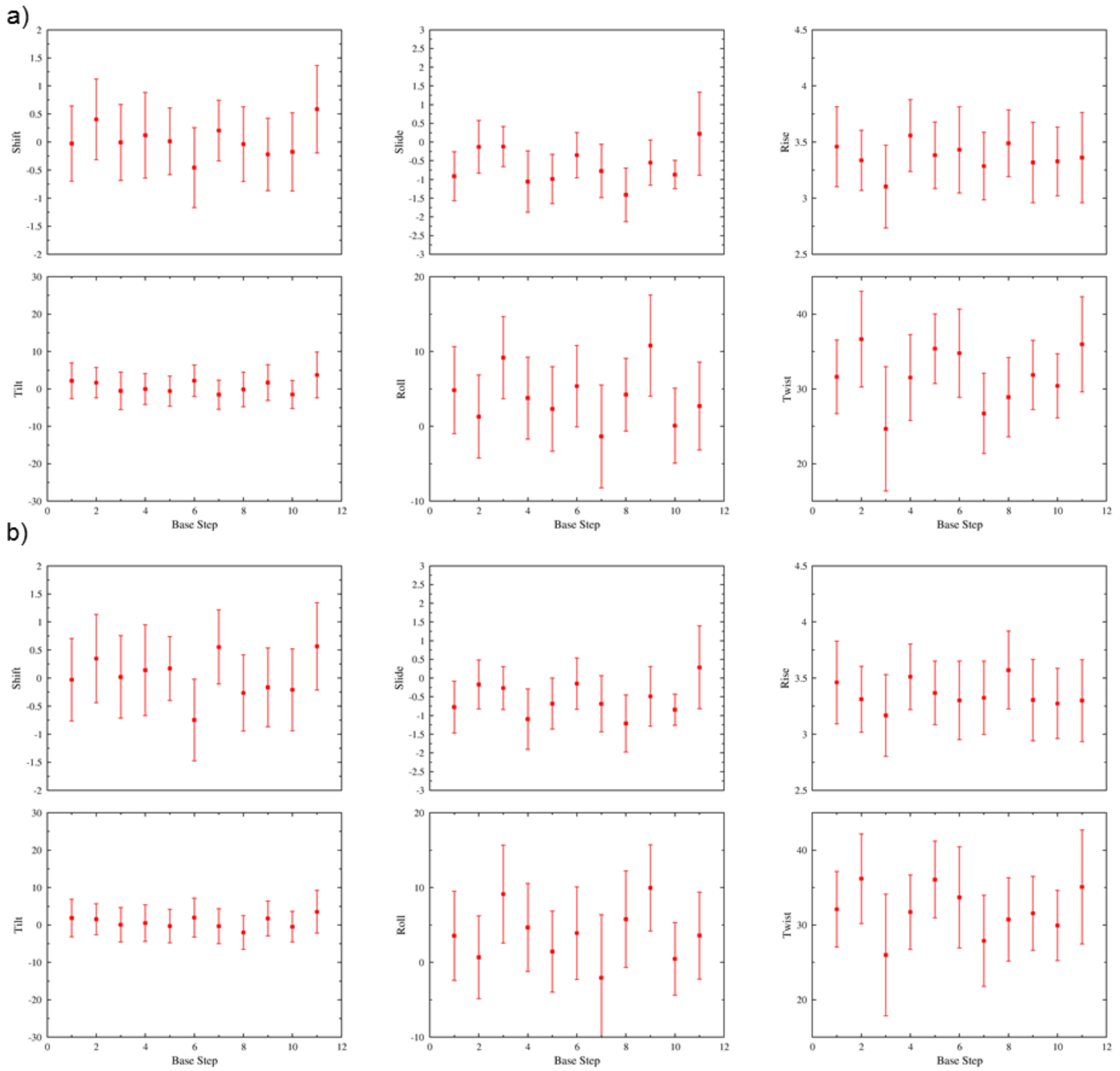

Figure S14. Average base step parameters from MD simulations on a) POB-G or b) PHB-G adducted DNA with the lesion in the extended conformation paired opposite C.

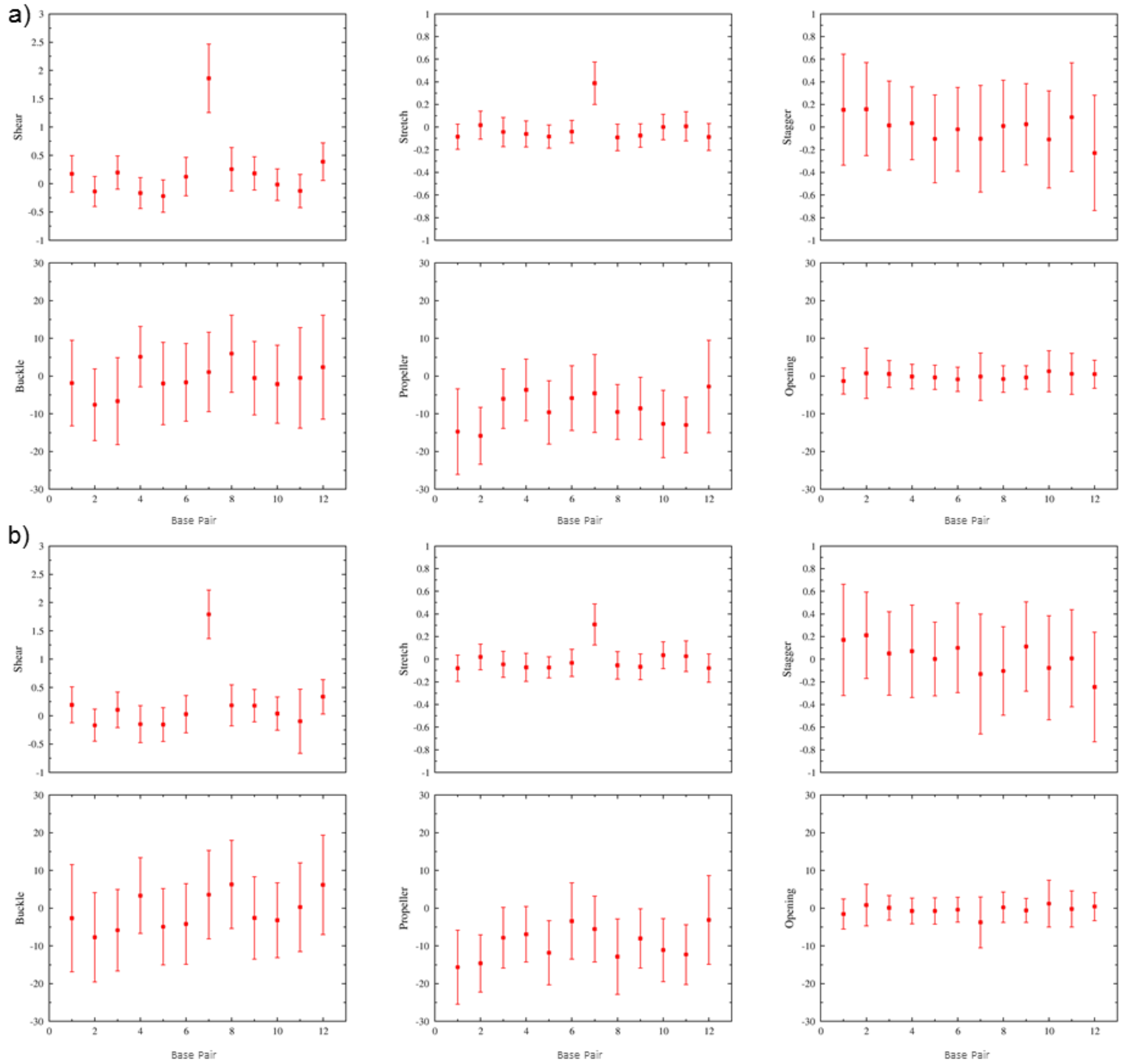

Figure S15. Average base pair parameters for a) POB-G or b) PHB-G adducted DNA with the lesion in the extended conformation paired opposite C.

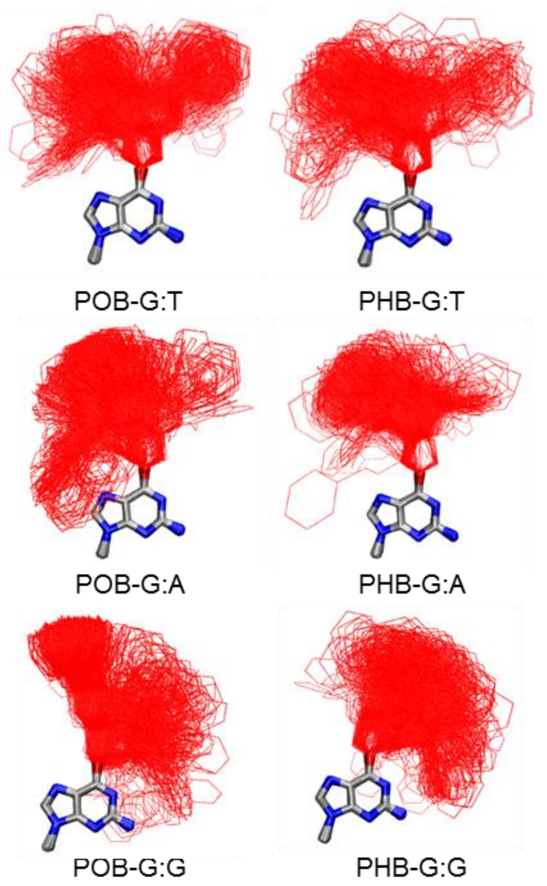

Figure S16. Overlay (based on G ring atoms) of lesion conformations adopted throughout MD simulations on DNA containing POB-G(left) or PHB-G (right) paired opposite T (top), A (middle) and G (bottom), highlighting the deviation in bulky moiety orientation (red).

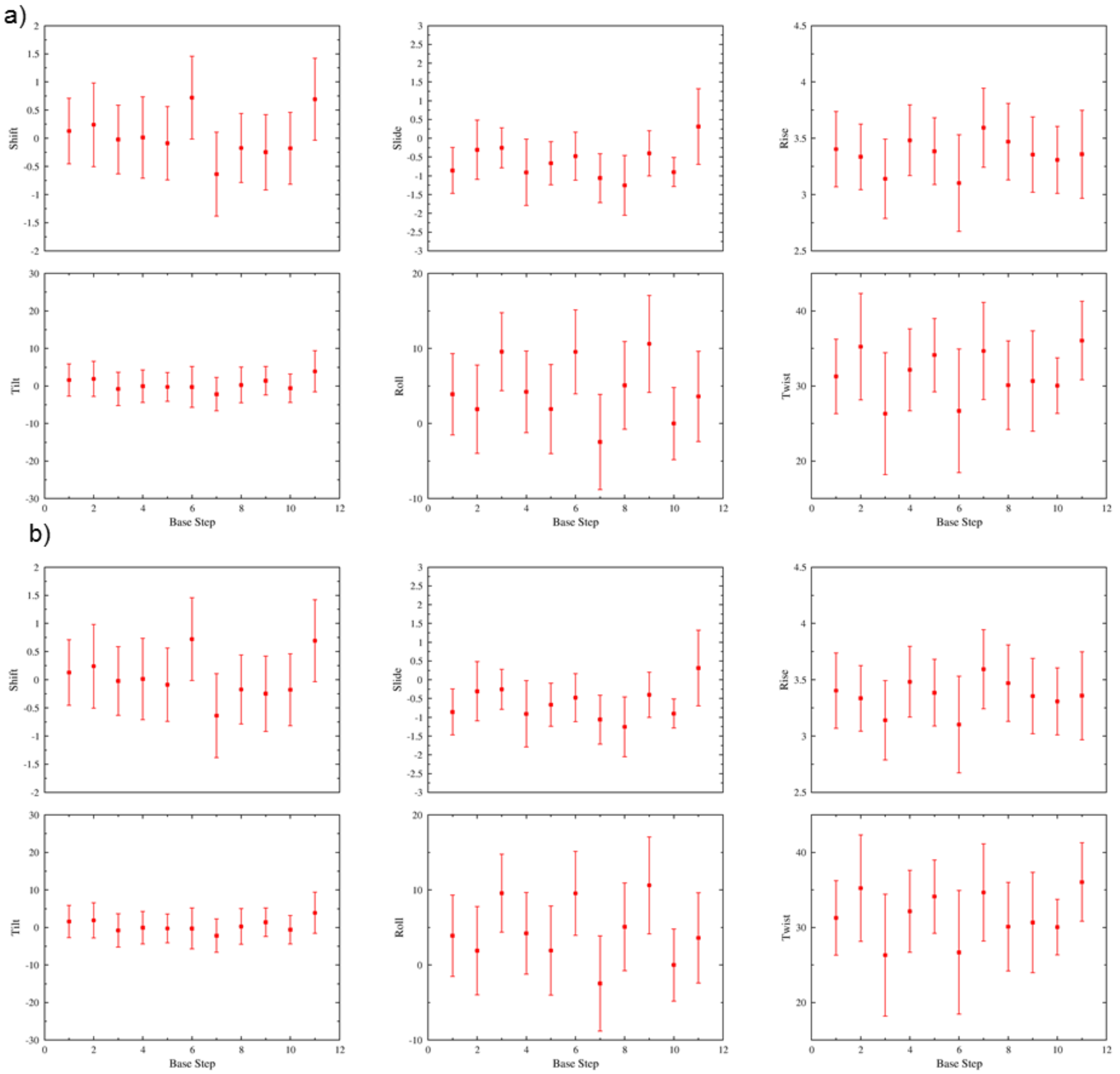

Figure S17. Average base step parameters from MD simulations on a) POB-G or b) PHB-G adducted DNA with the lesion in the extended conformation paired opposite T.

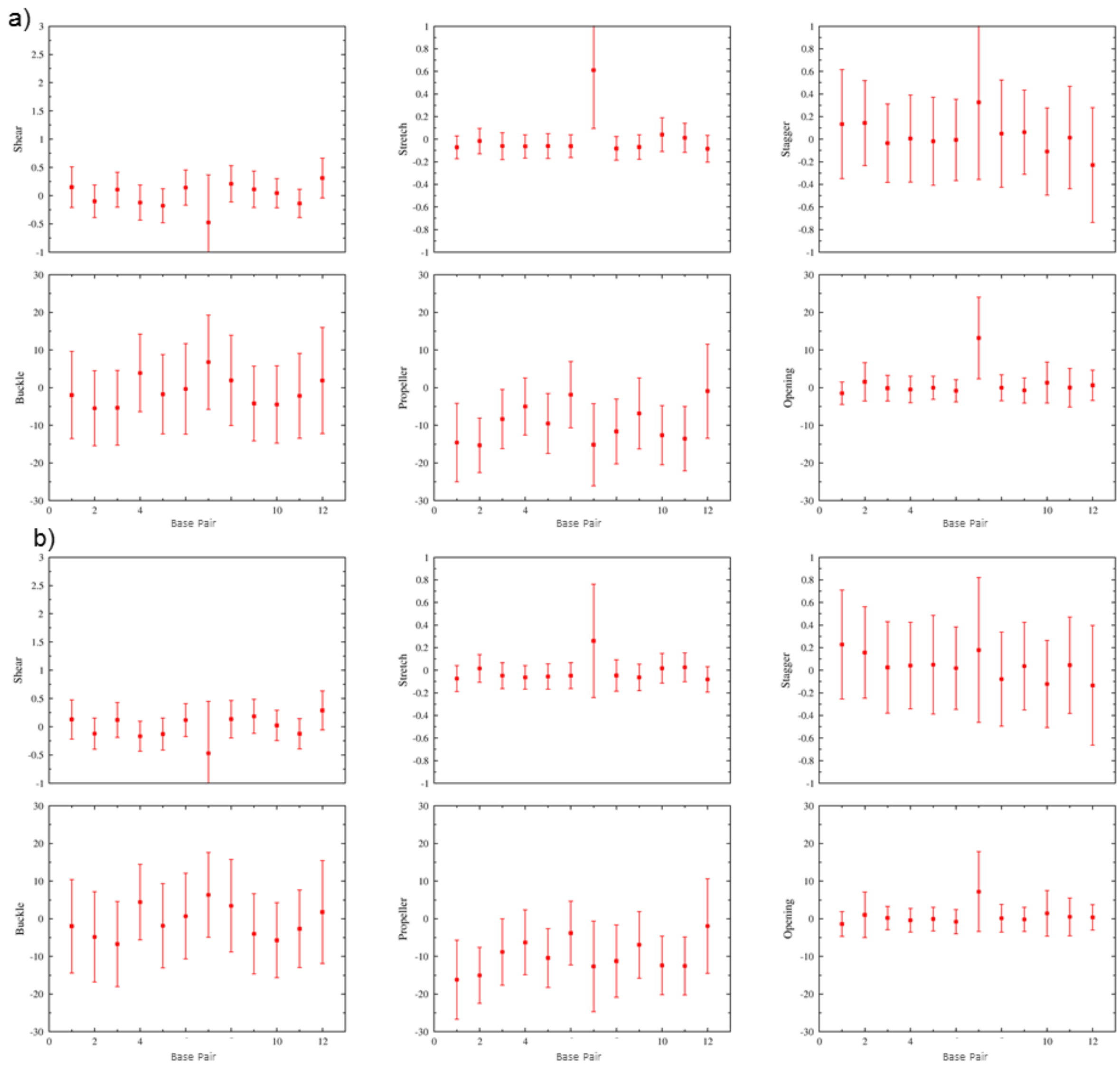

Figure S18. Average base pair parameters for a) POB-G or b) PHB-G adducted DNA with the lesion in the extended conformation paired opposite T.

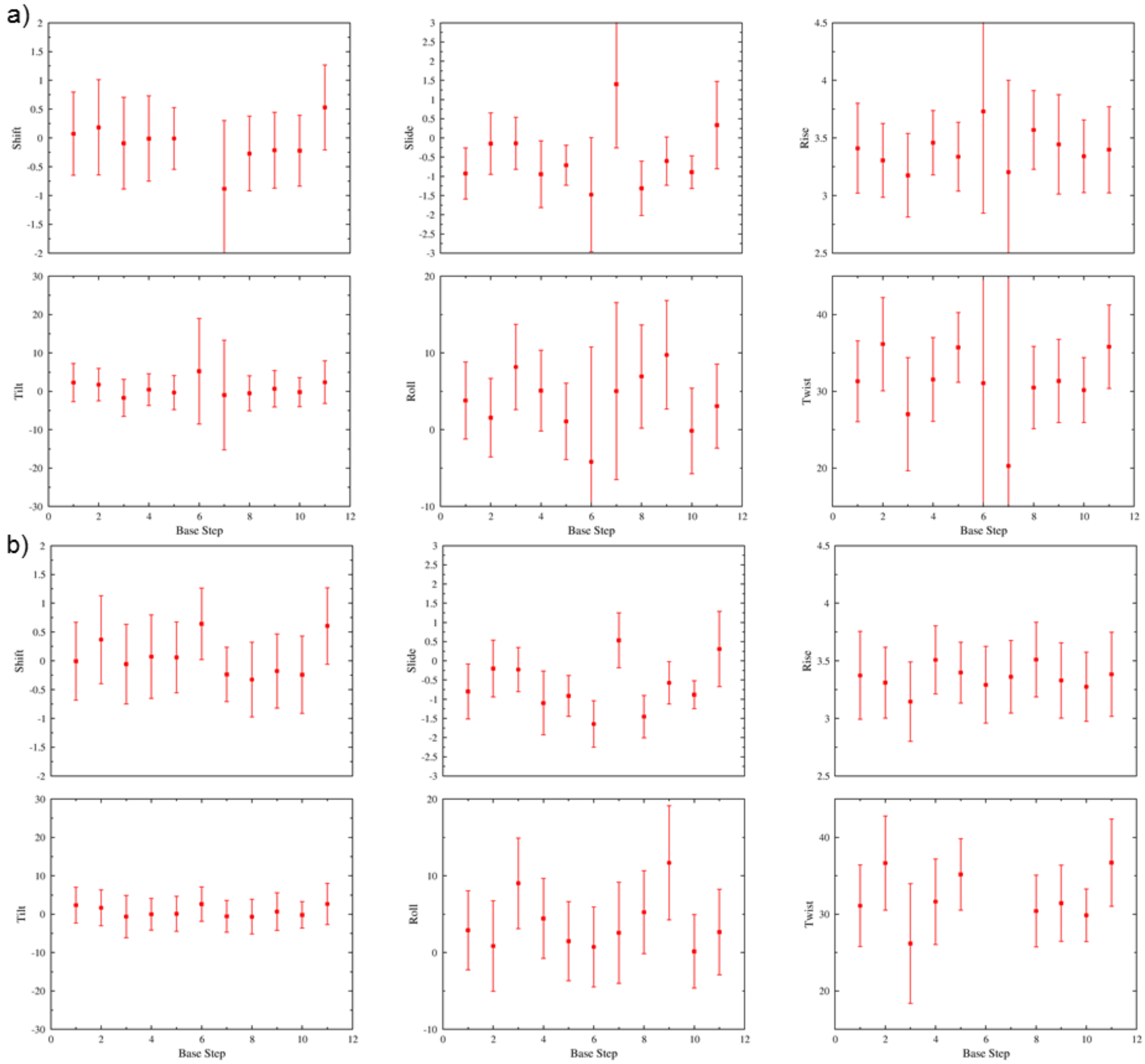

Figure S19. Average base step parameters for a) POB-G or b) PHB-G adducted DNA with the lesion in the extended conformation paired opposite A.

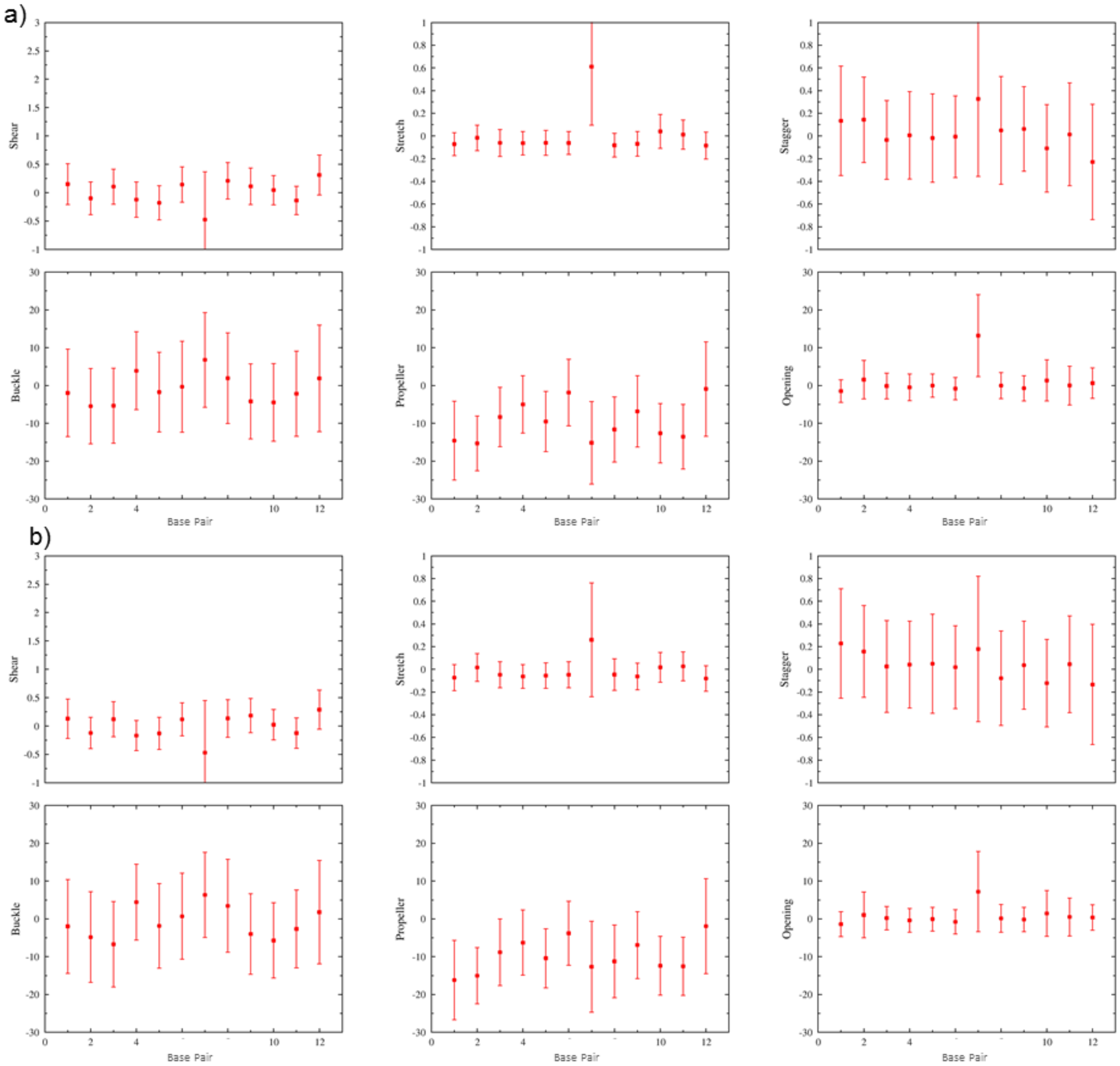

Figure S20. Average base pair parameters for a) POB-G or b) PHB-G adducted DNA with the lesion in the extended conformation paired opposite A.

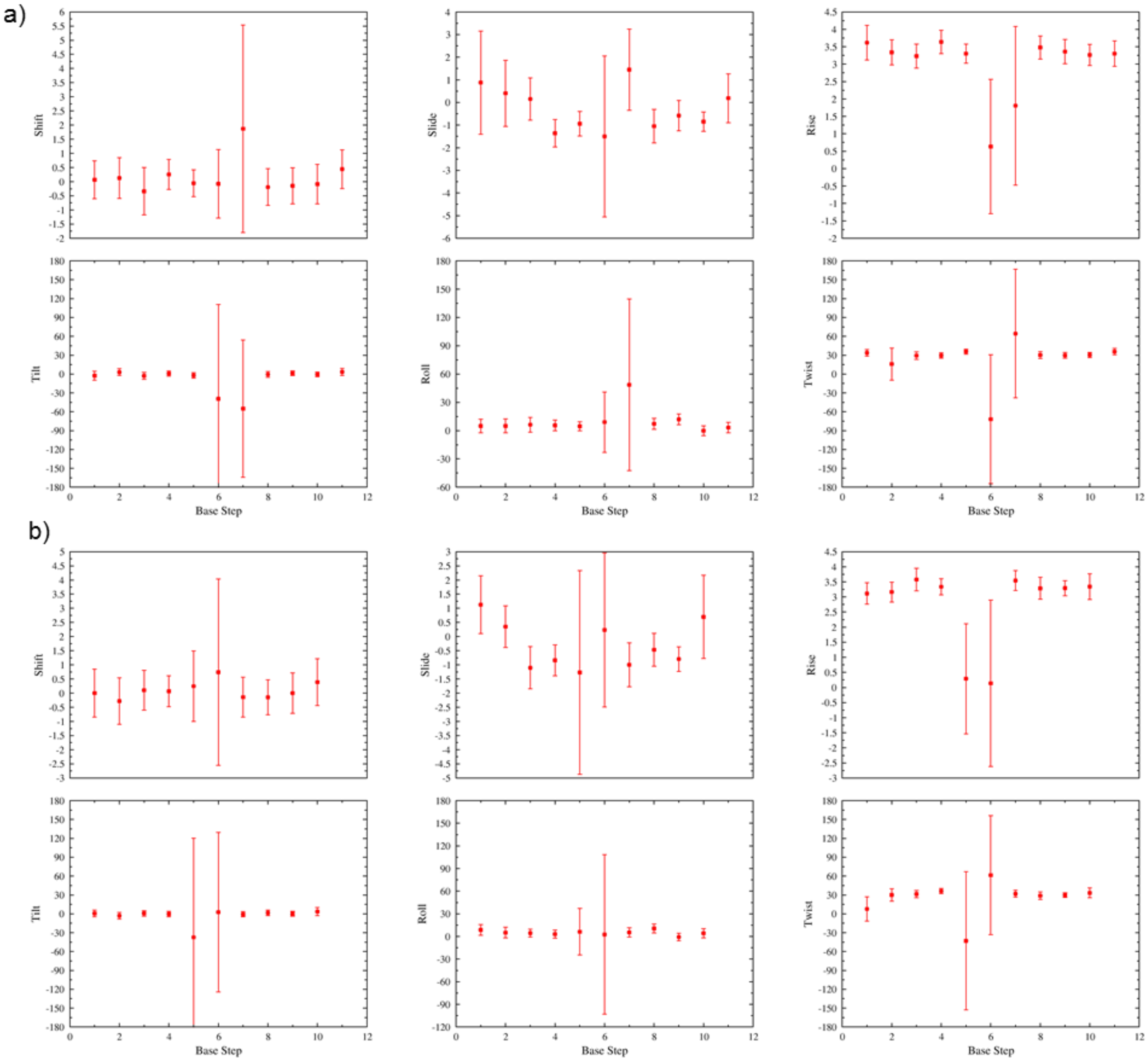

Figure S21. Average base step parameters for a) POB-G or b) PHB-G adducted DNA with the lesion in the extended conformation paired opposite G.

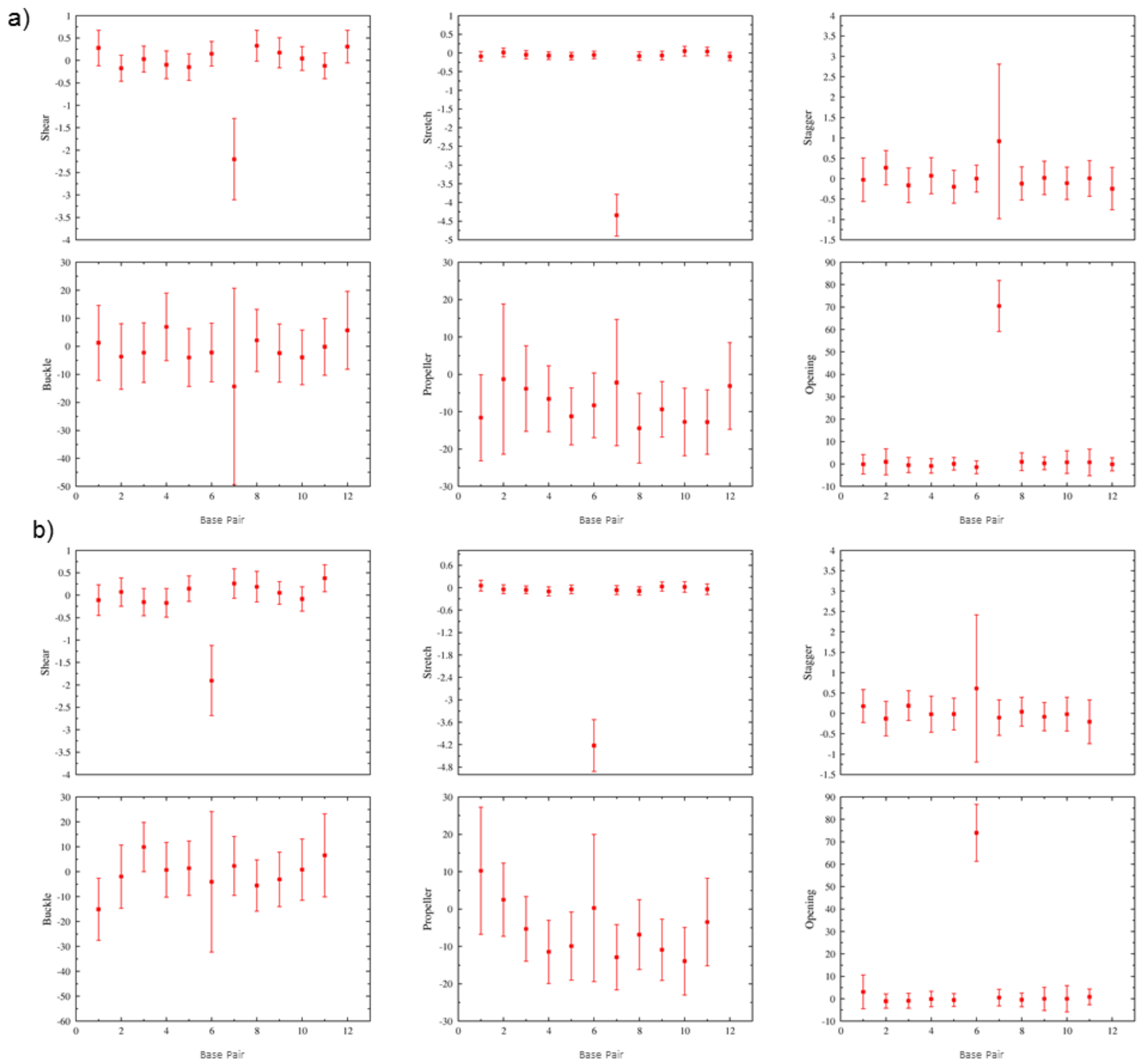

Figure S22. Average base pair parameters for a) POB-G or b) PHB-G adducted DNA with the lesion in the extended conformation paired opposite G.

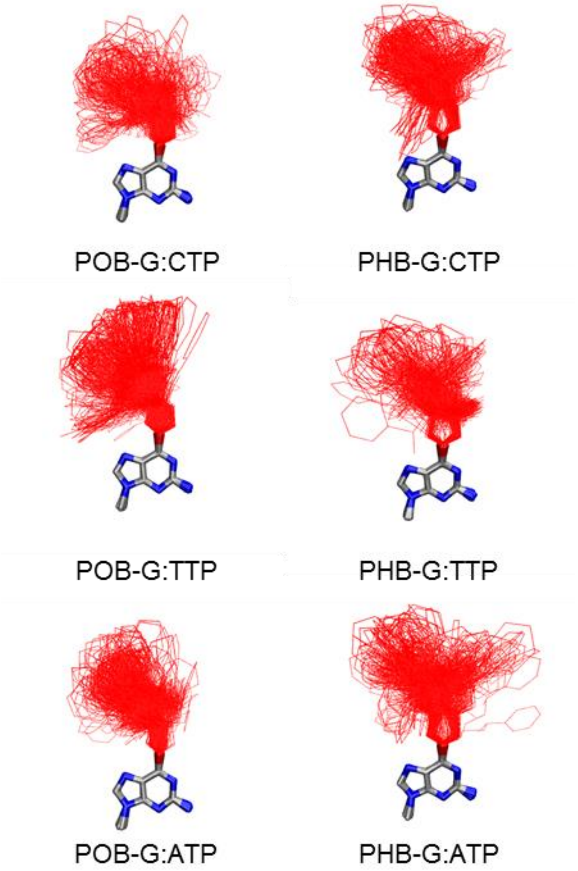

Figure S23. Overlay (based on G ring atoms) of lesion conformations adopted throughout the MD simulations on polymerase η complexes corresponding to dCTP (top), dTTP (middle), or dATP (bottom) insertion opposite POB-G (left) or PHB-G (right), highlighting the deviation in bulky moiety orientation (red).

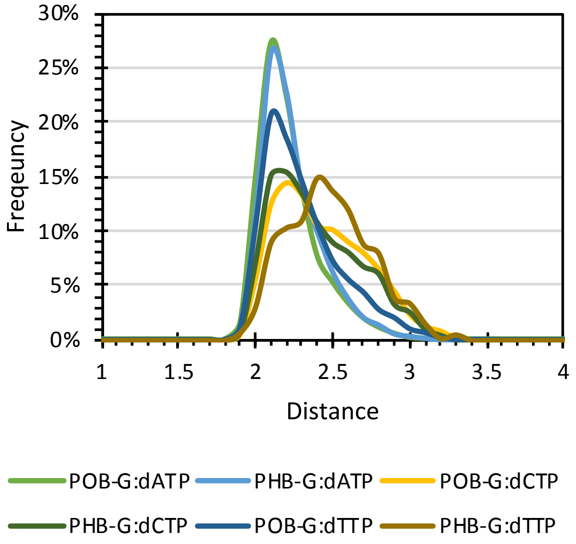

Figure S24. Distance between the binding Mg^{2+} ion and coordinating Oα during MD simulations on the insertion of a dNTP opposite POB-G or PHB-G by polymerase η. Full coordination was considered to occur if the distance was < 2.5 Å for > 95% of the simulation.

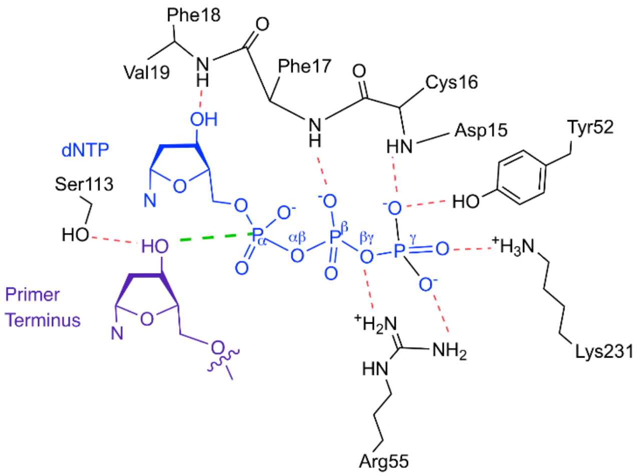

Figure S25. Hydrogen-bonding interactions with the dNTP in the polymerase η active site observed in the crystal structure corresponding to dATP incorporation opposite T (PDB ID: 4ECS).
